# Discovery of Natural Dimeric Naphthopyrones as Potential Cytotoxic Agents through ROS-Mediated Apoptotic Pathway

**DOI:** 10.3390/md17040207

**Published:** 2019-04-02

**Authors:** Kuo Xu, Chuanlong Guo, Jie Meng, Haiying Tian, Shuju Guo, Dayong Shi

**Affiliations:** 1 Chinese Academy of Sciences Key Laboratory of Experimental Marine Biology, Institute of Oceanology, Chinese Academy of Sciences, Qingdao 266071, China; xukuoworld@126.com (K.X.); gcl_cpu@126.com (C.G.); mengjie@qibebt.ac.cn (J.M.); 2 Department of Pharmacy, College of Chemical Engineering, Qingdao University of Science and Technology, Qingdao 266042, China; 3 College of Resources and Environment, Qingdao Agricultural University, Qingdao 266109, China; 4 Technology Center, China Tobacco Henan Industrial Co., Ltd., Zhengzhou, 450000, China; 13623810925@126.com; 5 State Key Laboratory of Microbial Technology, School of Life Science, Shandong University, No. 72 Binhai Road, Qingdao 266237, China

**Keywords:** *Aspergillus*, naphthopyrones, cytotoxicity, endophytic fungus, *Leathesia nana*

## Abstract

A study on the secondary metabolites of *Aspergillus sp*. XNM-4, which was derived from marine algae *Leathesia nana* (Chordariaceae), led to the identification of one previously undescribed (**1**) and seventeen known compounds (**2**–**18**). Their planar structures were established by extensive spectroscopic analyses, while the stereochemical assignments were defined by electronic circular dichroism (ECD) calculations. The biological activities of the compounds were assessed on five human cancer cell lines (PANC-1, A549, MDA-MB-231, Caco-2, and SK-OV-3), and one human normal cell line (HL-7702) using an MTT [3-(4,5-dimethyl-2-thiazolyl)-2,5-diphenyl tetrazolium bromide] assay. Among them, the dimeric naphthopyrones **7**, **10** and **12** exhibited potent cytotoxicity. Further mechanism studies showed that **12** induced apoptosis, arrested the cell cycle at the G0/G1 phase in the PANC-1 cells, caused morphological changes and generated ROS; and it induces PANC-1 cells apoptosis via ROS-mediated PI3K/Akt signaling pathway.

## 1. Introduction

Marine-derived endophytic fungi have drawn considerable attention for their surprising potential in drug discovery [1,2,3,4,5]. These endophytic fungi can be distributed in every possible marine host, such as plants, invertebrates and vertebrates [6]. In the interactional process of symbiosis and evolution, the host provides suitable living conditions to the endophytes, while the endophytes contribute bioactive secondary metabolites that provide protection and, ultimately, survival value to their hosts [7,8]. As one of the most prevalent sources of microorganisms, marine algae offer an abundant amount of endophytic fungi for chemical studies. Hundreds of natural products have been identified from the algal-derived fungi [9].

**Figure 1:**
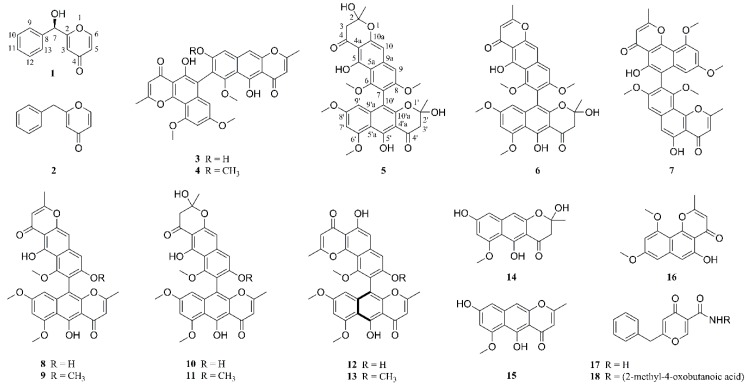
Compounds **1**–**18** isolated from *Aspergillus sp*. XNM-4.

In our ongoing efforts to discover the bioactive secondary metabolites of endophytic fungi from the marine brown algae *Leathesia nana* (Chordariaceae), eighteen compounds were isolated from an *Aspergillus sp*. XNM-4 strain (Figure 1). The planar structures of the metabolites were established by HRESIMS, UV, IR, one- and two-dimensional (1D and 2D) NMR spectroscopic data, while the stereochemistry of compounds **1** and **12** were assigned by a comparison of the calculated and experimental electronic circular dichroism (ECD). All compounds were assessed for inhibitory effects on five human cancer cell lines (PANC-1, A549, MDA-MB-231, Caco-2, and SK-OV-3) and one human normal cell line (HL-7702). Notably, as the most promising candidate, the cytotoxic mechanism of compound **12** in PANC-1 cells was studied preliminarily. These experimental results may be beneficial for the development of naturally occurring dimeric naphthopyrones as anti-tumor agents.

## 2. Results and Discussion

### 2.1. Structural Elucidation

Compound **1** was isolated as a white amorphous powder. Its molecular formula (C_12_H_10_O_3_) was determined on the HRESIMS (*m*/*z* 203.0707 [M + H]^+^, calcd 203.0708, Figure S3) in association with ^13^C NMR data. The IR spectrum (Figure S4) exhibited absorption bands for carbonyl (1653 cm^−1^) and olefinic (1384, 1602 cm^−1^) groups. The ^1^H NMR data of **1** (Table 1, Figure S5) revealed an ABX spin-system assignable to three pyrone protons [*δ*_H_ 8.05 (^1^H, d, *J* = 6.0 Hz), 6.21 (^1^H, dd, *J* = 2.4, 6.0 Hz), and 6.42 (^1^H, d, *J* = 2.4 Hz)]; however, their coupling constants were different from those of the benzene ring. The ^1^H NMR spectrum also showed five pyrone protons [*δ*_H_ 7.41 (2H, overlap), 7.37 (2H, overlap), and 7.32 (^1^H, m)] and an oxygenated methyne *δ*_H_ 5.48 (^1^H, s). In combination with the five resonance peaks at *δ*_H_ 7.32−7.41, the pyrone resonances in the ^13^C NMR spectrum [*δ*_C_ 140.6 (1C), 128.4 (2C), 128.0 (1C), and 126.8 (2C), Figure S6] supported the existence of a mono-substituted benzene ring [10]. The remaining five resonance peaks [*δ*_C_ 177.9 (1C), 170.2 (1C), 156.2 (1C), 116.2 (1C), and 112.3 (1C)] indicated a skeleton of 4*H*-pyran-4-one [11], except for the oxygenated carbon resonance at *δ*_C_ 71.2 (1C). In the 2D NMR experiment (Figures S7 and S8), the hydrogen resonance at *δ*_H_ 6.21 (H-5) has a homonuclear correlation with the resonance at *δ*_H_ 8.05 (H-6), and the hydrogen resonance at *δ*_H_ 5.48 (H-7) has long-range heteronuclear correlations with the carbon resonances at *δ*_C_ 112.3 (C-3) and 126.8 (C-9, 13), which confirmed that a benzyl group was substituted at C-2. Thus, the structure of compound **1** was identified as (hydroxy(phenyl)methyl)-4*H*-pyran-4-one.

**Figure 2:**
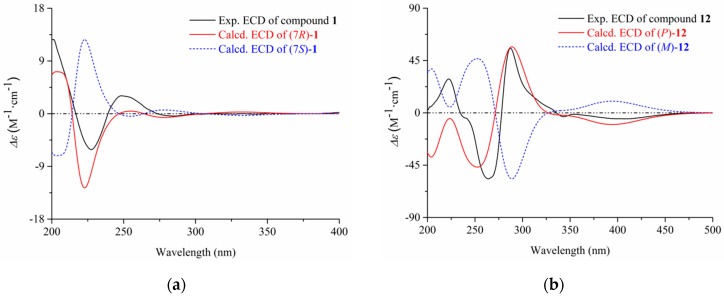
The experimental and calculated ECD of compounds **1** (**a**) and **12** (**b**).

The configurational assignment of C-7 was defined by ECD calculations using a MMFF94 force field and time-dependent density functional theory (TDDFT) at the B3LYP/6-311+G(d, p) level. The overall calculated ECD curve of (7*R*)-**1** were produced by Boltzmann weighting of their lowest energy conformers, matching well with the corresponding experimental ECD data (Figure 2, the procedure was detailed in Supplementary Materials, S34–S36). Thus, the structure of compound **1** was finally established as (7*R*)-(hydroxy(phenyl)methyl)-4*H*-pyran-4-one.

By comparing the spectroscopic data (HRESIMS, ^1^H and ^13^C NMR, Figures S9–S60) with those reported in the literature, the remaining nineteen known compounds (**2**–**21**) were identified as 2-benzyl-4*H*-pyran-4-one (**2**) [11], asperpyrone D (**3**) [12], asperpyrone C (**4**) [13], aurosperone B (**5**) [14], fonsecinone B (**6**) [14], asperpyrone B (**7**) [15], dianhydro-aurasperone C (**8**) [12], isoaurasperone A (**9**) [15], aurasperone F (**10**) [16], fonsecinone D (**11**) [14], asperpyrone A (**12**) [12], fonsecinone A (**13**) [15], fonsecin (**14**) [14], TMC 256 A1 (**15**) [17], flavasperone (**16**) [14], carbonarone A (**17**) [18], pestalamide A (**18**) [19]. In addition, the *p* configuration of **12** was defined by ECD calculation at the B3LYP/6-311+G(d, p) level (Figure 2, Supplementary Materials, S34–S36).

### 2.2. Cytotoxic Activities of Compounds ***1***–***18***

**Figure 3:**
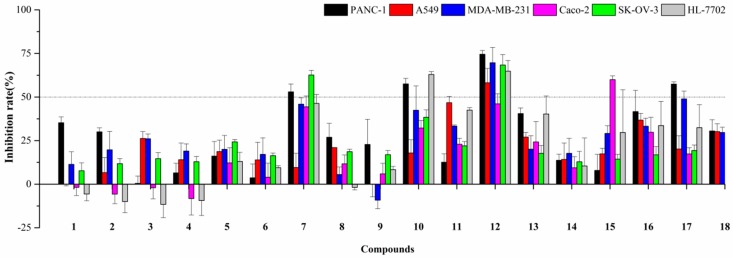
Cytotoxic activities of compounds **1**–**18** at the concentration of 50 µM.

**Figure 4:**
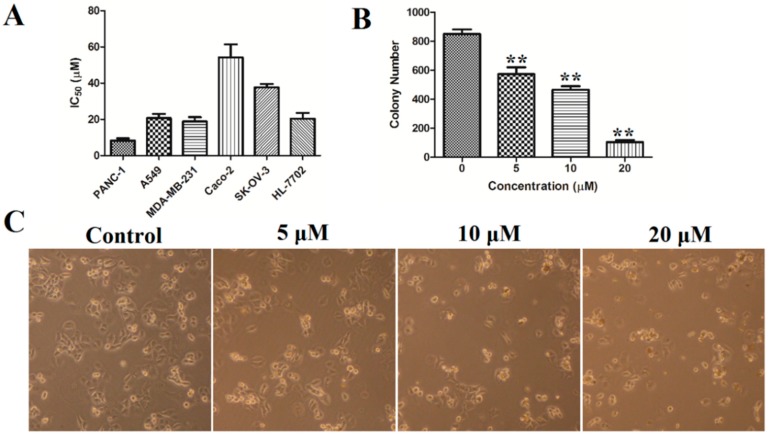
(**A**) The IC_50_ values of compound **12** on PANC-1, A549, MDA-MB-231, Caco-2, SK-OV-3, and HL-7702 cells; (**B**) colony formation in PANC-1 cells was determined by staining with crystal violet; (**C**) cell morphology was observed using inverted microscope. ** *p* < 0.01 vs. control group.

Natural naphthopyrones have been previously reported for their anticancer potential [13,15,17]. Therefore, the present study evaluated the inhibitory effects of the isolated compounds on five human cancer cell lines (PANC-1, A549, MDA-MB-231, Caco-2, and SK-OV-3), and one human normal cell line (HL-7702) at a concentration of 50 µM. As a result, the dimeric naphthopyrones **7**, **10**, and especially **12**, exhibited potent cytotoxicity on PANC-1, A549, MDA-MB-231, Caco-2, SK-OV-3 and HL-7702 cells (Figure 3). The IC_50_ values of compound **12** on the different cells were further measured, and it possessed the greatest inhibitory effects against PANC-1, with an IC_50_ value of 8.25±2.20 µM (Figure 4A).

### 2.3. Pharmacological Mechanism of Compound 12 on PANC-1 Cells

#### 2.3.1. Morphological Changes

It is well known that cytotoxic agents often cause changes in cell morphology, such as irregular cell morphology, increased cell debris, and reduced cell numbers. As shown in Figure 4, after treatment with compound **12**, the PANC-1 cells showed morphological changes such as cell shrinkage, deformation and a reduced number of viable cells.

#### 2.3.2. Colony Formation

A 10-day colony formation experiment was performed to explore the long-term impact of compound **12** on the PANC-1 cells growth. PANC-1 cells were seeded in 6-well plates (1000 cells/well) and were treated with various concentrations of compound **12** (0, 5, 10, 20 µM) for 10 days to allow colony formation. As shown in Figure 4B, 852 ± 43 colonies were present in the control group, whereas the colony numbers decreased to 574 ± 65, 421 ± 30 and 105 ± 21 after treatment with compound **12** (5, 10, and 20 μM, respectively). These results showed that compound **12** could inhibit the colony formation of PANC-1 cells.

#### 2.3.3. Cell Apoptosis

**Figure 5:**
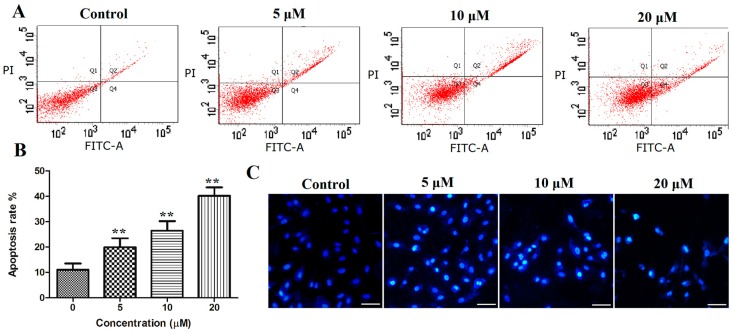
(**A**,**B**) PANC-1 cells were stained with Annexin-V FITC and propidium iodide (PI), and then analyzed using flow cytometry, both early and late apoptotic cells were analyzed; (**C**) PANC-1 cells were stained with Hoechst 33258 and photographed using a fluorescence microscopy (bar = 50 μm). ** *p* < 0.01 vs. control group.

To explore whether the abovementioned reduction in cell viability was caused by the induction of apoptosis, PANC-1 cells were treated with compound **12** (5, 10, and 20 μM) for 72 h. The cells were then stained with fluorescein isothiocyanate (Annexin-V FITC) and propidium iodide (PI) and were analyzed by flow cytometry. The results indicated that compound **12** could induce cell apoptosis in a concentration-dependent manner. As shown in Figure 5A,B, 11.07 ± 2.43% of the apoptotic cells were present in the control, whereas the apoptotic population increased to 19.93 ± 65, 26.43 ± 3.81 and 40.43 ± 3.27 after treatment with **12** (5, 10, and 20 μM, respectively).

Apoptosis often causes morphological changes, which can be observed by Hoechst 33258 staining the apoptotic cells. Thus, the PANC-1 cells were treated with compound **12** (5, 10, and 20 μM) for 72 h, stained with Hoechst 33258 and analyzed by fluorescence microscopy; significant morphological changes were observed. As shown in Figure 5C, nuclear pyknosis and chromosome condensation were observed in PANC-1 treated with compound **12**, and no apoptosis was found in the control group.

#### 2.3.4. Cell Cycle

**Figure 6:**
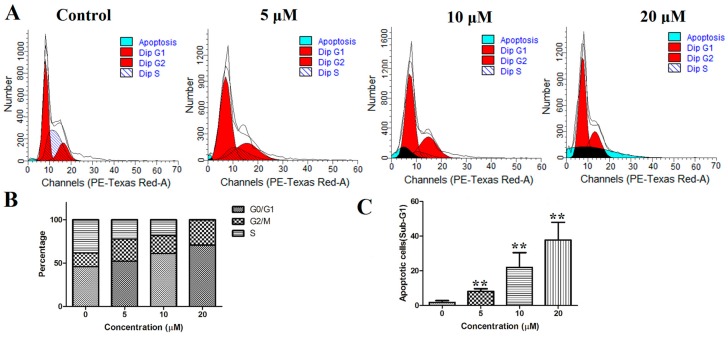
(**A**) PANC-1 cells were stained with propidium iodide (PI) and analyzed using flow cytometry; (**B**) proportion of PANC-1 cells in each phase; (**C**) the sub-G1 was increased after treatment of compound **12**. ** *p* < 0.01 vs. control group.

To explore the influence of this compound on the cell cycle distribution, PANC-1 cells were treated with compound **12** (5, 10, and 20 μM) for 72 h. Next, the cell cycle distribution was analyzed by flow cytometry after staining with PI. As shown in Figure 6A,B, the G0/G1 phase was increased in a concentration-dependent manner in the PANC-1 cells. Compared with the control group, the population in the G1 phase increased from 45.97% to 70.94% at a concentration of 20 µM. Moreover, the sub-G1 group significantly increased after the cells were cultured with compound **12** (Figure 6C). These results indicated that compound **12** could induce apoptosis and arrested the cell cycle at the G0/G1 phase in PANC-1 cells in a concentration-dependent manner.

#### 2.3.5. ROS Generation

**Figure 7:**
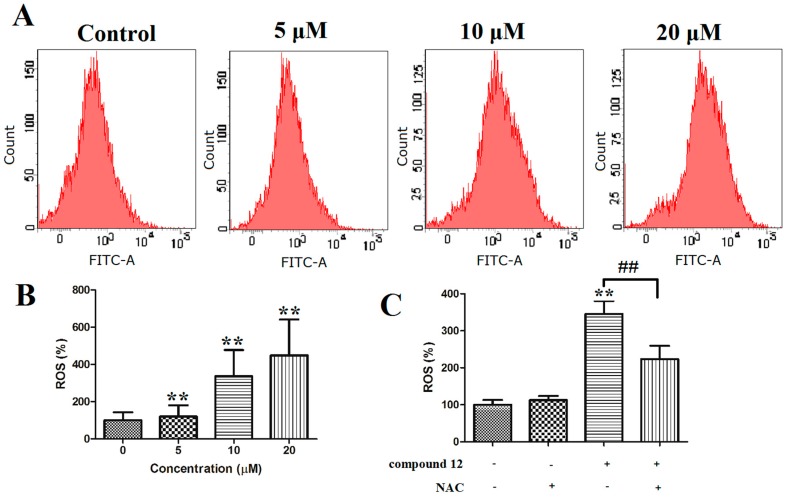
(**A**,**B**) PANC-1 cells were stained with 2′,7′-dichlorodihydrofluorescein in diacetate (DCFH-DA) and then analyzed using flow cytometry; (**C**) PANC-1 cells were treated with 10 µM compound **12** alone or in combination with NAC (5 mM) for 72 h. ** *p* < 0.01 vs. control group. ^##^ *p* < 0.01 vs. compound **12**(+)/NAC(−) group.

ROS (reactive oxygen species) plays an important role in cell proliferation or apoptosis [20,21], and it can induce cell death in a variety of ways. When intracellular ROS accumulates in cells, it causes the mitochondrial membrane potential damage and eventually leads to apoptosis [22,23]. To explore whether this compound triggers ROS generation, PANC-1 cells were stained with a fluorescent probe, 2′,7′-dichlorodihydrofluorescein in diacetate (DCFH-DA), which can detect intracellular ROS. The result showed that a rapid production of ROS could be detected in the PANC-1 cells after the treatment of compound **12**. As shown in Figure 7A,B, compared with that of the control, the ROS content in the experimental group increased to 120.09%, 336.99% and 449.09%. The ROS-mediated effects may be modulated by antioxidants such as *N*-acetylcysteine (NAC). Next, PANC-1 cells were treated with 10 µM compound **12** combined with/without 5 mM NAC (Beyotime, Nanjing, China), a ROS scavenger, for 72 h, cells were harvested and analyzed after staining with DCFH-DA. The results showed that compound **12**-induced ROS generation was blocked by NAC in PANC-1 cells (Figure 7C). These data indicated that compound **12** could induce ROS generation, and this might be a mechanism of apoptosis.

#### 2.3.6. Mechanism Study of Compound **12**

**Figure 8:**
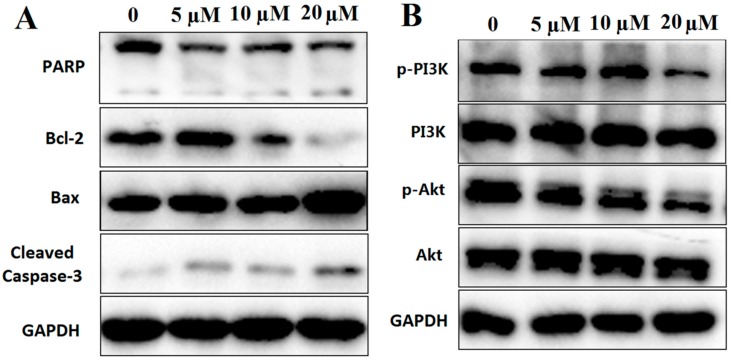
(**A**) Western blot analysis of apoptosis-related proteins, including PARP, Bcl-2, Bax and Cleaved-Caspase-3; (**B**) western blot analysis of PI3K/Akt pathway proteins, including PI3K and Akt. GAPDH was used to normalize protein content (*n* =3).

Apoptosis serves a key role in the regulation of cells. It mainly comprised two apoptotic pathways: The death receptor-mediated apoptosis pathway and the mitochondria-mediated apoptosis pathway [24]. In the mitochondria-mediated apoptosis pathway, proteins from the Bcl-2 family, such as Bax and Bcl-2, are the main components that regulate mitochondrial permeability [25]. In this study, it was demonstrated that compound **12** treatment could increase the ratio of Bax/Bcl-2 as well as activate Caspase-3 and PARP (Figure 8A).

PI3K/Akt signaling pathway plays an important role in the process of apoptosis especially in the ROS-mediated apoptotic pathway [26,27]. In this study, the phosphorylation of PI3K and Akt were decreased after treatment with compound **12** (Figure 8B). Based on the above studies, our results indicated that compound **12**-induced PANC-1 apoptosis may be through ROS-mediated PI3K/Akt signaling pathway.

## 3. Materials and Methods

### 3.1. General Experimental Procedures

The HRESIMS analyses were performed on a Waters Xevo G2-XS QTof mass spectrometer (Waters Corp., Milford Massachusetts, America). NMR spectra were recorded on an Ascend 600 MHz instrument (Bruker-Biospin, Billerica, MA, America). The analytical experiments were performed on a Shimadzu LC-20AT HPLC system (Shimadzu Corp., Kyoto, Japan) equipped with a Shimadzu InertSustain C18 column (4.6 I.D. × 250 mm, 5 μm, S/N 6LR98081). A Hanbon NP700 semipreparative HPLC (Hanbon Sci. & Tech., Jiangsu, China) equipped with a Shimadzu InertSustain C18 column (10 I.D. × 250 mm, 5 *μ*m, S/N 7ER43006) was used for purifying compounds. Biological assays were monitored on a BioTek ELx808 microplate spectrophotometer (BioTek Instruments, Inc., Winooski, America), a BD FACSCalibur flow cytometry (BD Biosciences, Franklin Lakes, America) and an Olympus BX-51 Fluorescence Microscopy (Olympus Corporation, Tokyo, Japan).

### 3.2. Fungal Material and Fermentation

The fungus strain *Aspergillus* sp. XNM-4 was isolated from *Leathesia nana*, which was collected in April 2017 in Weihai, Shandong Province, China (Latitude: 37°31′57.58′′N; Longitude: 122°02′52.85′′E). The strain was identified according to 18S rDNA gene sequence analysis by the Beijing Genomics Institute (Shenzhen, China).

The fungus *Aspergillus* sp. XNM-4 was fermented on Malt Extract Medium (130.0 g/L malt extract, 0.1 g/L chloramphenicol, 15.0 g/L agar, pH 5.6 ± 0.2) under static conditions at 25 °C for 10 days. A total of 300 culture dishes (90 * 15 mm) were used in the experiment.

### 3.3. Extraction and Isolation

The agar blocks with mycelium were collected in a 2 L beaker, and ultrasonically extracted with 1.5 L of ethyl acetate (three times and for 30 min each). The crude extract (10.3 g) was chromatographed on a silica gel column (4*40 cm, 200–300 mesh) and successively eluted with petroleum ether (0.5 L), petroleum-EtOAc (4:1, 2.5 L), petroleum-EtOAc (1:2, 2 L), EtOAc (1 L), and EtOAc-MeOH (1:2, 2 L). The eluents were concentrated by reduced pressure at 40 °C, and then merged in nine fractions under HPLC analysis, including Fractions A (985.5 mg), B (364.6 mg), C (41.7 mg), D (90.4 mg), E (220.2 mg), F (344.6 mg), G (384.7 mg), H (8.3 mg), and I (1611.4 mg). These subfractions were further purified by semipreparative HPLC using a continuous gradient of MeOH-H_2_O (60–100%, 20 min, 3 mL/min). The obtained eluents were extracted by ethyl acetate (v/v, 1:2) twice. After being dried by anhydrous Na_2_SO_4_, the organic phase was concentrated under a reduced pressure at 40 °C and then freeze-dried to yield compounds **1**–**18**. As a result, compounds **2** (3.1 mg,), **15** (5.2 mg), and **16** (4.4 mg) were from Fr. D, compounds **3** (3.0 mg), **4** (3.8 mg), **5** (9.2 mg), **6** (7.0 mg), **7** (5.3 mg), **9** (13.4 mg), **11** (4.2 mg), **13** (8.9 mg), and **14** (11.6 mg) were from Fr. E, compounds **8** (7.4 mg), **10** (8.2 mg), **12** (8.8 mg), **17** (29.2 mg), **18** (5.4 mg) were from Fr. F, compound **1** (5.0 mg) was from Fr. G. The purities of all isolated compounds was determined to be >95% under two solvent conditions by analytical HPLC recorded on a Shimadzu LC-20A system. Solvent conditions A: CH_3_OH/H_2_O with 0.1% trifluoroacetic acid 60–100% (20 min); Solvent conditions B: CH_3_CN/H_2_O with 0.1% trifluoroacetic acid 30–100% (20 min); UV detection, 254 nm; flow rate, 1.0 mL/min; temperature, 40 °C; injection volume, 30 μL. The analytical HPLC spectra were listed on page S37–S54 in the Supplementary Materials.

*(7R)-(hydroxy(phenyl)methyl)-4H-pyran-4-one (****1****)*: white amorphous powder; ${\left[\alpha \right]}_{D}^{20}$ +78.1° (*c* 0.10, MeOH); UV (MeOH) *λ*_max_ (log *ε*) 248 (4.14) nm; ECD (MeOH) *λ*_max_ (Δ*ε*) 201 (+12.65), 228 (−6.13), 250 (+3.01) nm; IR (KBr) *ν*_max_ 3447, 1653, 1602, 1384 cm^−1^; ^1^H; for ^13^C NMR data see Table 1; HRESIMS (*m*/*z*): 203.0707 [M + H]^+^ (calcd for C_12_H_11_O_3_, 203.0708).

### 3.4. Biological Activity Test

#### 3.4.1. Cell Culture

PANC-1, A549, MDA-MB-231, Caco-2, SK-OV-3 and HL-7702 were supplied by Cell Bank, Chinese Academy of Sciences (Shanghai, China). These cells were separately maintained in DMEM medium, F-12K medium, L15 medium, MEM medium, McCoy′s 5A (Modified) medium, and RPMI-1640 medium. All media were supplemented with 10% FBS, 100 U/mL penicillin and 100 µg/mL streptomycin. Cells were cultured at 37 °C in a humidified CO_2_ (5%).

#### 3.4.2. Determination of Cell Viability

Cell viability was evaluated by a 3-(4,5-dimethyl-2-thiazolyl)-2,5-diphenyl tetrazolium bromide (MTT) assay [28]. For the preliminary anti-tumor activity screening, the cells were plated in 96-well plates (3 × 10^3^ cells/well for A549 and PANC-1, A549, MDA-MB-231 and Caco-2, 5 × 10^3^ cells/well for SK-OV-3 and GL-7702) and incubated with the tested compounds at a concentration of 50 µM for 72 h. For detection of the IC_50_, cells were treated with varying concentrations of **12** (0, 6.25, 12.5, 25, 50 µM) for 72 h. After incubation, MTT (5 mg/mL) was added and incubated at 37 °C for 4 h. The formazan was dissolved by DMSO and measured using a microplate reader at 490 nm.

#### 3.4.3. Colony Forming Assay

The PANC-1 cells were seeded in 6-well plates (1000 cells/well) and treated with varying concentrations of **12** (0, 5, 10, 20 µM). These cells were further incubated for 10 days to allow colony formation; then, the cells were fixed with 4% paraformaldehyde for 10 min. After three washes, the cells were finally stained with crystal violet for 10 min. Cells > 50 were scored as colonies [29].

#### 3.4.4. Analysis of Apoptosis

The PANC-1 cells were seeded in 6-well plates (2 × 10^5^/well). After 24 h of incubation, the cells were treated with compound **12** (0, 5, 10, 20 µM) for 72 h. After being harvested and washed with PBS, PANC-1 cells were stained with Annexin V/PI for 15 min. Finally, the cells were detected and analyzed by flow cytometry.

#### 3.4.5. Hoechst 33258 Staining

The PANC-1 cells were seeded in 6-well plates (2 × 10^5^/well). After 24 h of incubation, the cells were treated with compound **12** (0, 5, 10, 20 µM) for 72 h. Next, the cells were stained with Hoechst dye 33258 for five min at room temperature and assessed by a fluorescence microscopy.

#### 3.4.6. Analysis of Cell Cycle

The PANC-1 cells were seeded in 6-well plates (2 × 10^5^/well). After 24 h of incubation, the cells were treated with varying concentrations of compound **12** (0, 5, 10, 20 µM) for 72 h. After fixed in cold 75% ethanol at −20 °C overnight, the cells were washed twice with PBS and stained with a PI solution containing 20 μg/mL of RNaseA and 50 μg/mL of PI for 30 min. Finally, the cells were detected and analyzed by flow cytometry.

#### 3.4.7. Measurement of Intracellular ROS

The PANC-1 cells were seeded in 6-well plates (2 × 10^5^/well). After 24 h of incubation, the cells were treated with varying concentrations of compound **12** (0, 5, 10, 20 µM) for 72 h. After being stained with 10 μM of DCFH-DA at 37 °C for 30 min, the cells were washed with media and were detected and analyzed by flow cytometry.

#### 3.4.8. Western Blot Analysis

PANC-1 cells were harvested and seeded in 6-cell plates and allowed to settle overnight. Cells were treated with compound **12** (0, 5, 10 and 20 µM) for 72 h. Proteins were harvested and separated by SDS-PAGE and transferred onto PVDF membranes. Membranes were blocked in a blocking solution (containing 5% non-fat milk) and subsequently probed with primary antibodies at 4 °C overnight. After 15 min washes in TBST, the membranes were incubated with a secondary antibody for 1 h at room temperature. Antibodies against Bcl-2, Bax, PARP, Cleaved-Caspase-3, phosphorylation-Akt, Akt and GAPDH were purchased from Cell Signaling Technology (Beverly, MA, USA). Phosphorylation-PI3K and PI3K were purchased from Abcam (Cambridge, UK). The anti-mouse IgG and anti-rabbit secondary antibodies raised from goat were obtained from Abcam (Cambridge, UK). The bands were detected using an enhanced chemiluminescence system BeyoECL Plus (Beyotime, Nanjing, China).

## 4. Conclusions

In the present study, eighteen metabolites, including one new pyrone derivative (**1**), were identified from the culture of an algae-derived endophytic fungus *Aspergillus* sp. XNM-4. Among them, compounds **1**, **17**, and **18** were first reported from the genus *Aspergillus*. The pharmacological experiments showed that dimeric naphthopyrones **7**, **10**, and especially **12**, possessed potent cytotoxicity on five human cancer cell lines (PANC-1, A549, MDA-MB-231, Caco-2, and SK-OV-3), and one human normal cell line (HL-7702). Further studies indicated that compound **12** induced apoptosis, arrested the cell cycle at the G0/G1 phase in PANC-1 cells, caused morphological changes and generated ROS. Mechanism studies found that compound **12** induced PANC-1 apoptosis was via ROS-mediated PI3K/Akt signaling pathway. These experimental results may be beneficial for the development of naturally occurring dimeric naphthopyrones as anti-tumor agents.

## Figures and Tables

**Table 1:** ^1^H, ^13^C NMR and HMBC spectroscopic data of **1** (600 MHz, ppm in DMSO-*d*_6_).

Position	*δ*_H_ (*J* in Hz)	*δ*_C_ (m)	Key HMBC (H→C)
2		170.2	
3	6.42, d (*J* = 2.4)	112.3	C-5, 7
4		177.9	
5	6.21, dd (*J* = 2.4, 6.0)	116.2	C-3
6	8.05, d (*J* = 6.0)	156.2	C-4
7	5.48, s	71.2	C-3, 9, 13
8		140.6	
9	7.37, overlap	126.8	C-7, 11
10	7.41, overlap	128.4	C-8
11	7.32, m	128.0	C-9, 13
12	7.41, overlap	128.4	C-8
13	7.37, overlap	126.8	C-7, 11

## Supplementary Materials

The following are available online at https://www.mdpi.com/1660-3397/17/4/207/s1, including the HPLC, UV, IR, HRESIMS, 1D NMR, HSQC, and HMBC spectra for compound **1**, the HPLC, HRESIMS and 1D NMR spectra for compounds **2**–**18**, and the ECD calculation for compounds **1** and **12**.
