# Supplementary material for: Discovery of Natural Dimeric Naphthopyrones as Potential Cytotoxic Agents through ROS-Mediated Apoptotic Pathway
Source: Mar Drugs. 2019 Apr 2;17(4):207. doi: 10.3390/md17040207 (PMC6520789; doi:10.3390/md17040207)
Supplement: Supplementary file 1 [file marinedrugs-17-00207-s001.pdf]

Supplementary Materials

Discovery of Natural Dimeric Naphthopyrones as Potential Cytotoxic Agents Through ROS-mediated Apoptotic Pathway

Kuo Xu [1,†], Chuanlong Guo [1,2,†], Jie Meng [1,4], Haiying Tian [5], Shuju Guo [1,*] and Dayong Shi [1,3,*]

[1] Chinese Academy of Sciences Key Laboratory of Experimental Marine Biology, Institute of Oceanology, Chinese Academy of Sciences, Qingdao 266071, China; xukuoworld@126.com (K.X.); gcl_cpu@126.com (C.G.); mengjie@qibebt.ac.cn (J.M.)

[2] Department of Pharmacy, College of Chemical Engineering, Qingdao University of Science and Technology, Qingdao 266042, China

[3] State Key Laboratory of Microbial Technology, School of Life Science, Shandong University, No. 72 Binhai Road, Qingdao 266237, China

[4] College of Resources and Environment, Qingdao Agricultural University, Qingdao 266109, China;

[5] Technology Center, China Tobacco Henan Industrial Co., Ltd., Zhengzhou, 450000, China; 13623810925@126.com

* Correspondence: guoshuju@qdio.ac.cn (S.G.); shidayong@qdio.ac.cn (D.S.); Tel.: +86-532-82898741 (S.G.); +86-532-82898719 (D.S.)

† These authors contributed equally to this work.

Table of Contents

NO.	Contents	Page
**Figure S1**	The UV spectrum of **1** in MeOH	S4
**Figure S2**	The ECD spectrum of **1** in MeOH	S4
**Figure S3**	The HR-ESI-MS data of **1**	S5
**Figure S4**	The IR spectrum (KBr) of **1**	S5
**Figure S5**	The ^{1}H NMR spectrum (600 MHz) of **1** in DMSO-d_6	S6
**Figure S6**	The ^{13}C NMR spectrum (150 MHz) of **1** in DMSO-d_6	S6
**Figure S7**	The HSQC spectrum (600 MHz) of **1** in DMSO-d_6	S7
**Figure S8**	The HMBC spectrum (600 MHz) of **1** in DMSO-d_6	S7
**Figure S9**	The HR-ESI-MS data of **2**	S8
**Figure S10**	The ^{1}H NMR spectrum (600 MHz) of **2** in DMSO-d_6	S8
**Figure S11**	The ^{13}C NMR spectrum (150 MHz) of **2** in DMSO-d_6	S9
**Figure S12**	The HR-ESI-MS data of **3**	S9
**Figure S13**	The ^{1}H NMR spectrum (600 MHz) of **3** in acetone-d_6	S10
**Figure S14**	The ^{13}C NMR spectrum (150 MHz) of **3** in acetone-d_6	S10
**Figure S15**	The HR-ESI-MS data of **4**	S11
**Figure S16**	The ^{1}H NMR spectrum (600 MHz) of **4** in acetone-d_6	S11
**Figure S17**	The ^{13}C NMR spectrum (150 MHz) of **4** in acetone-d_6	S12

**Figure S18**	The HR-ESI-MS data of **5**	S12
**Figure S19**	The ^{1}H NMR spectrum (600 MHz) of **5** in $CDCl_3$	S13
**Figure S20**	The ^{13}C NMR spectrum (150 MHz) of **5** in $CDCl_3$	S13
**Figure S21**	The HR-ESI-MS data of **6**	S14
**Figure S22**	The ^{1}H NMR spectrum (600 MHz) of **6** in acetone-d_6	S14
**Figure S23**	The ^{13}C NMR spectrum (150 MHz) of **6** in acetone-d_6	S15
**Figure S24**	The HR-ESI-MS data of **7**	S15
**Figure S25**	The ^{1}H NMR spectrum (600 MHz) of **7** in acetone-d_6	S16
**Figure S26**	The ^{13}C NMR spectrum (150 MHz) of **7** in acetone-d_6	S16
**Figure S27**	The HR-ESI-MS data of **8**	S17
**Figure S28**	The ^{1}H NMR spectrum (600 MHz) of **8** in acetone-d_6	S17
**Figure S29**	The ^{13}C NMR spectrum (150 MHz) of **8** in acetone-d_6	S18
**Figure S30**	The HR-ESI-MS data of **9**	S18
**Figure S31**	The ^{1}H NMR spectrum (600 MHz) of **9** in acetone-d_6	S19
**Figure S32**	The ^{13}C NMR spectrum (150 MHz) of **9** in acetone-d_6	S19
**Figure S33**	The HR-ESI-MS data of **10**	S20
**Figure S34**	The ^{1}H NMR spectrum (600 MHz) of **10** in acetone-d_6	S20
**Figure S35**	The ^{13}C NMR spectrum (150 MHz) of **10** in acetone-d_6	S21
**Figure S36**	The HR-ESI-MS data of **11**	S21
**Figure S37**	The ^{1}H NMR spectrum (600 MHz) of **11** in acetone-d_6	S22
**Figure S38**	The ^{13}C NMR spectrum (150 MHz) of **11** in acetone-d_6	S22
**Figure S39**	The HR-ESI-MS data of **12**	S23
**Figure S40**	The ECD spectrum of **12** in MeOH	S23
**Figure S41**	The ^{1}H NMR spectrum (600 MHz) of **12** in acetone-d_6	S24
**Figure S42**	The ^{13}C NMR spectrum (150 MHz) of **12** in acetone-d_6	S24
**Figure S43**	The HR-ESI-MS data of **13**	S25
**Figure S44**	The ^{1}H NMR spectrum (600 MHz) of **13** in acetone-d_6	S25
**Figure S45**	The ^{13}C NMR spectrum (150 MHz) of **13** in acetone-d_6	S26
**Figure S46**	The HR-ESI-MS data of **14**	S26
**Figure S47**	The ^{1}H NMR spectrum (600 MHz) of **14** in DMSO-d_6	S27
**Figure S48**	The ^{13}C NMR spectrum (150 MHz) of **14** in DMSO-d_6	S27
**Figure S49**	The HR-ESI-MS data of **15**	S28
**Figure S50**	The ^{1}H NMR spectrum (600 MHz) of **15** in acetone-d_6	S28
**Figure S51**	The ^{13}C NMR spectrum (150 MHz) of **15** in acetone-d_6	S29
**Figure S52**	The HR-ESI-MS data of **16**	S29

**Figure S53**	The ^{1}H NMR spectrum (600 MHz) of **16** in acetone-d_6	S30
**Figure S54**	The ^{13}C NMR spectrum (150 MHz) of **16** in acetone-d_6	S30
**Figure S55**	The HR-ESI-MS data of **17**	S31
**Figure S56**	The ^{1}H NMR spectrum (600 MHz) of **17** in acetone-d_6	S31
**Figure S57**	The ^{13}C NMR spectrum (150 MHz) of **17** in acetone-d_6	S32
**Figure S58**	The HR-ESI-MS data of **18**	S32
**Figure S59**	The ^{1}H NMR spectrum (600 MHz) of **18** in acetone-d_6	S33
**Figure S60**	The ^{13}C NMR spectrum (150 MHz) of **18** in acetone-d_6	S33
—	General procedure for ECD calculations of compounds **1** and **12**	S34−S36
—	Analytical HPLC spectra for all isolated compounds (**1**−**18**)	S37−S54

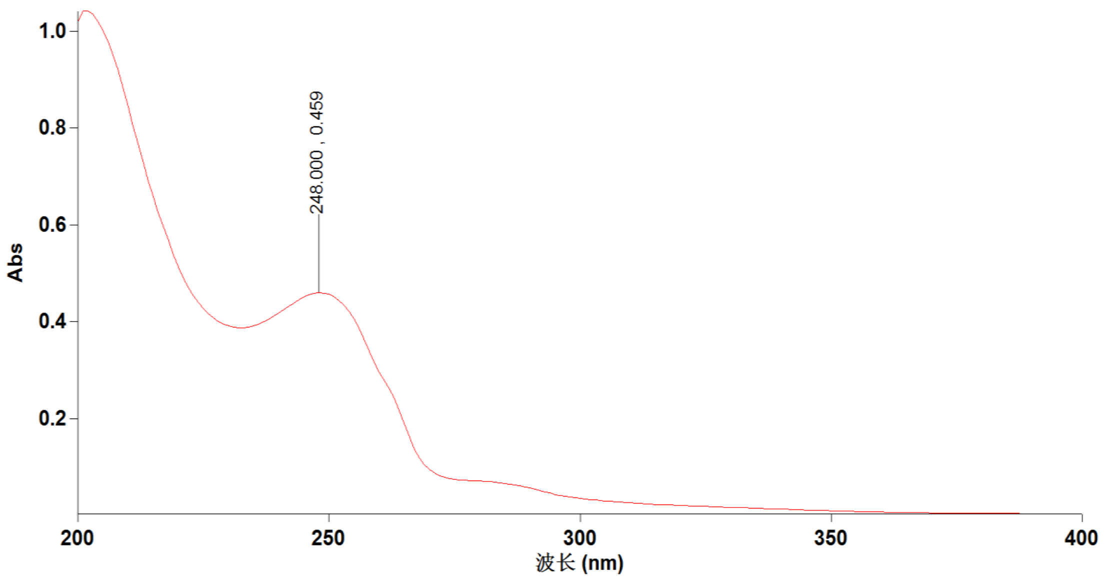

Figure S1. The UV spectrum of **1** in MeOH

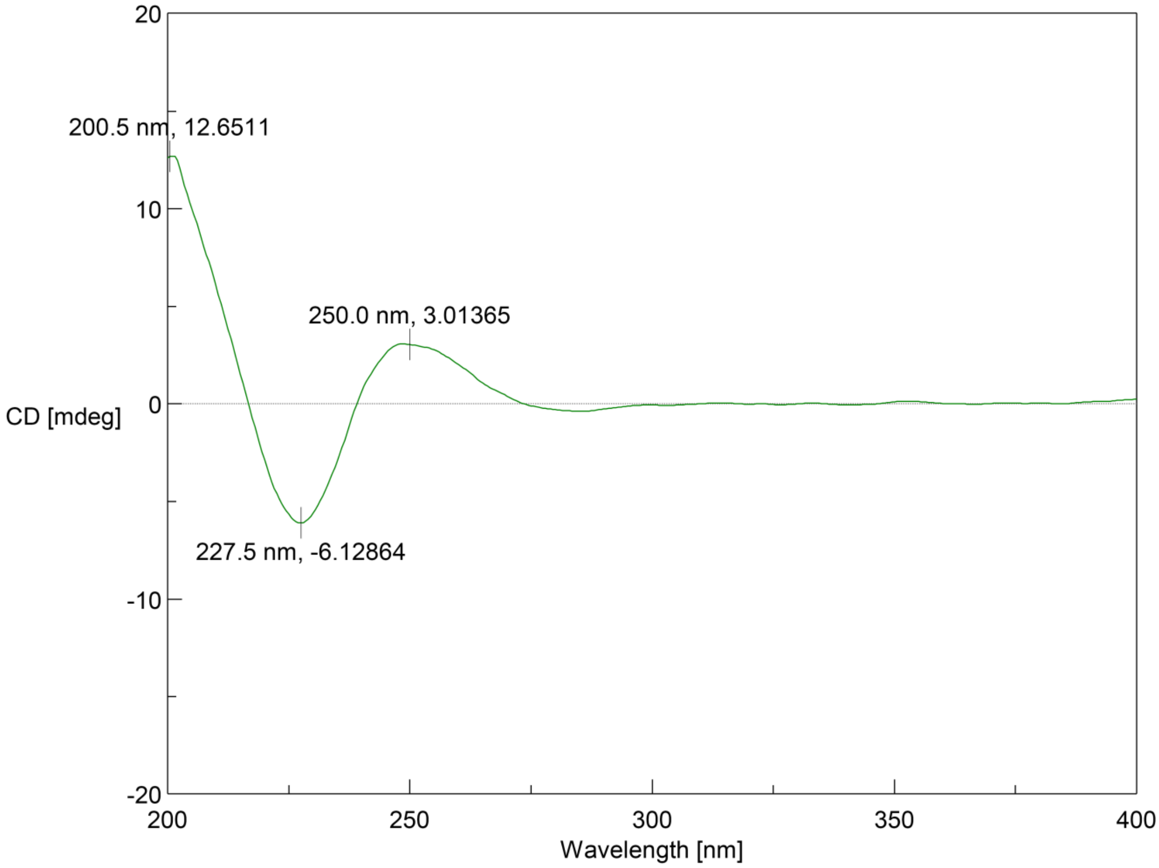

Figure S2. The ECD spectrum of **1** in MeOH

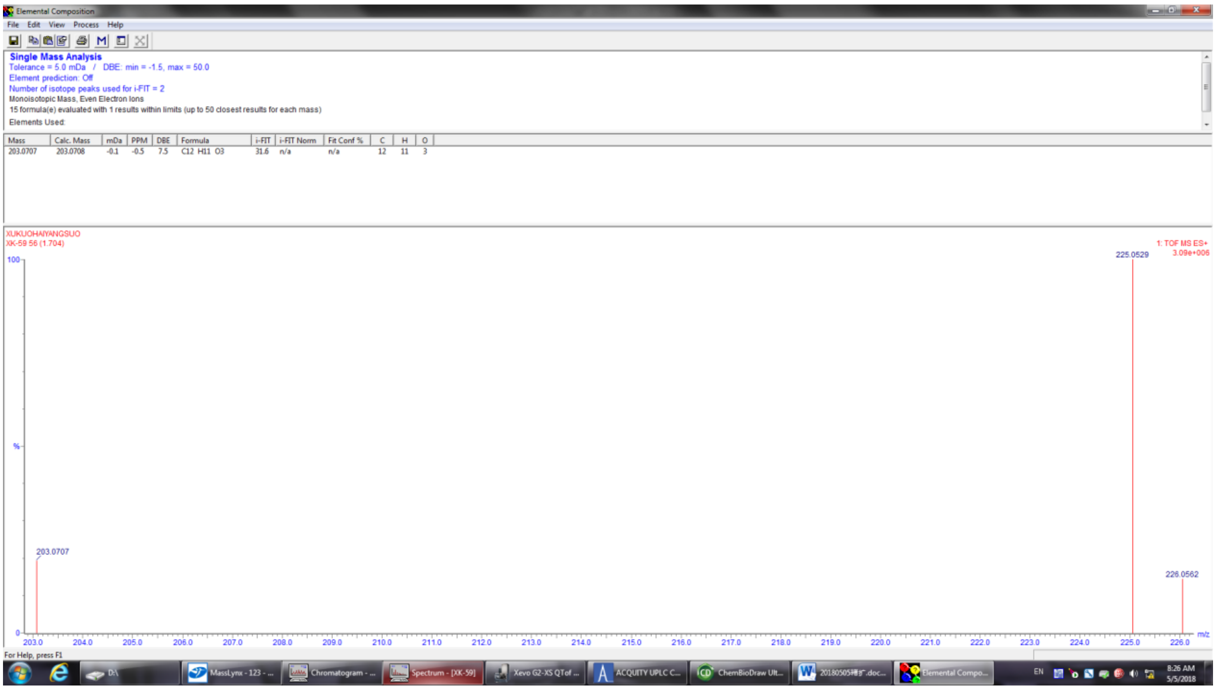

Figure S3. The HR-ESI-MS data of **1**

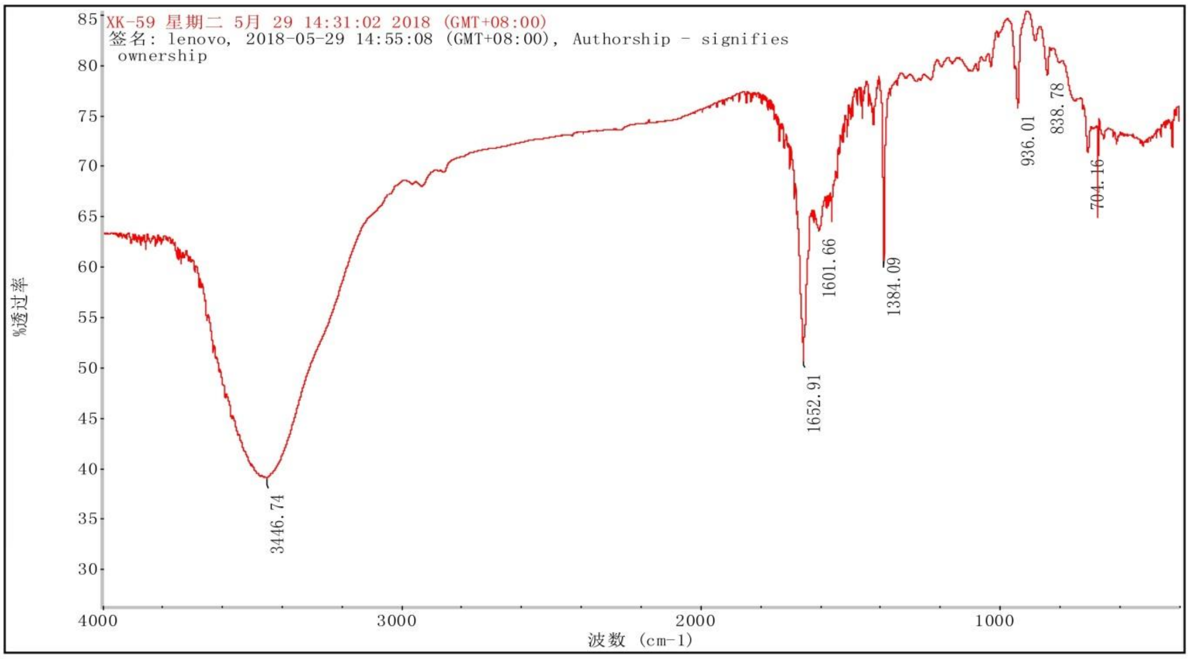

Figure S4. The IR spectrum (KBr) of **1**

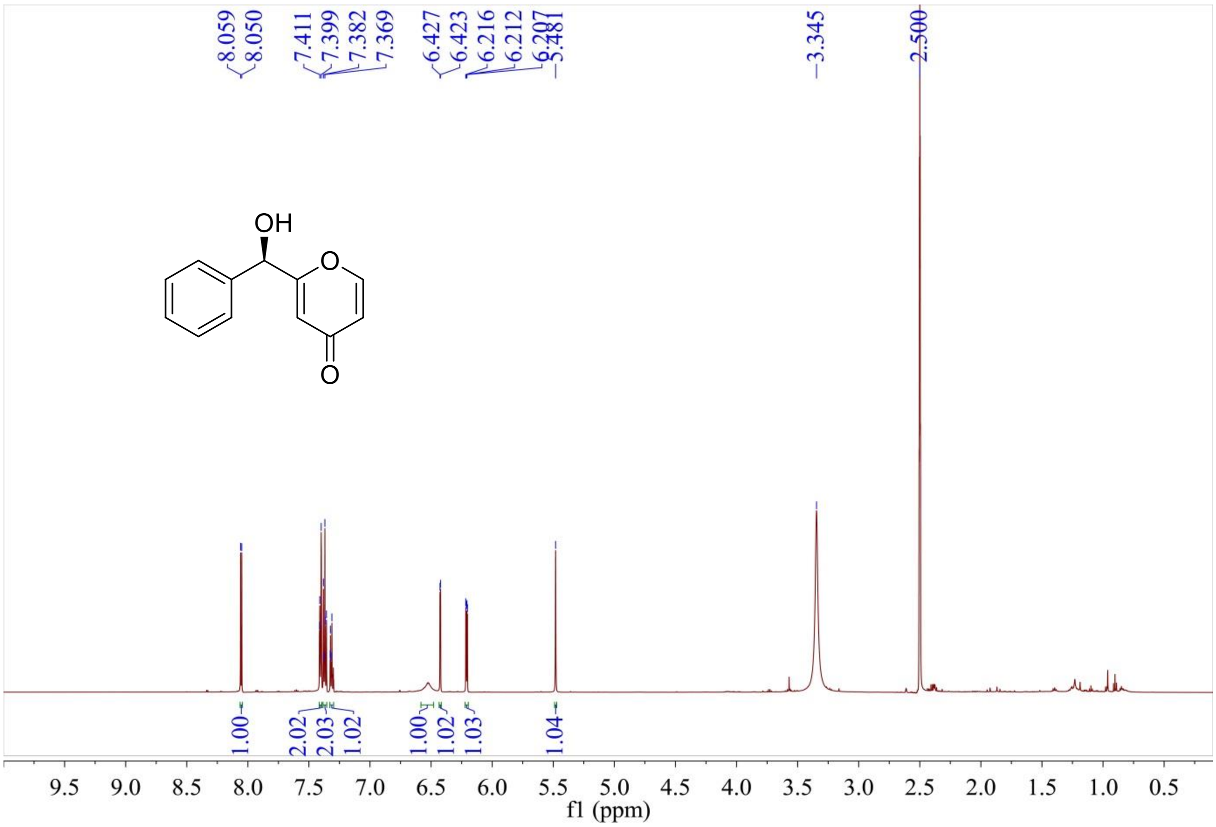

Figure S5. The ^{1}H NMR spectrum (600 MHz) of **1** in DMSO-d_6

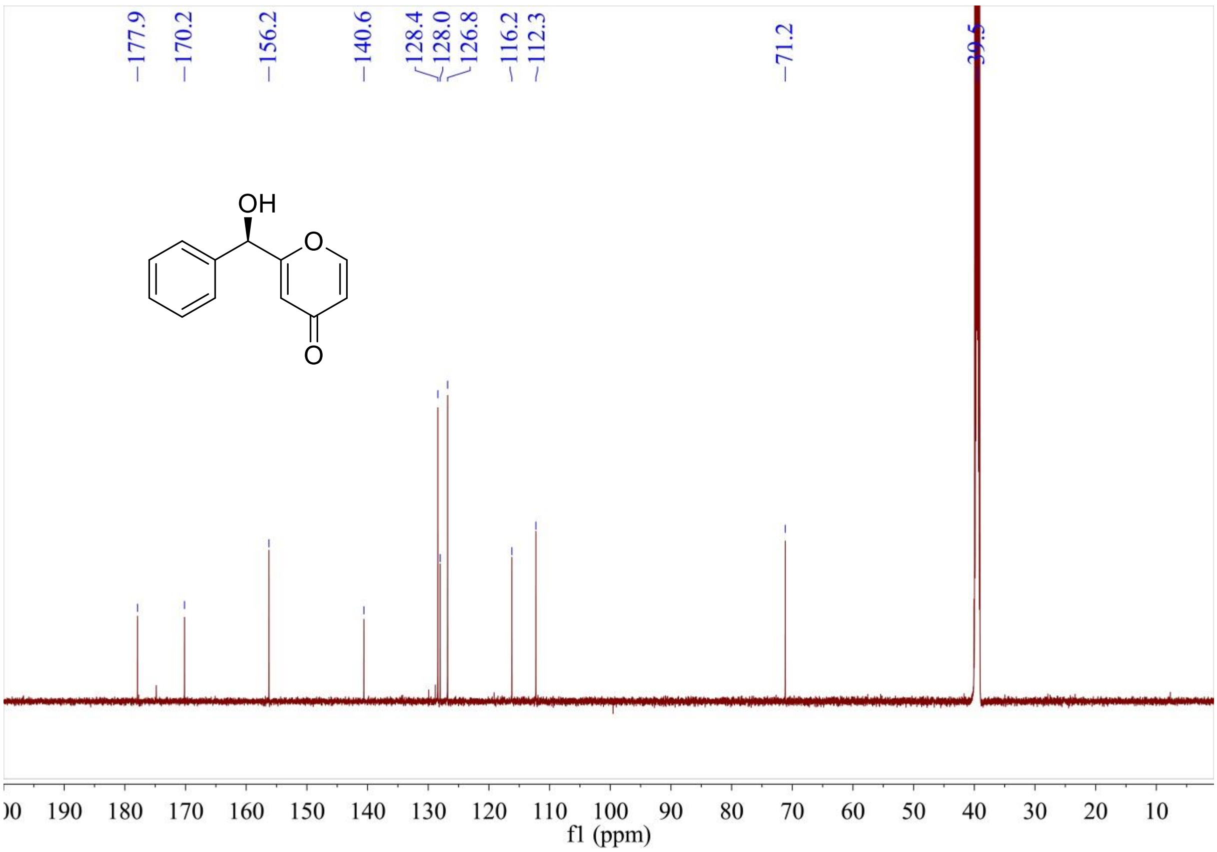

Figure S6. The ^{13}C NMR spectrum (150 MHz) of **1** in DMSO-d_6

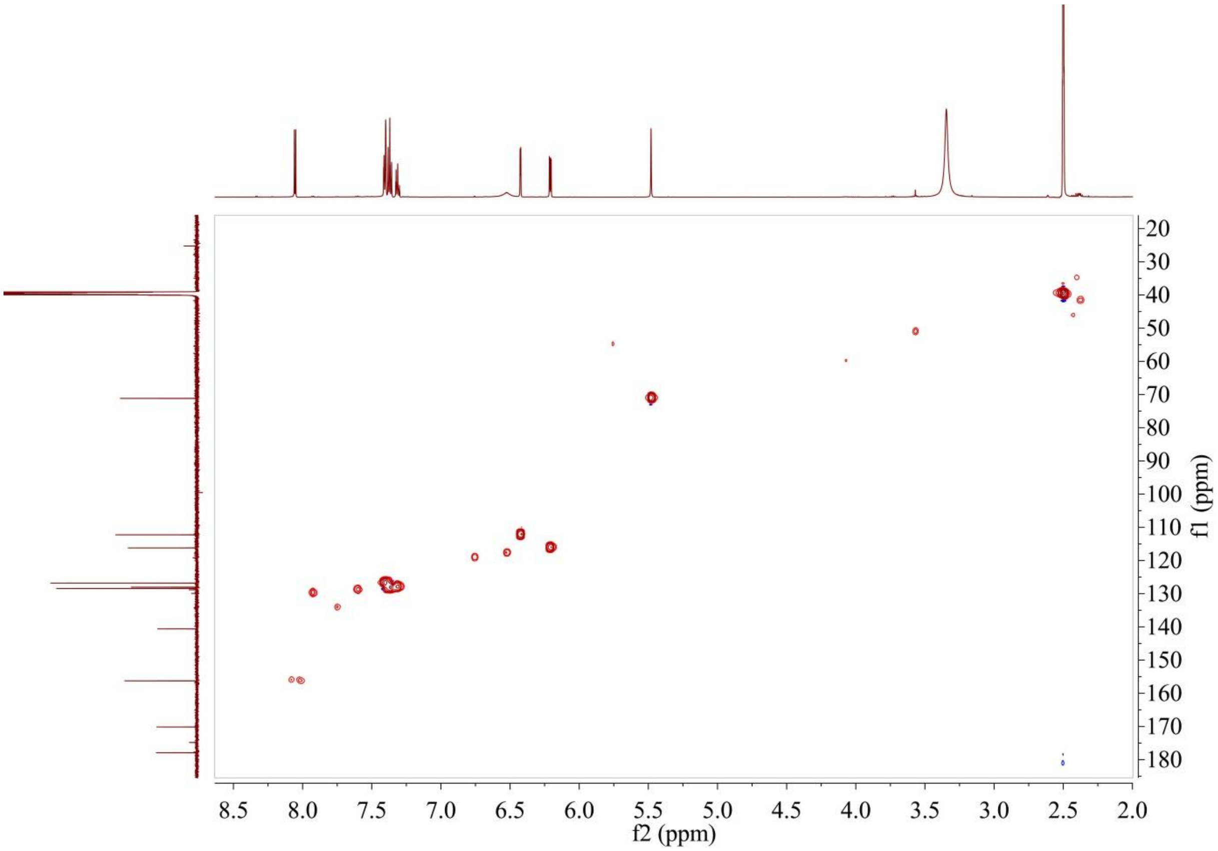

Figure S7. The HSQC spectrum (600 MHz) of **1** in DMSO-d_6

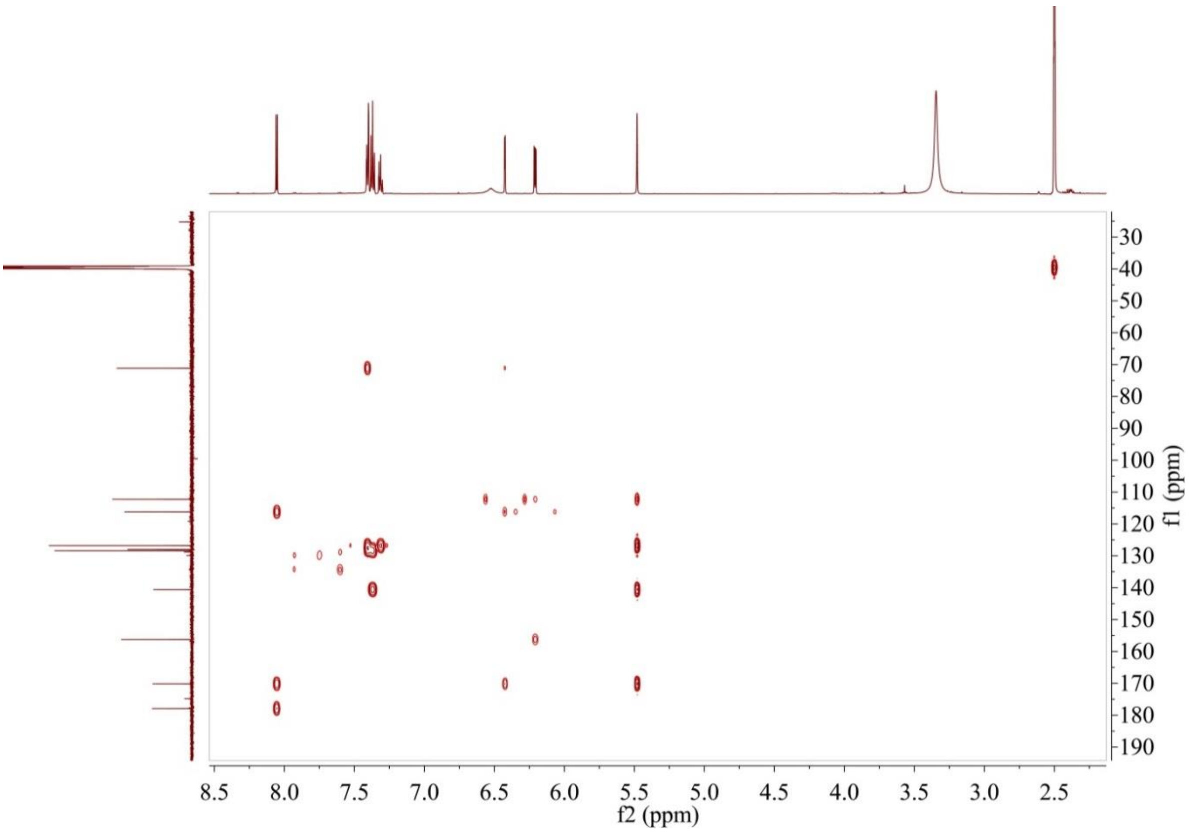

Figure S8. The HMBC spectrum (600 MHz) of **1** in DMSO-d_6

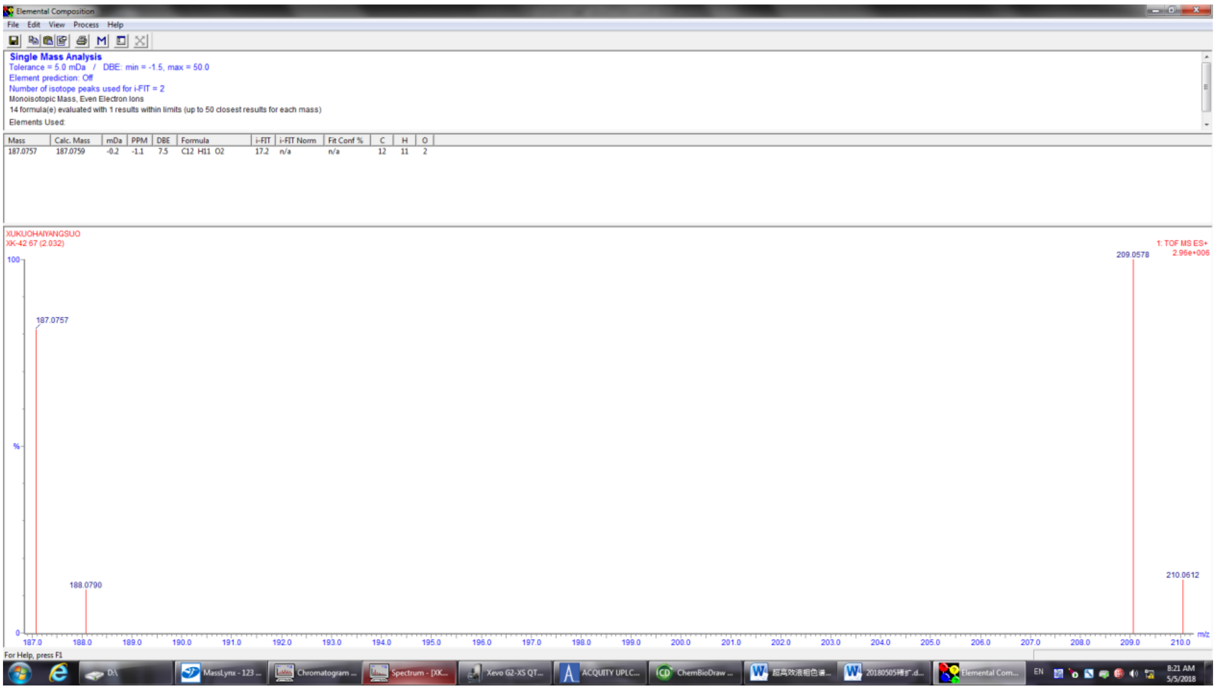

Figure S9. The HR-ESI-MS data of **2**

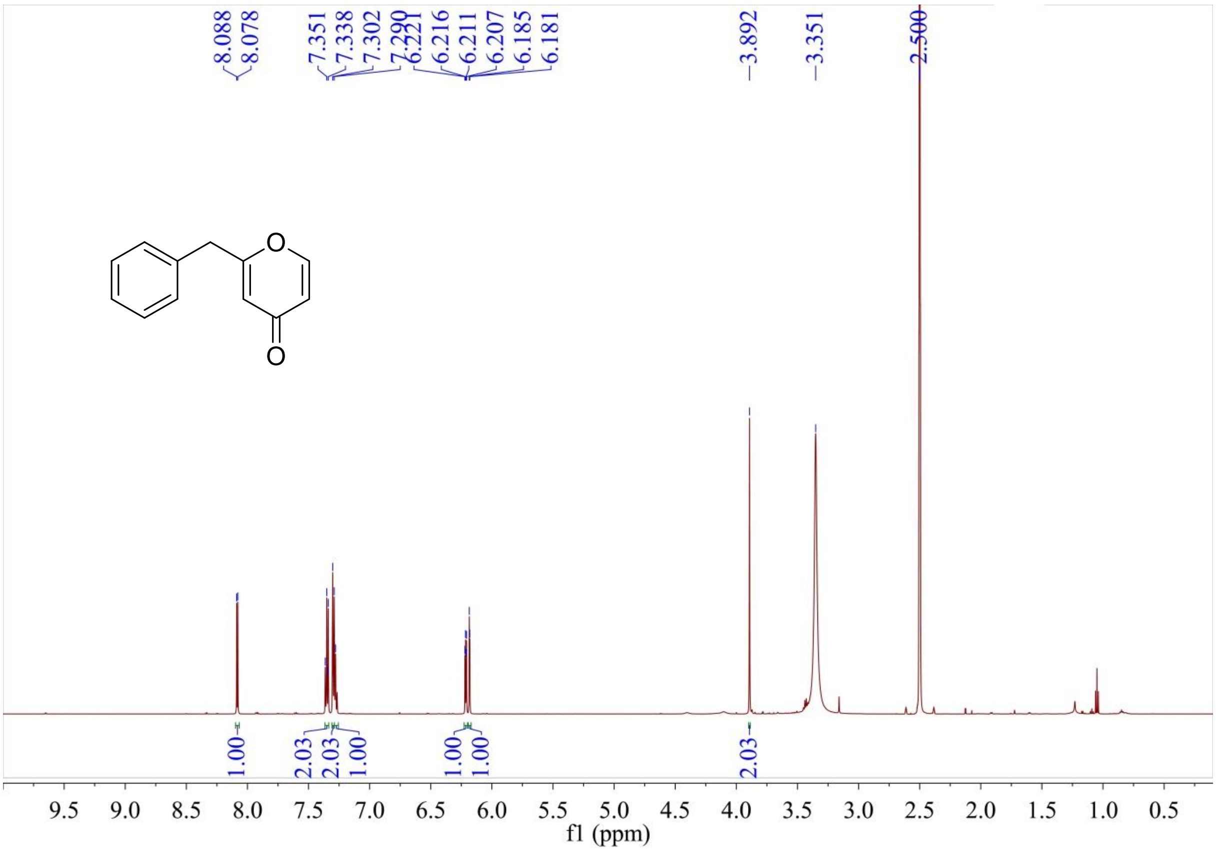

Figure S10. The ^{1}H NMR spectrum (600 MHz) of **2** in DMSO-d_6

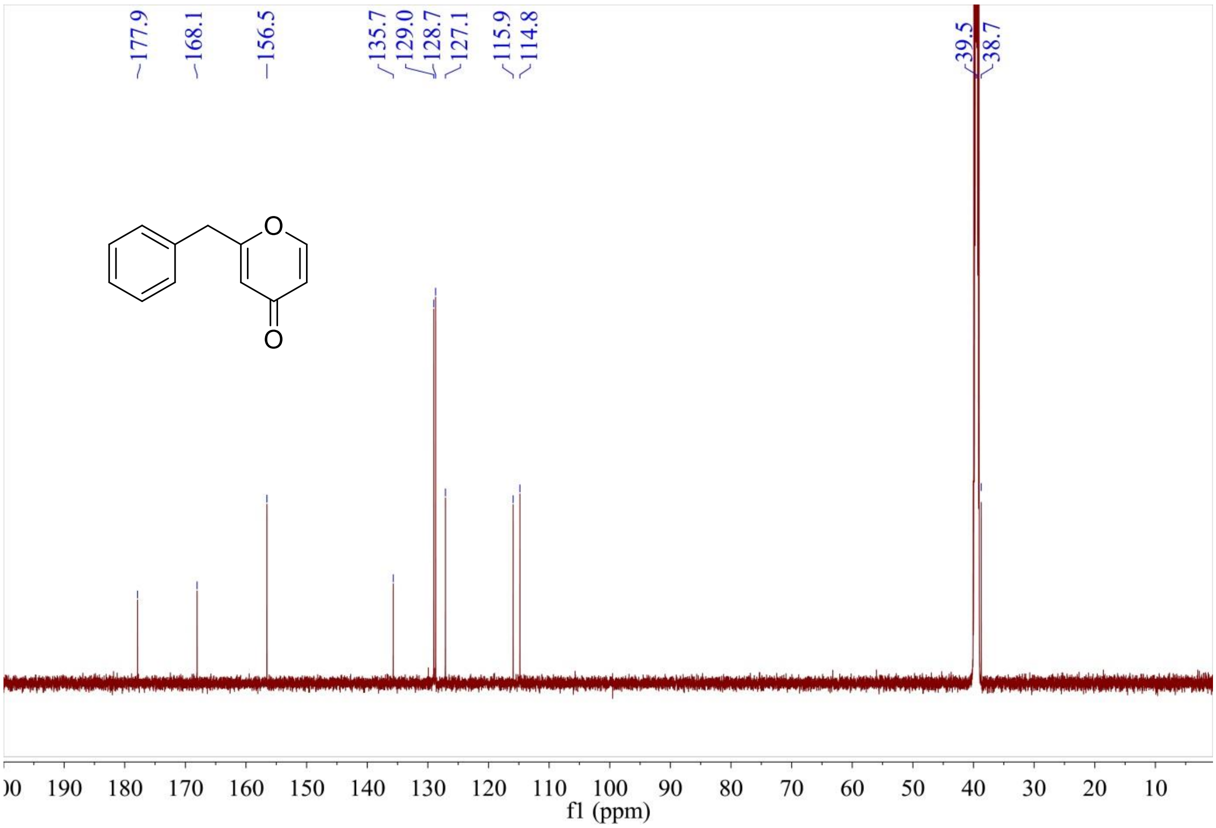

Figure S11. The ^{13}C NMR spectrum (150 MHz) of **2** in DMSO-d_6

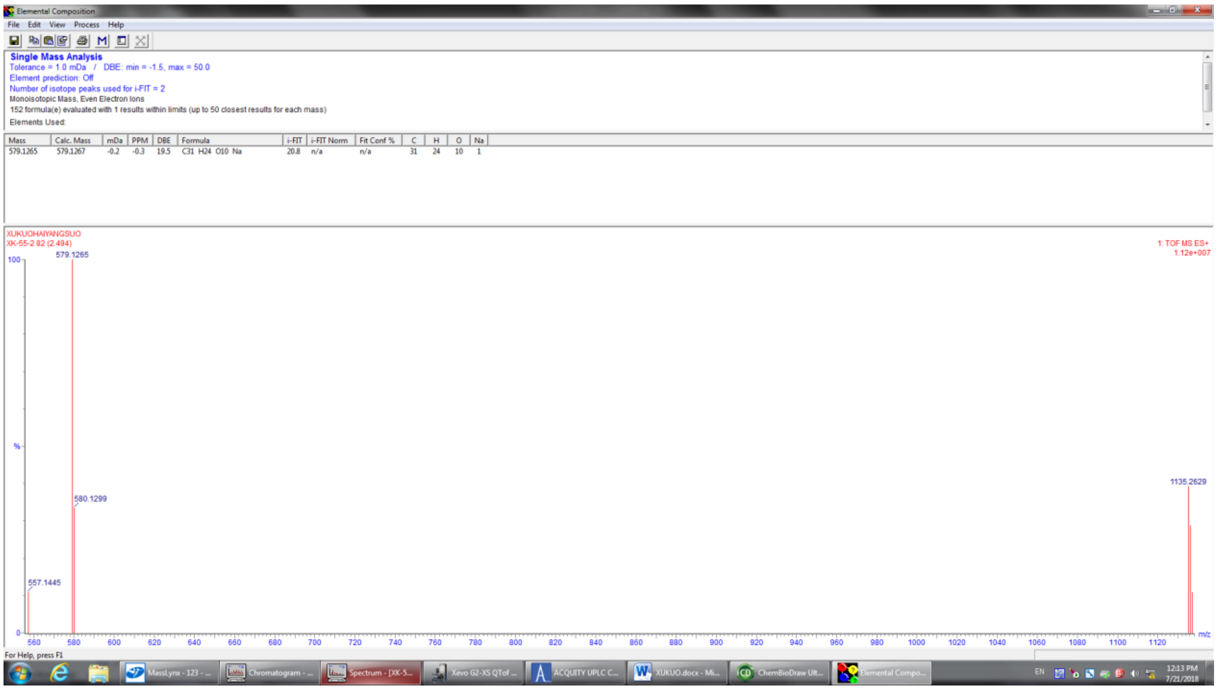

Figure S12. The HR-ESI-MS data of **3**

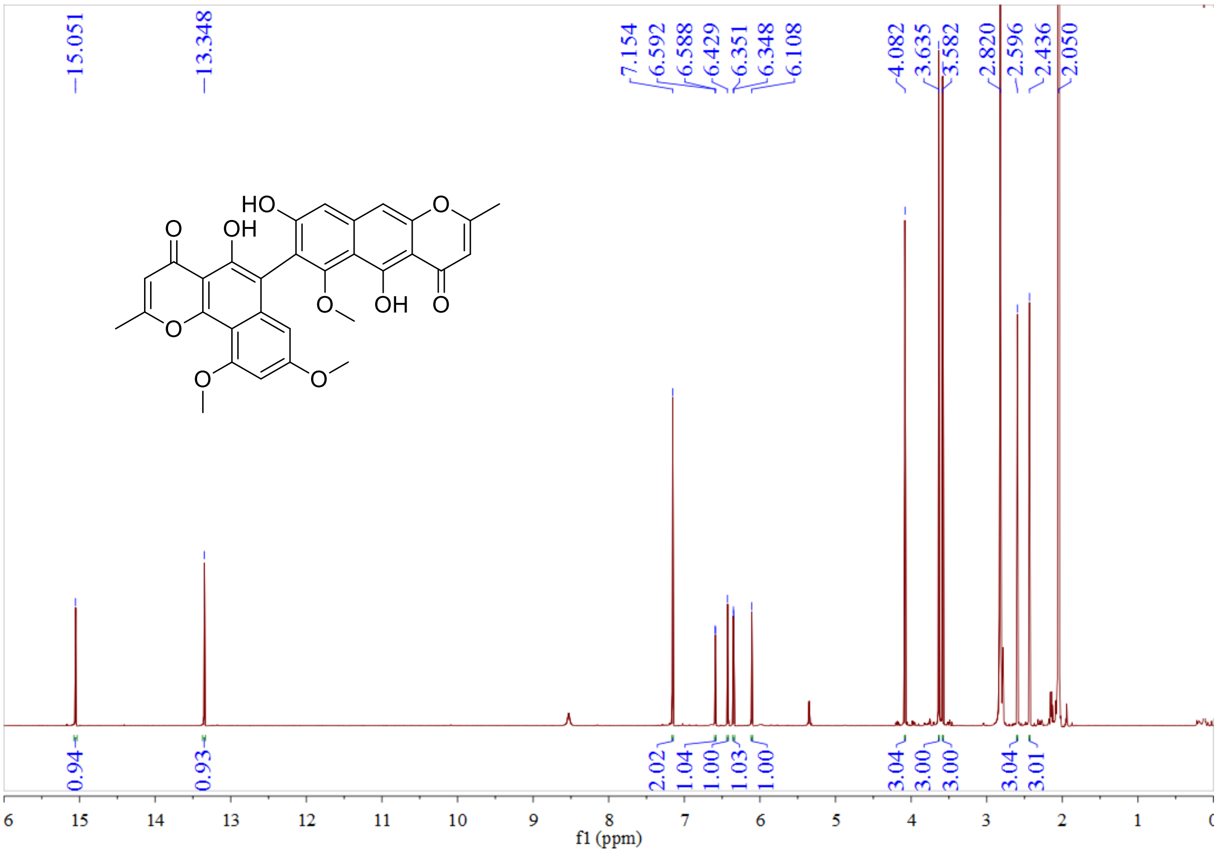

Figure S13. The ^{1}H NMR spectrum (600 MHz) of **3** in acetone-d_6

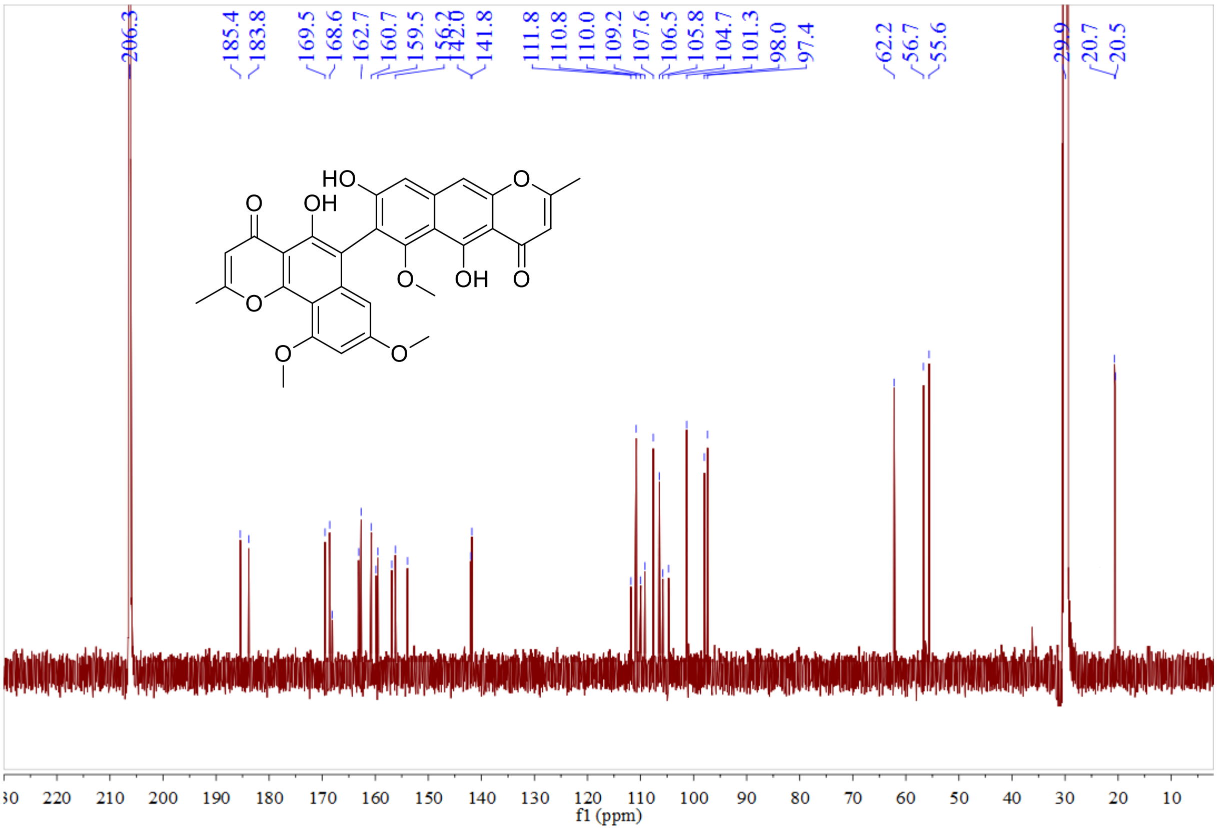

Figure S14. The ^{13}C NMR spectrum (150 MHz) of **3** in acetone-d_6

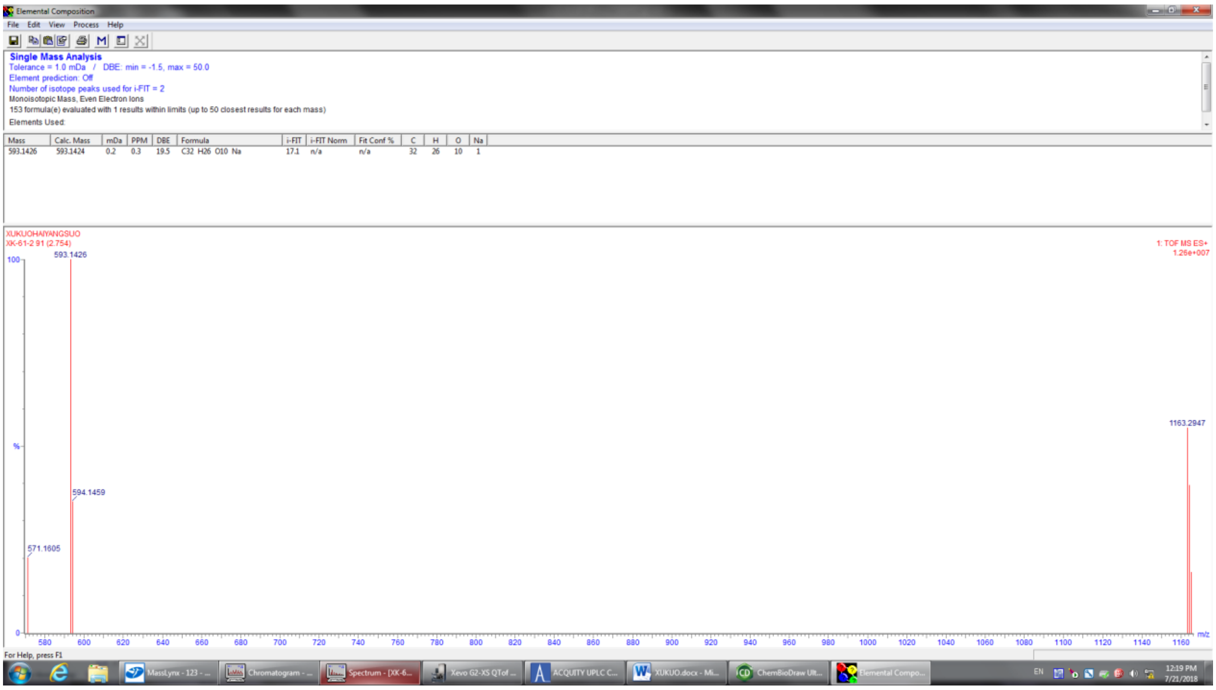

Figure S15. The HR-ESI-MS data of **4**

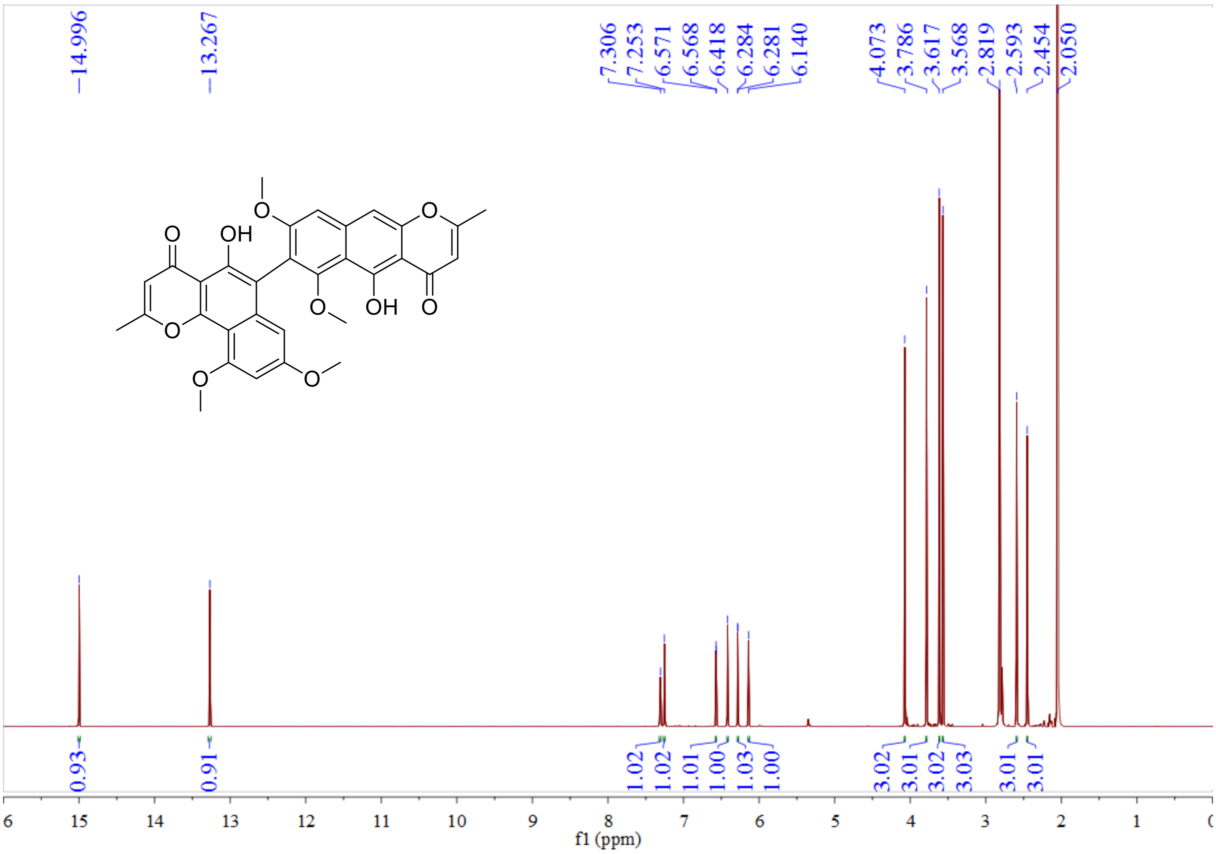

Figure S16. The ^{1}H NMR spectrum (600 MHz) of **4** in acetone-d_6

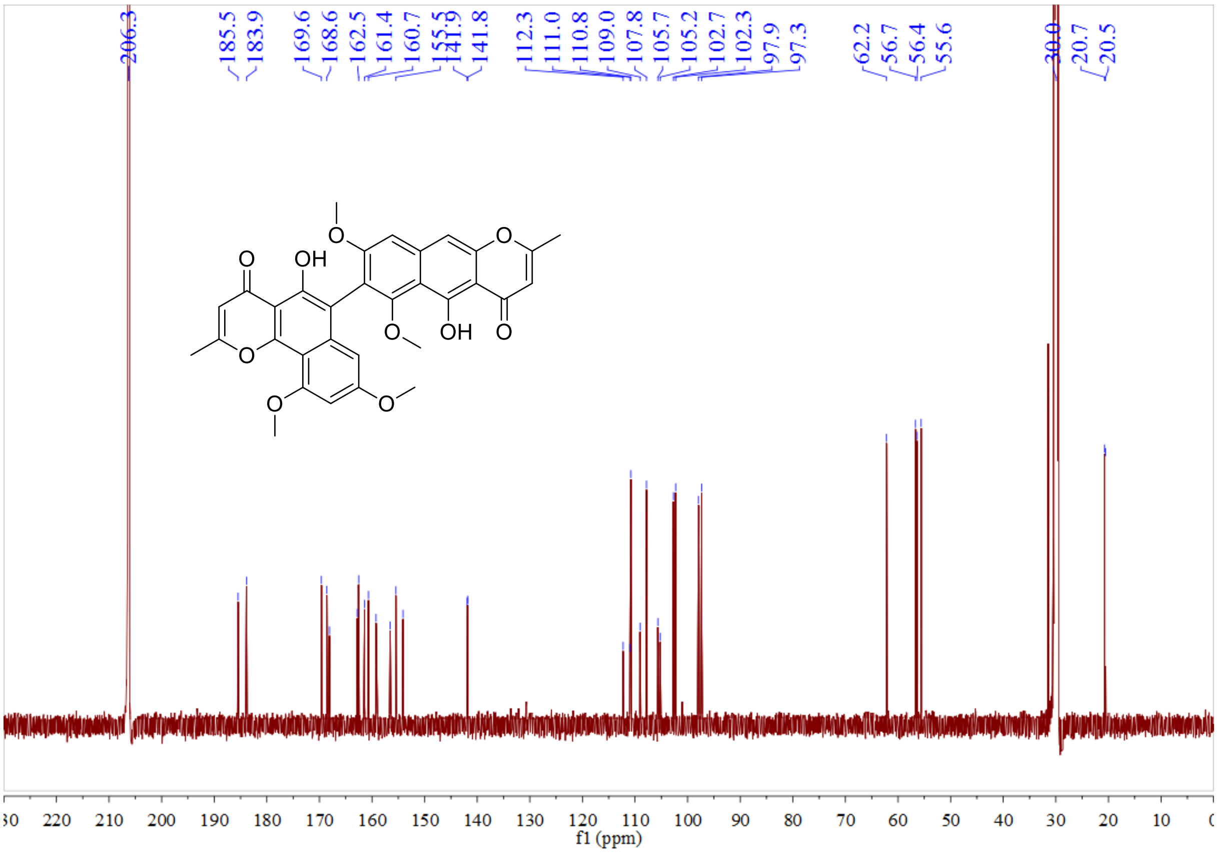

Figure S17. The ^{13}C NMR spectrum (150 MHz) of **4** in acetone-d_6

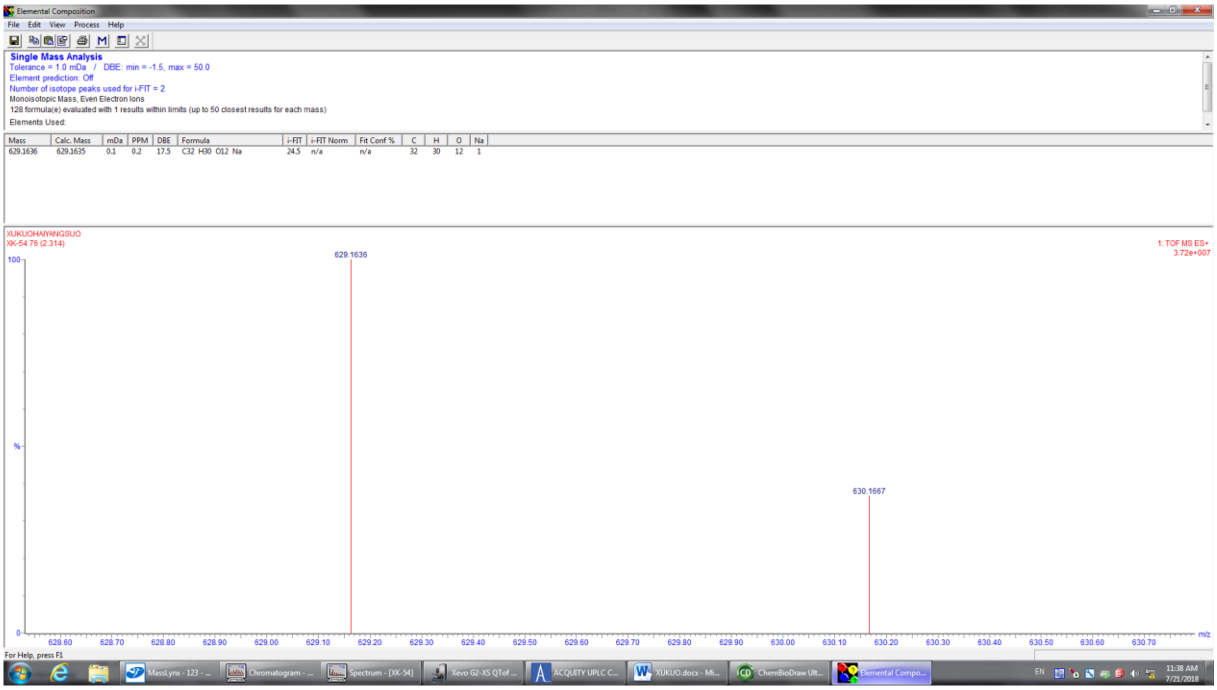

Figure S18. The HR-ESI-MS data of **5**

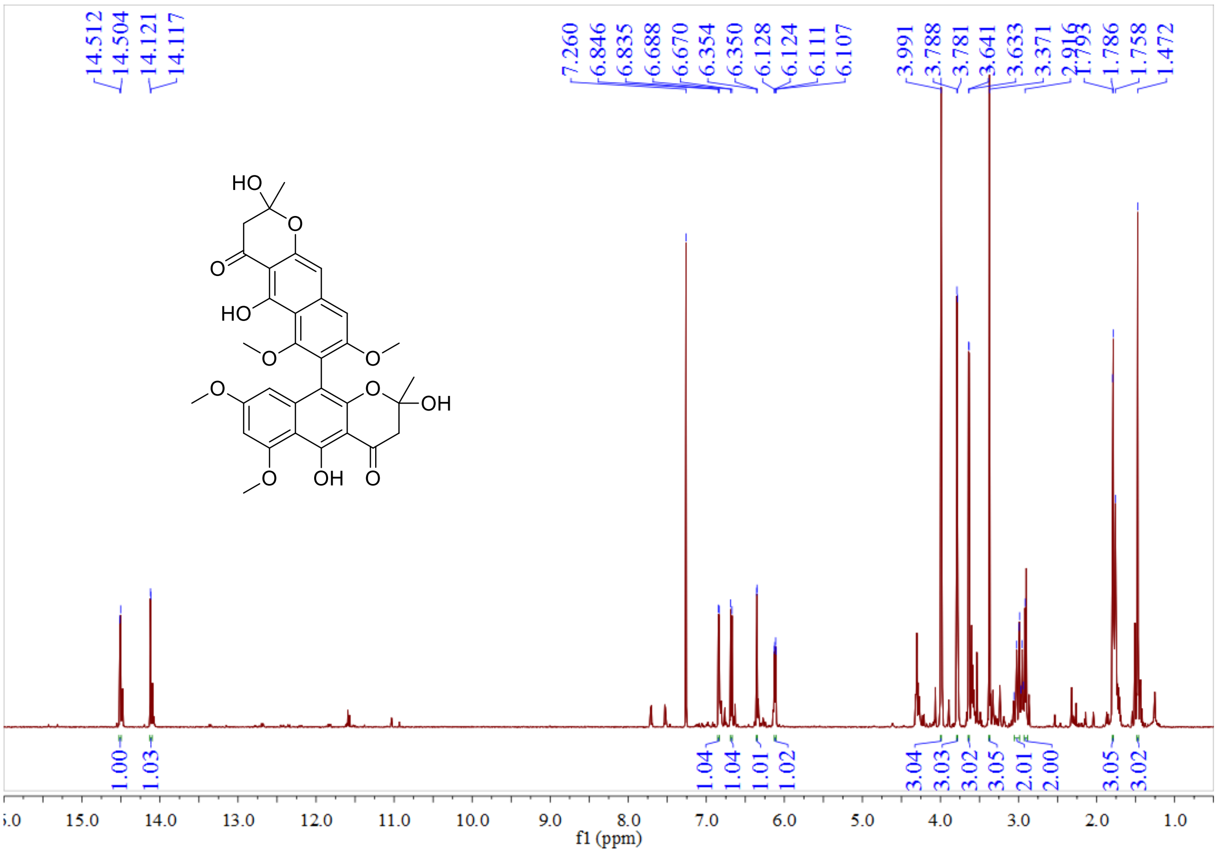

Figure S19. The ^{1}H NMR spectrum (600 MHz) of **5** in $CDCl_3$

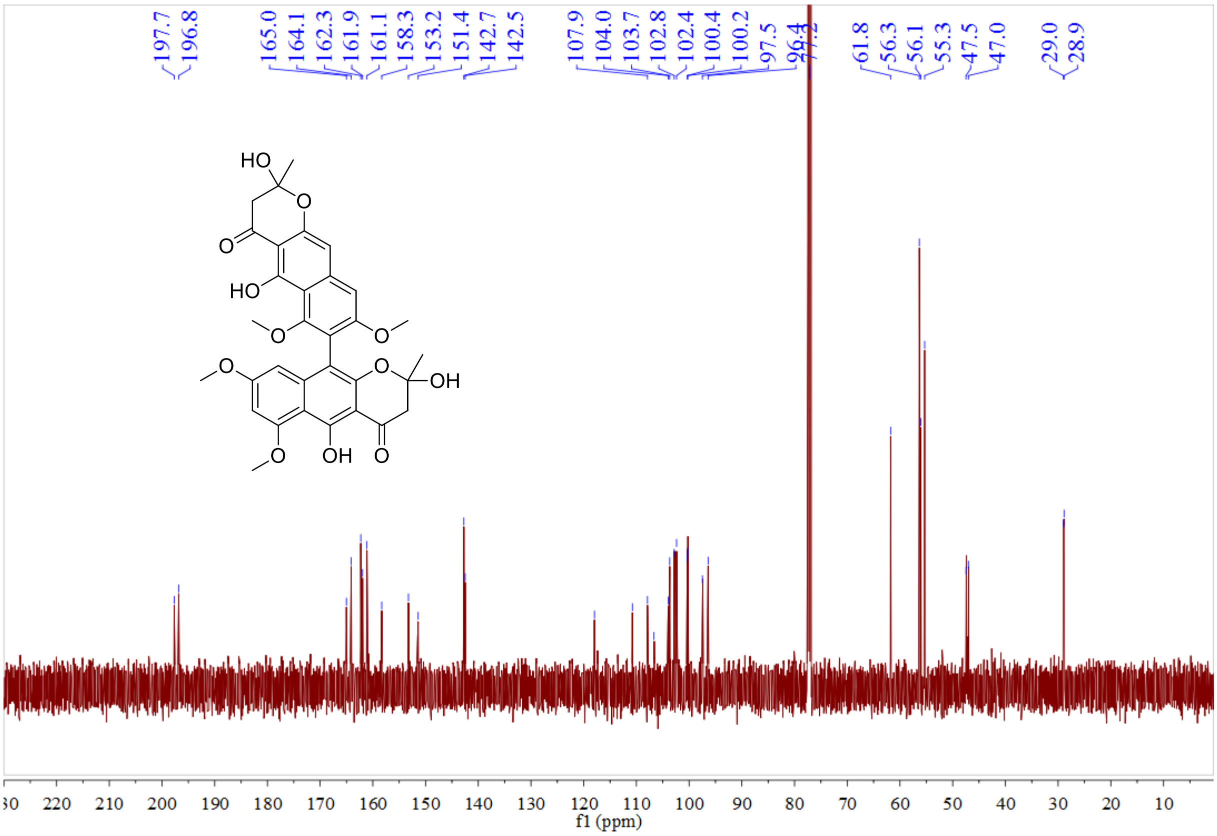

Figure S20. The ^{13}C NMR spectrum (150 MHz) of **5** in $CDCl_3$

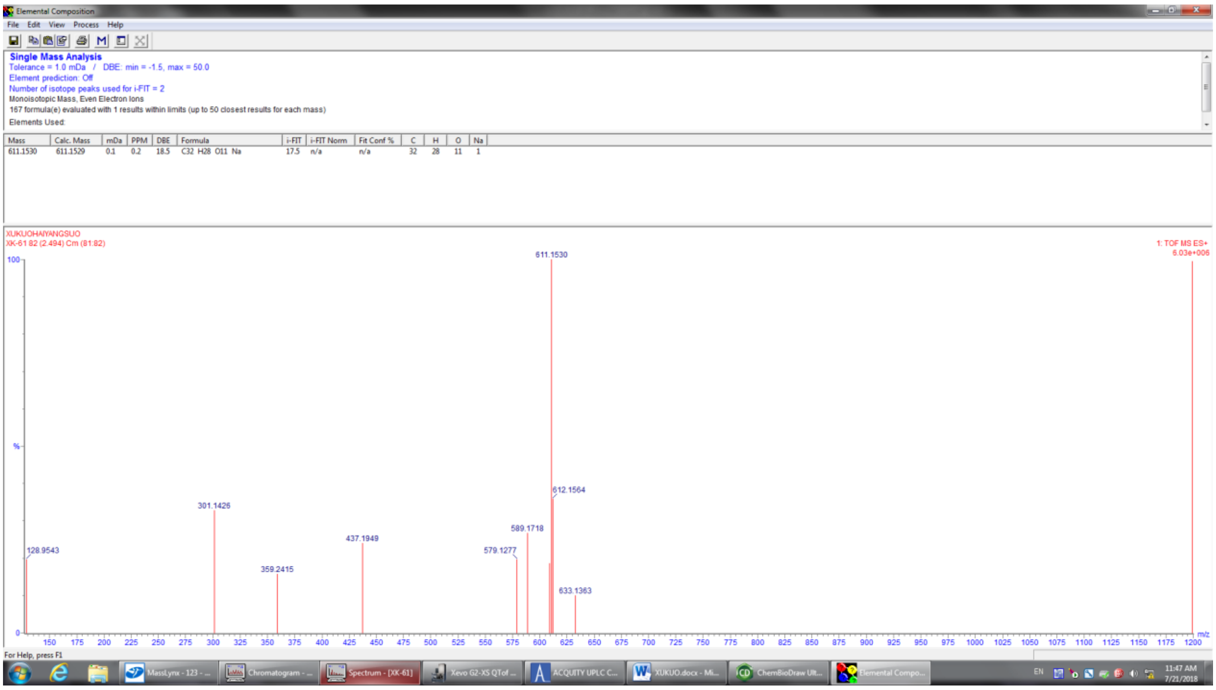

Figure S21. The HR-ESI-MS data of **6**

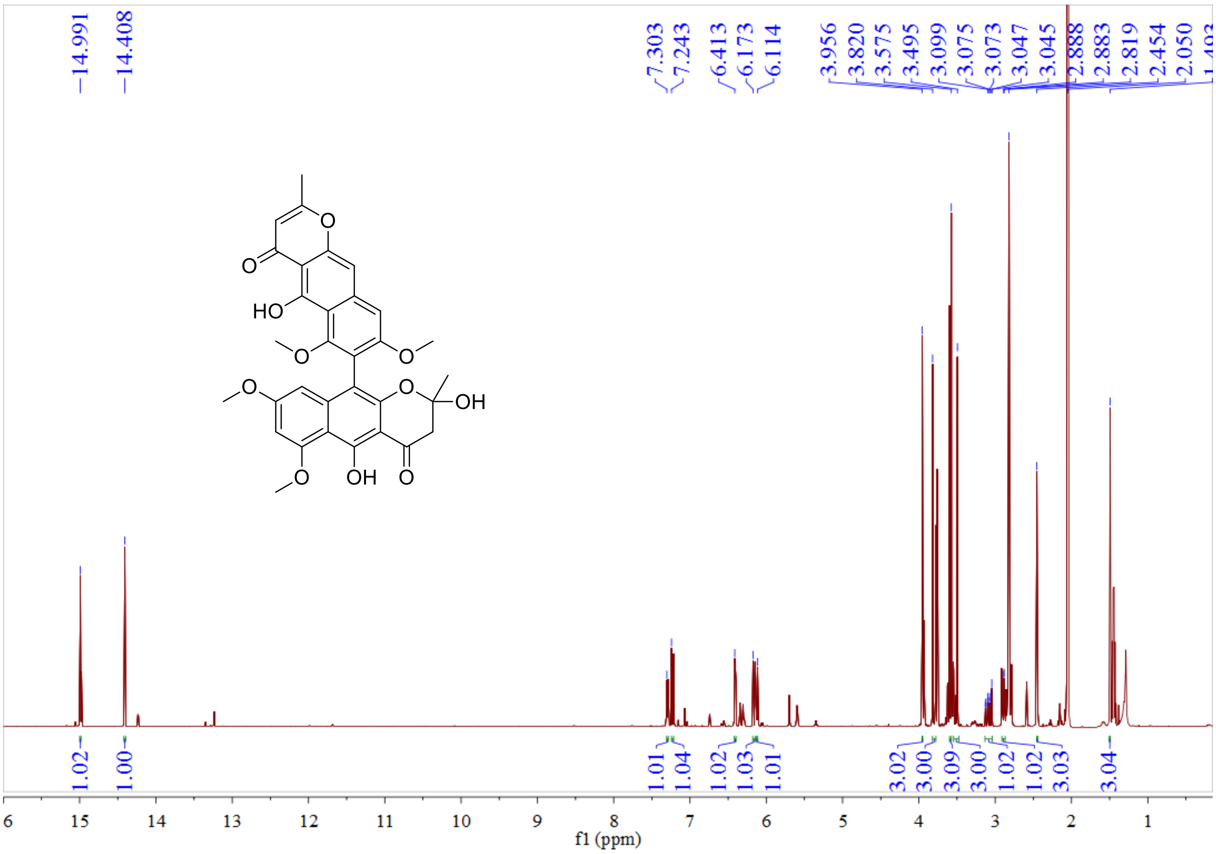

Figure S22. The ^{1}H NMR spectrum (600 MHz) of **6** in acetone-d_6

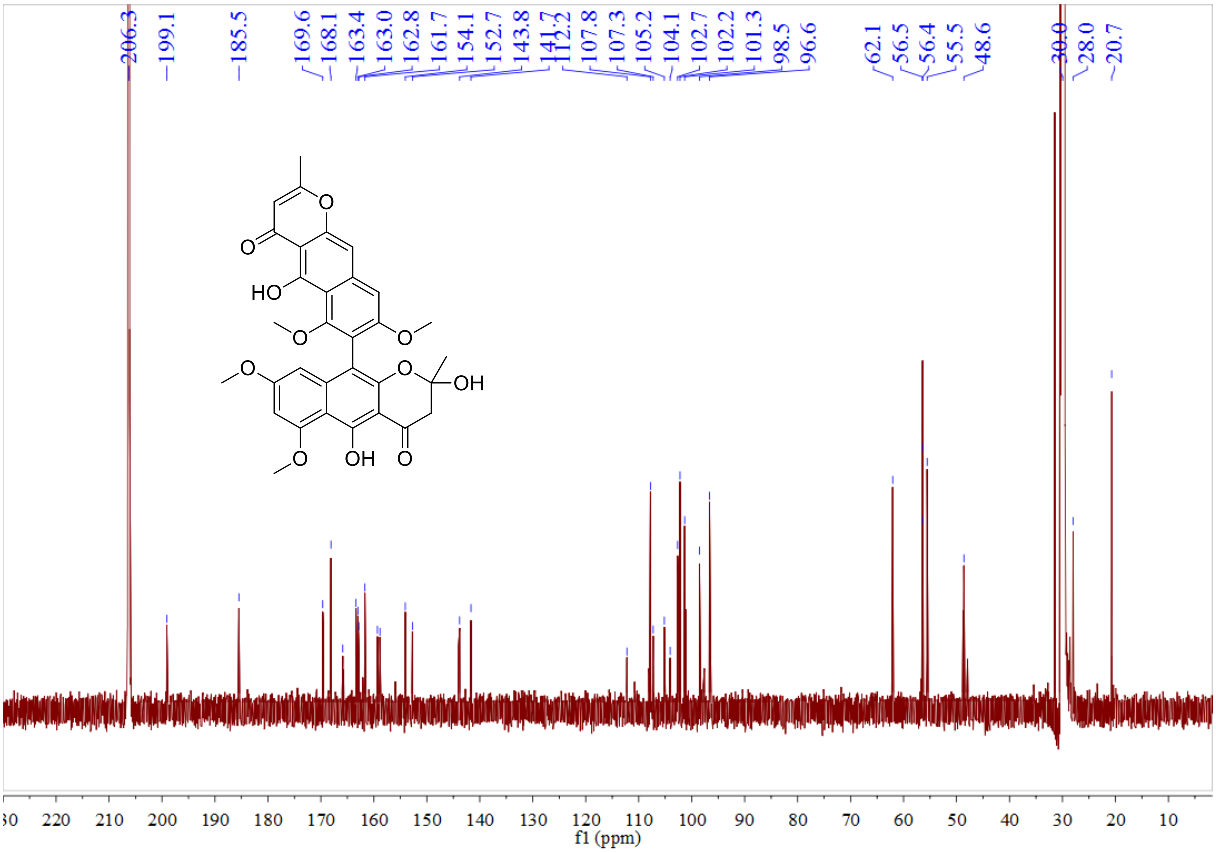

Figure S23. The ^{13}C NMR spectrum (150 MHz) of **6** in acetone-d_6

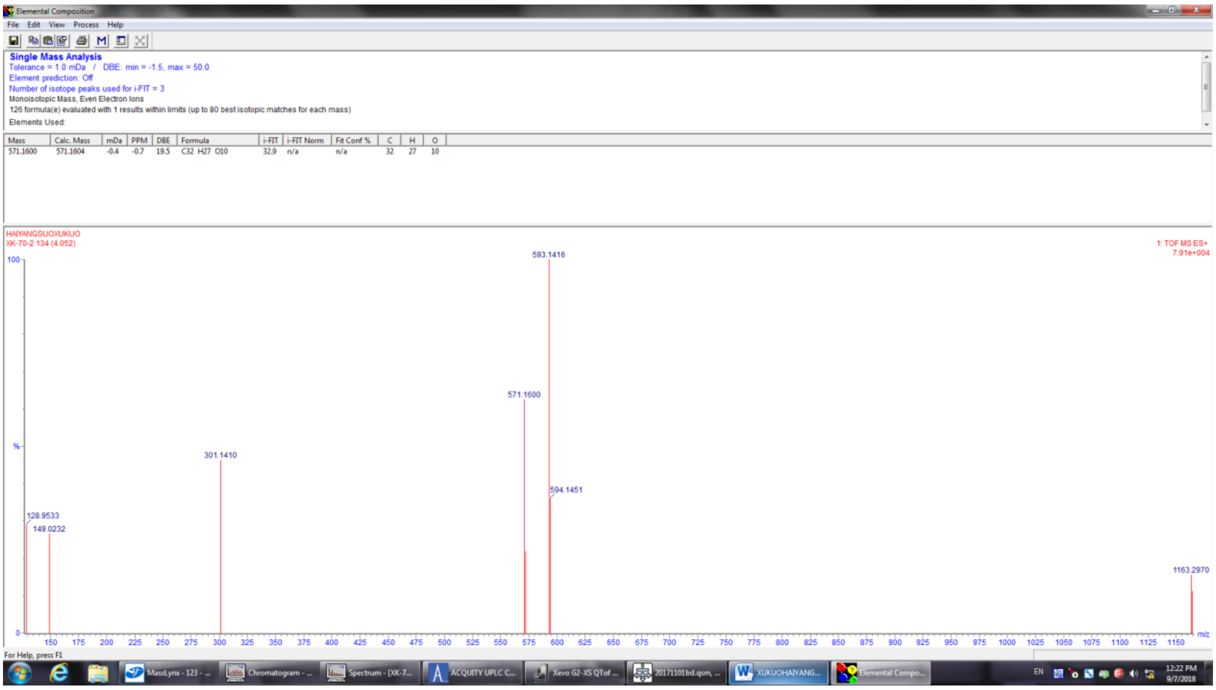

Figure S24. The HR-ESI-MS data of **7**

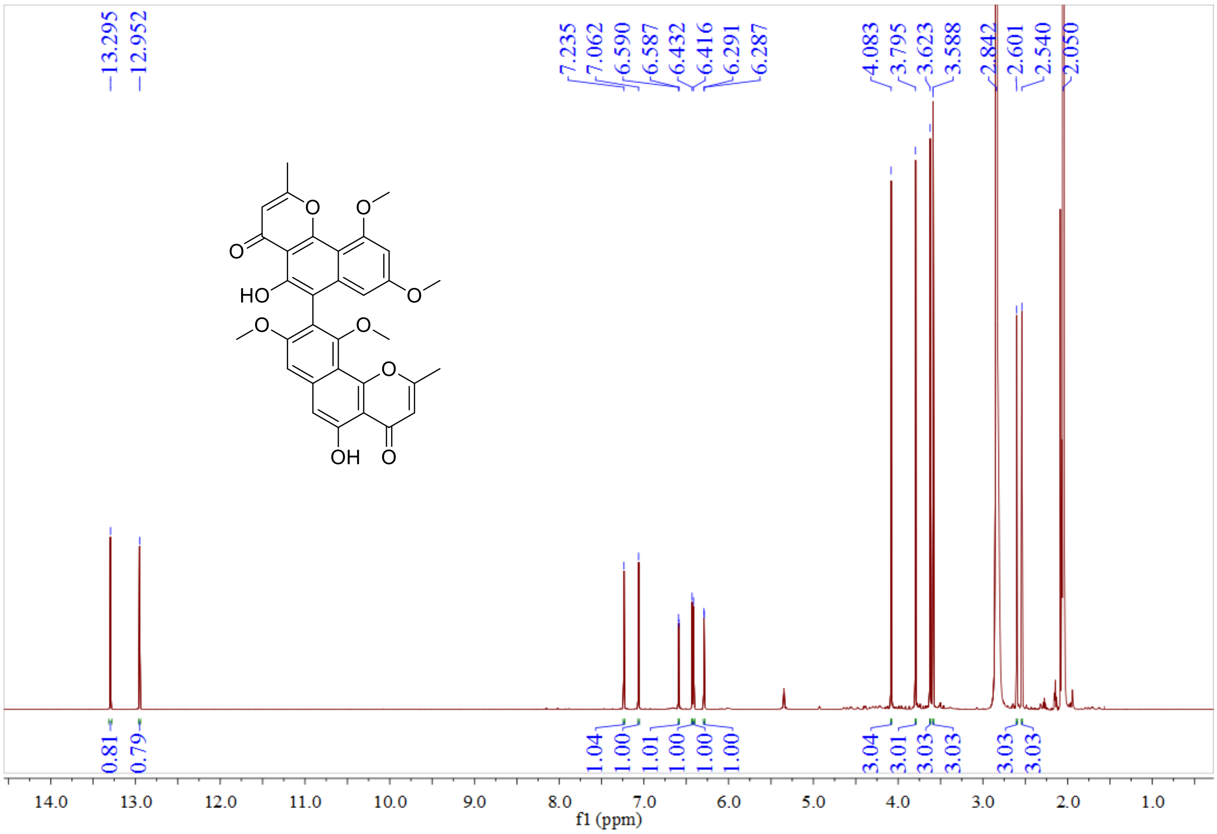

Figure S25. The ^{1}H NMR spectrum (600 MHz) of **7** in acetone-d_6

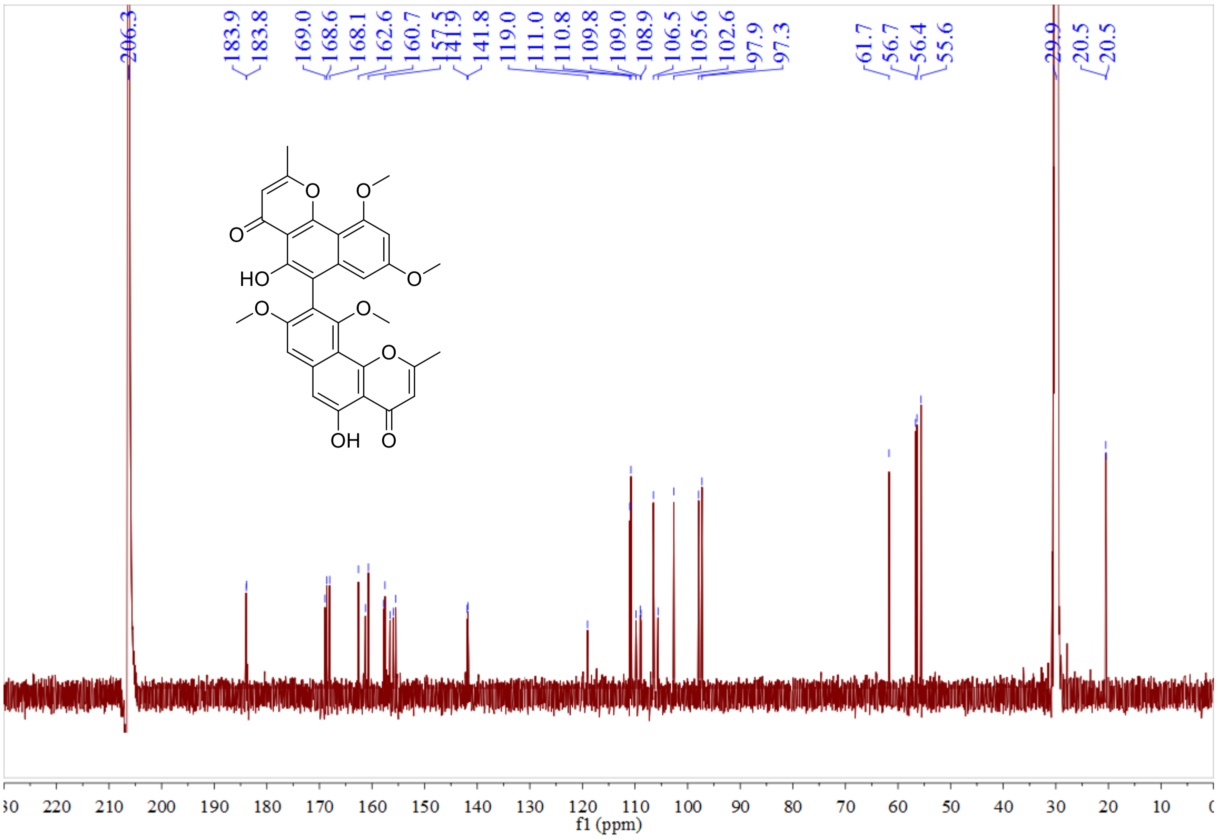

Figure S26. The ^{13}C NMR spectrum (150 MHz) of **7** in acetone-d_6

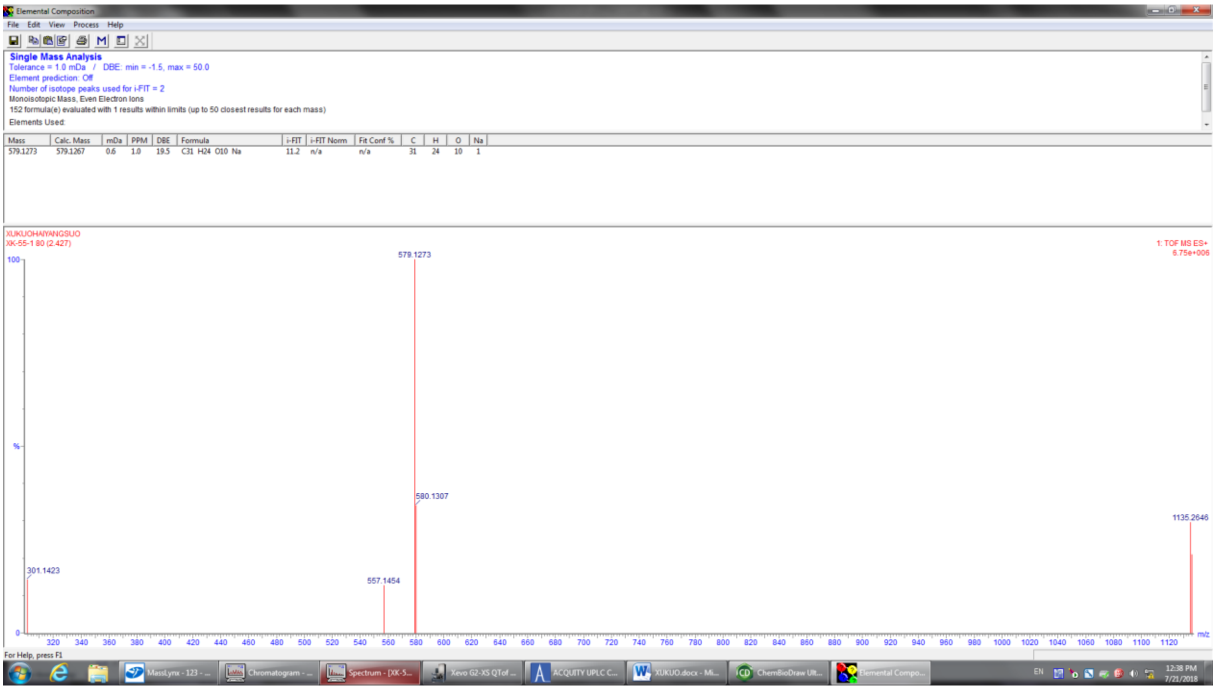

Figure S27. The HR-ESI-MS data of **8**

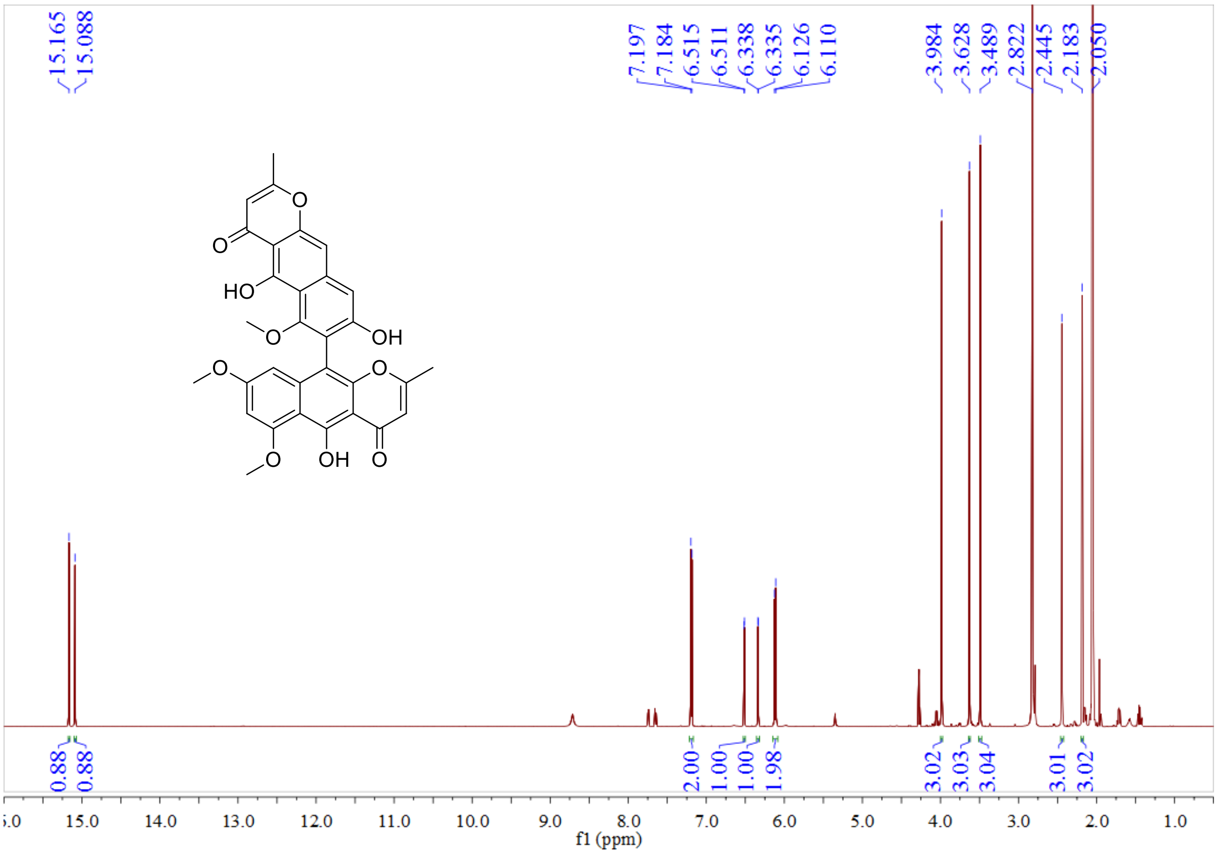

Figure S28. The ^{1}H NMR spectrum (600 MHz) of **8** in acetone-d_6

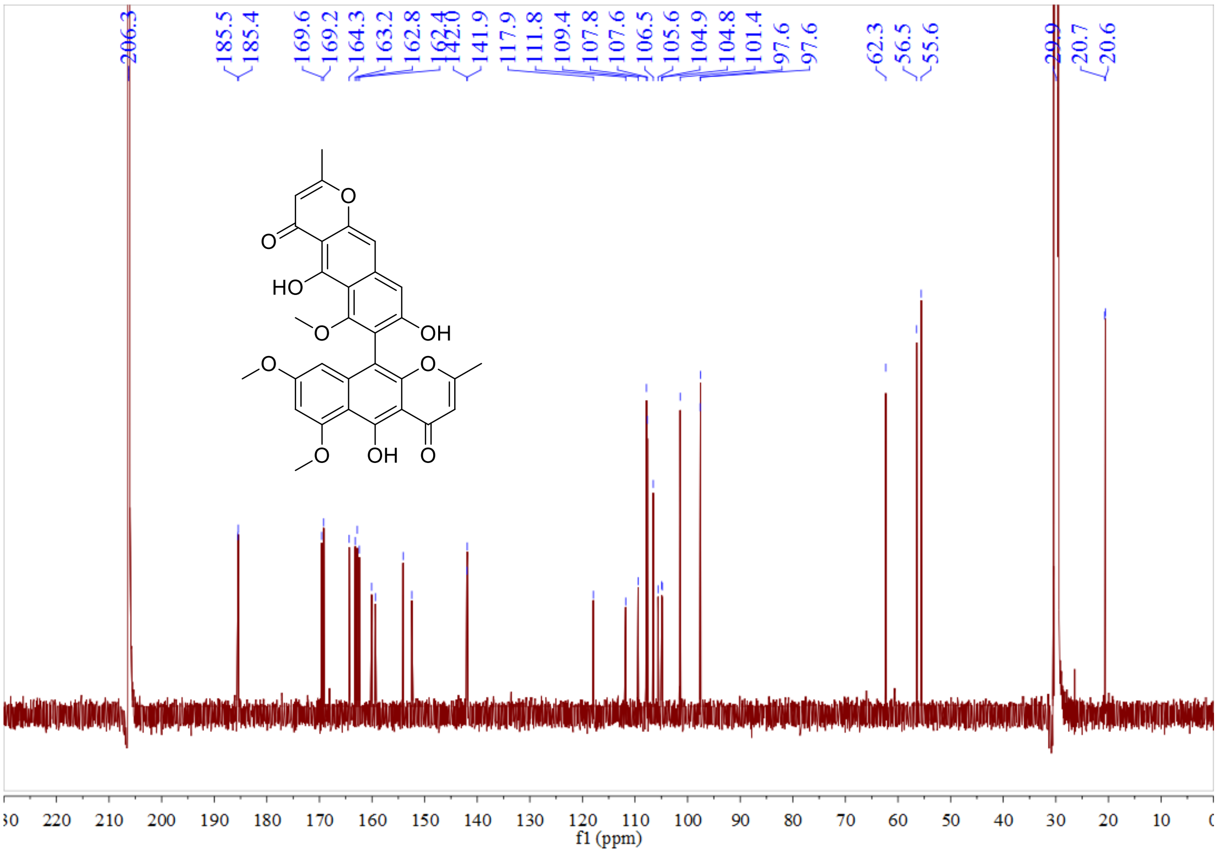

Figure S29. The ^{13}C NMR spectrum (150 MHz) of **8** in acetone-d_6

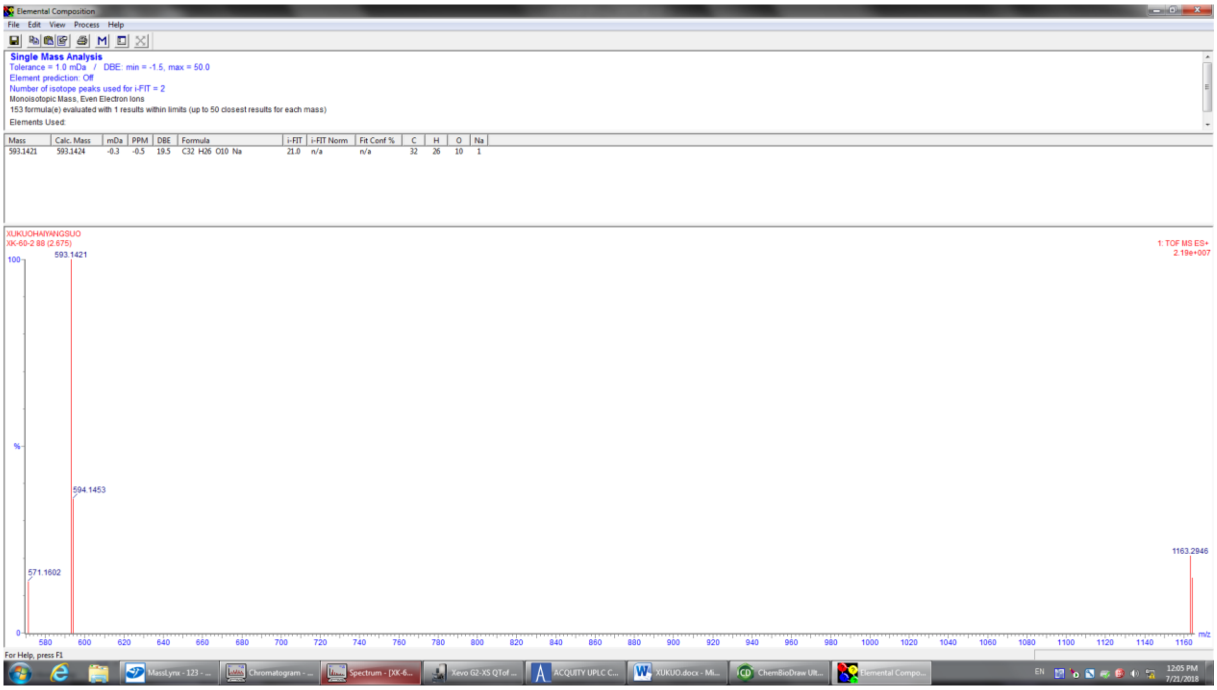

Figure S30. The HR-ESI-MS data of **9**

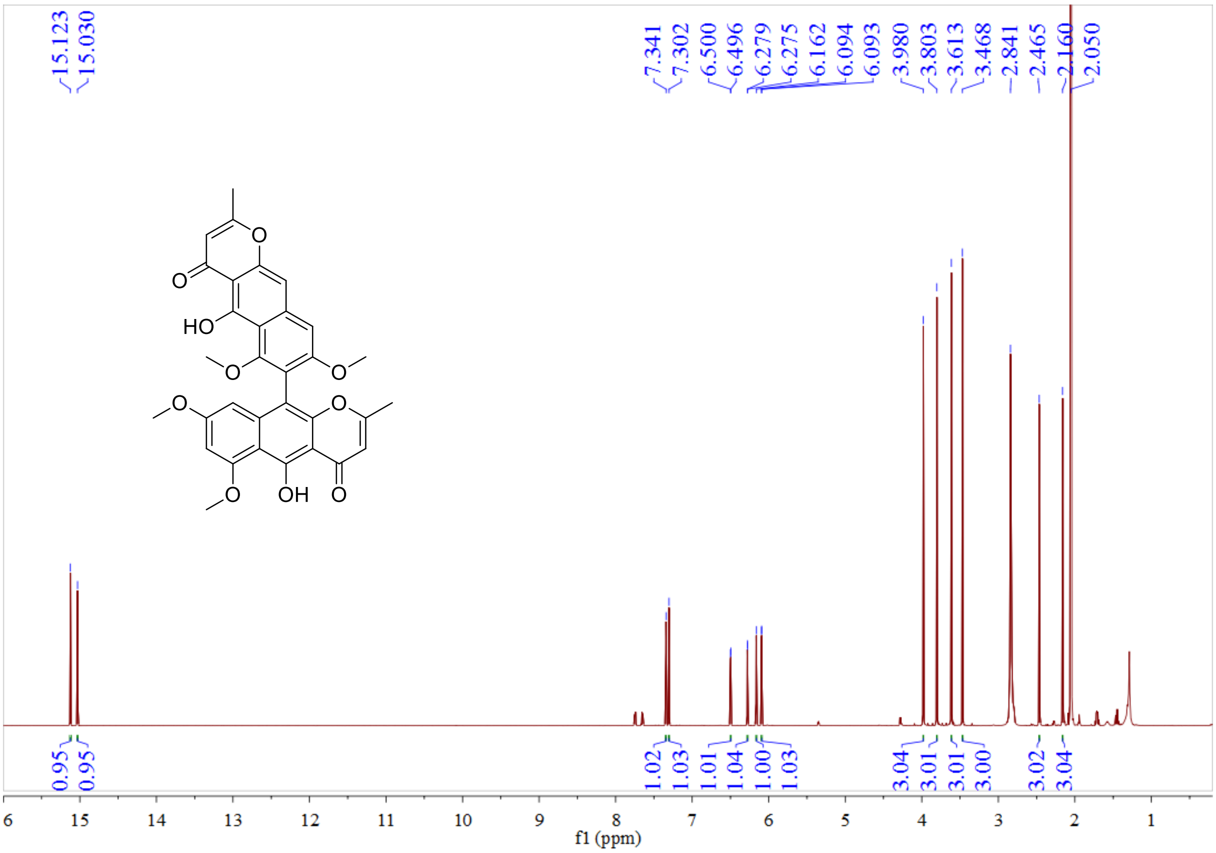

Figure S31. The ^{1}H NMR spectrum (600 MHz) of **9** in acetone-d_6

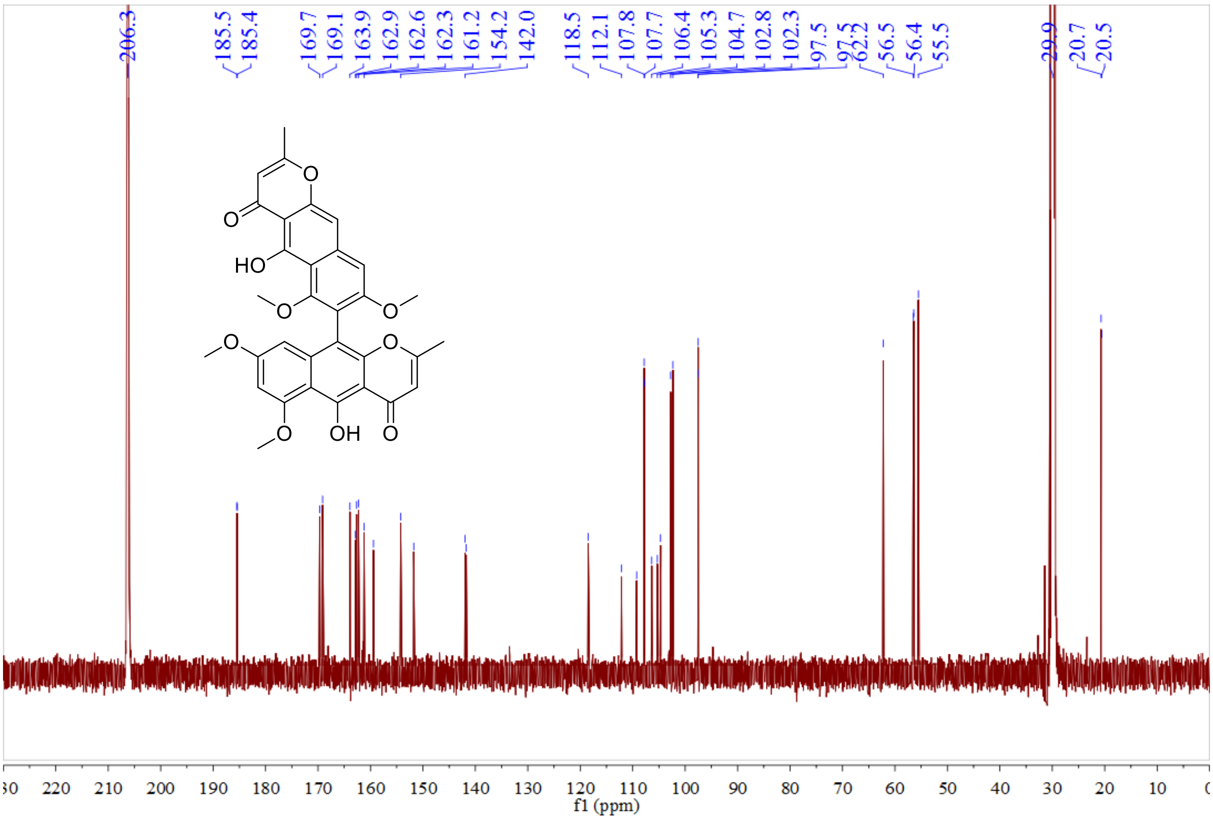

Figure S32. The ^{13}C NMR spectrum (150 MHz) of **9** in acetone-d_6

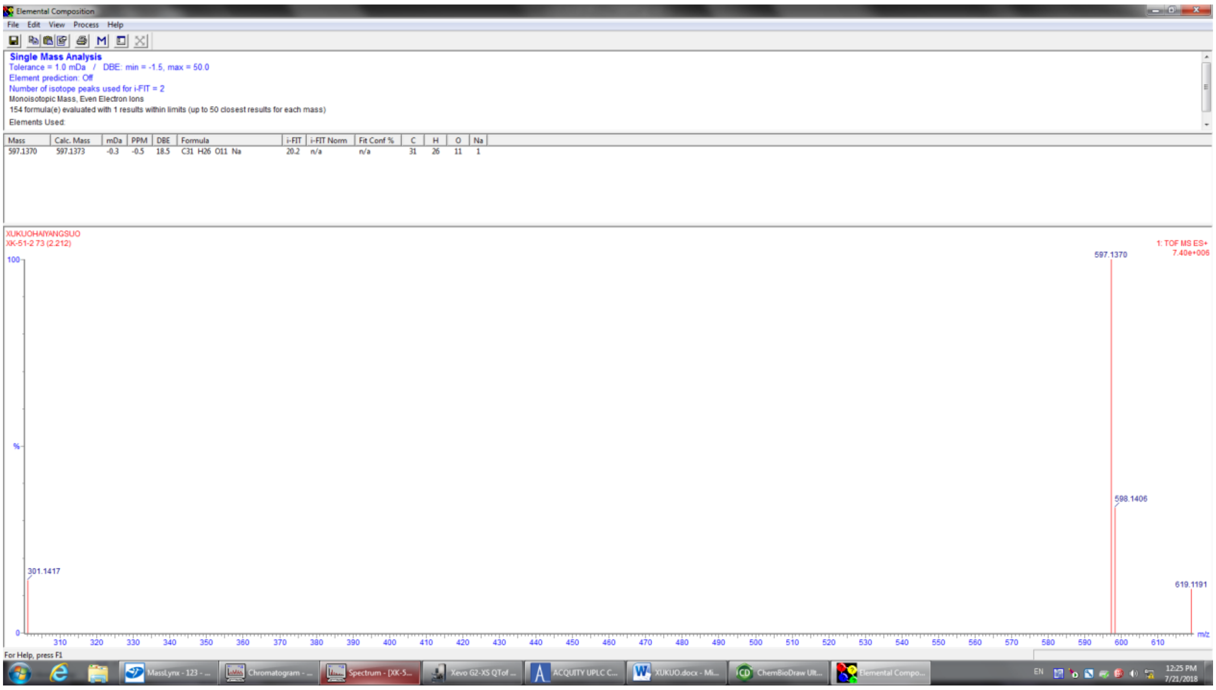

Figure S33. The HR-ESI-MS data of **10**

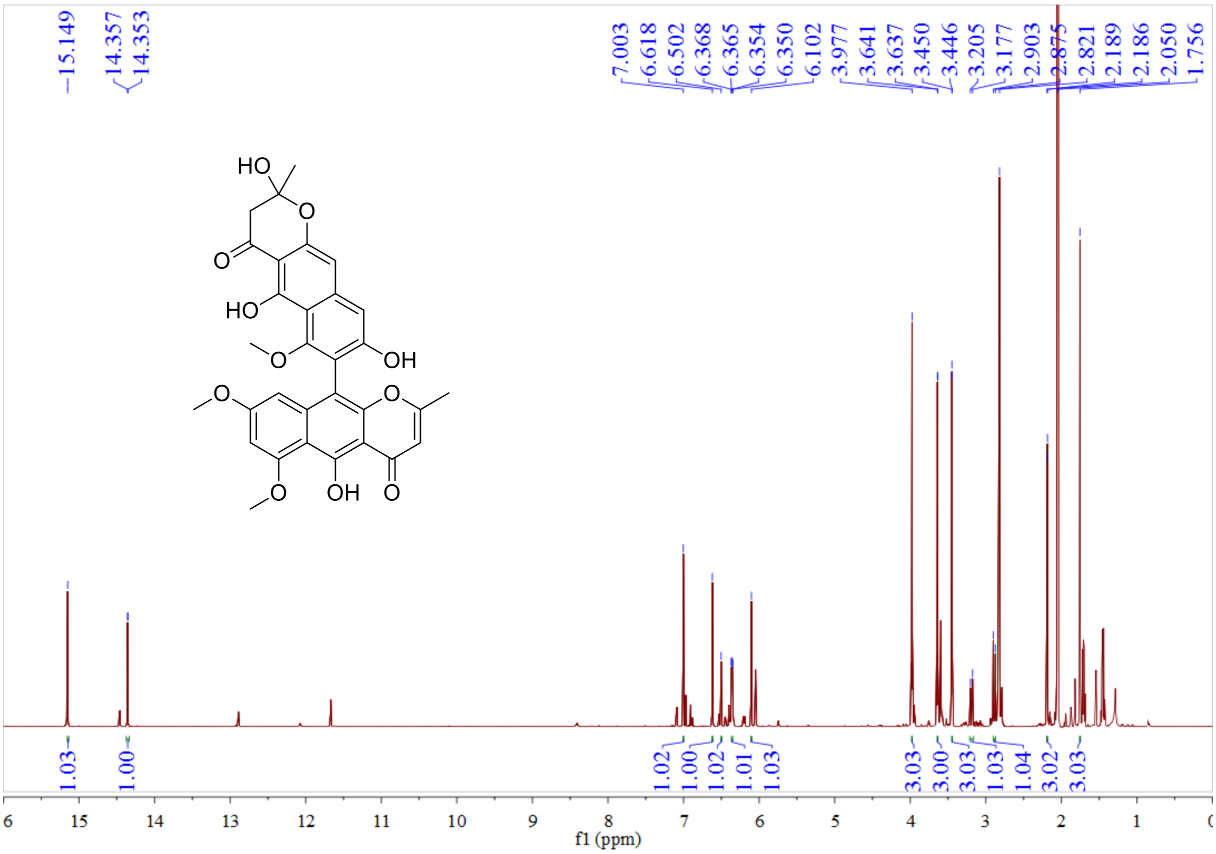

Figure S34. The ^{1}H NMR spectrum (600 MHz) of **10** in acetone-d_6

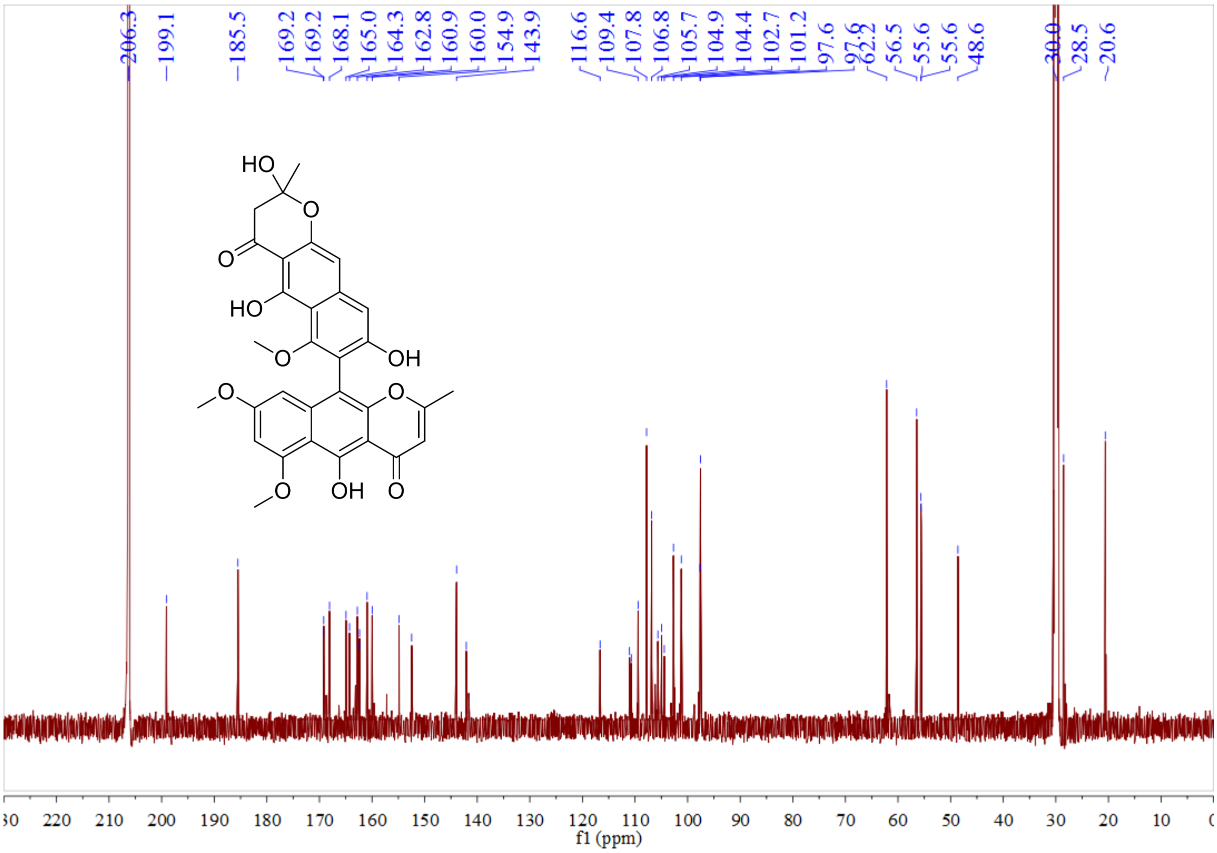

Figure S35. The ^{13}C NMR spectrum (150 MHz) of **10** in acetone-d_6

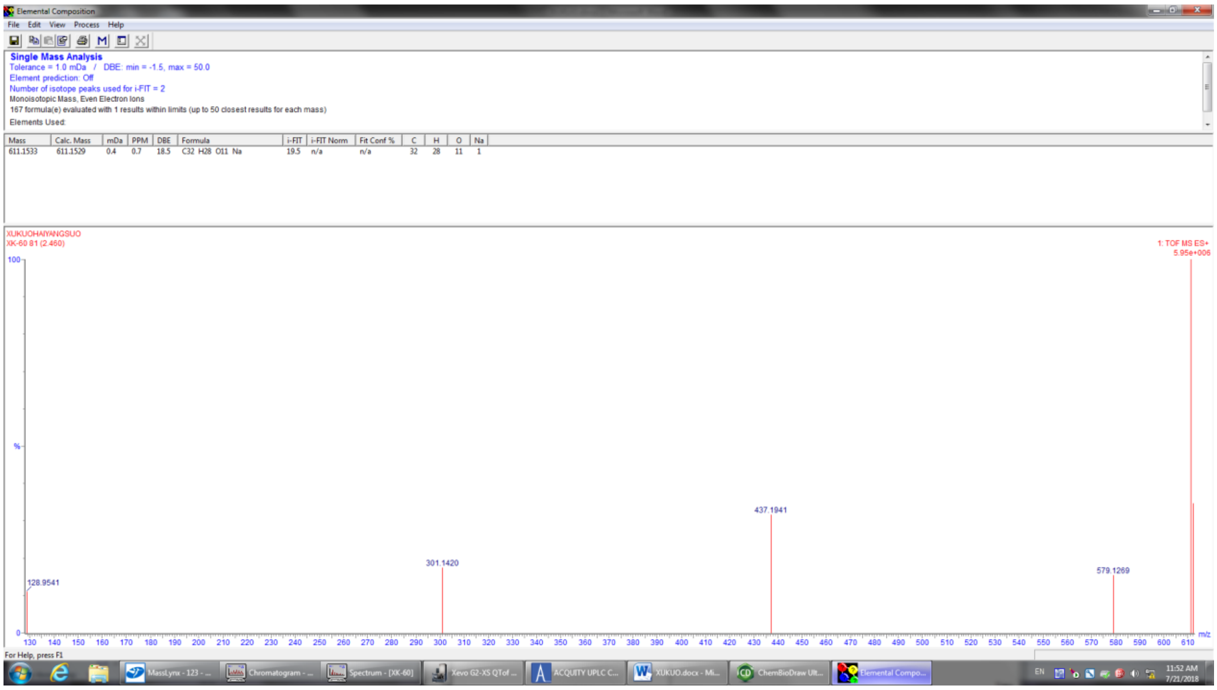

Figure S36. The HR-ESI-MS data of **11**

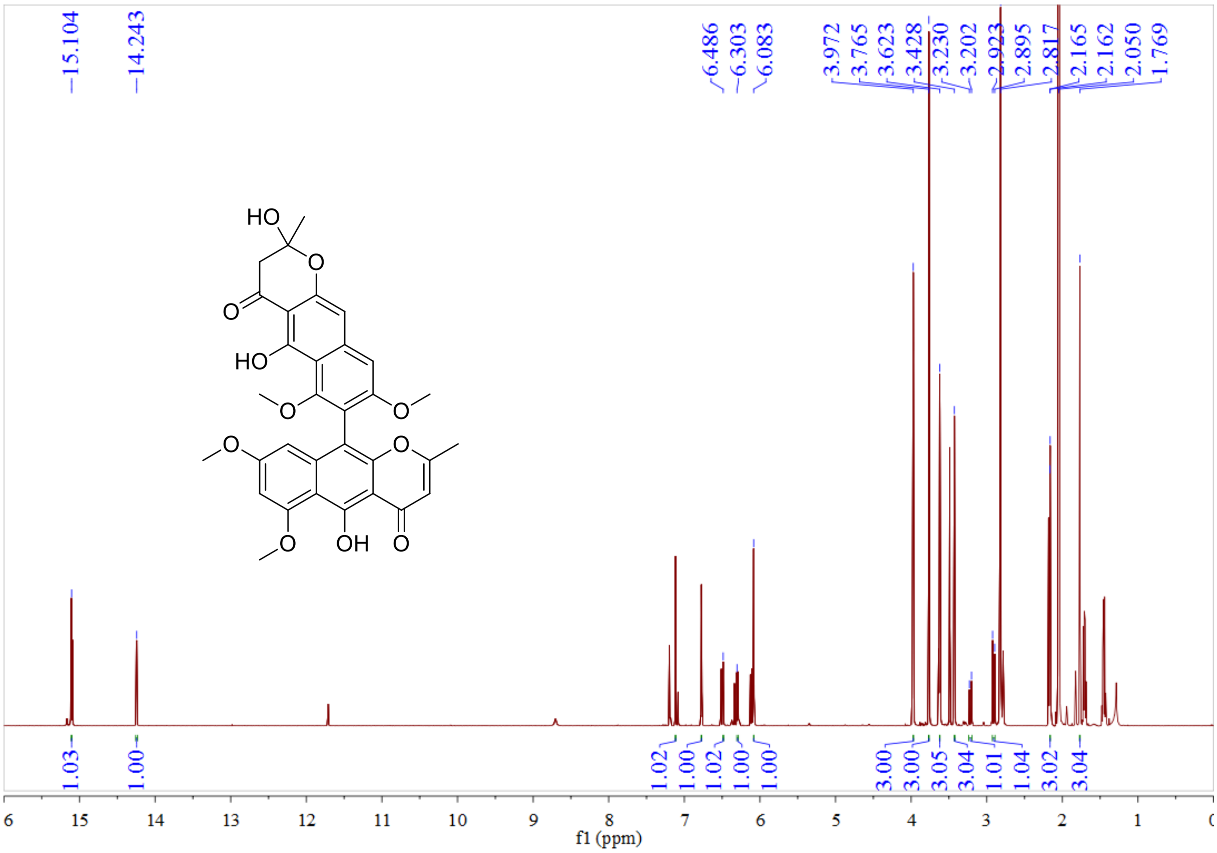

Figure S37. The ^{1}H NMR spectrum (600 MHz) of **11** in acetone-d_6

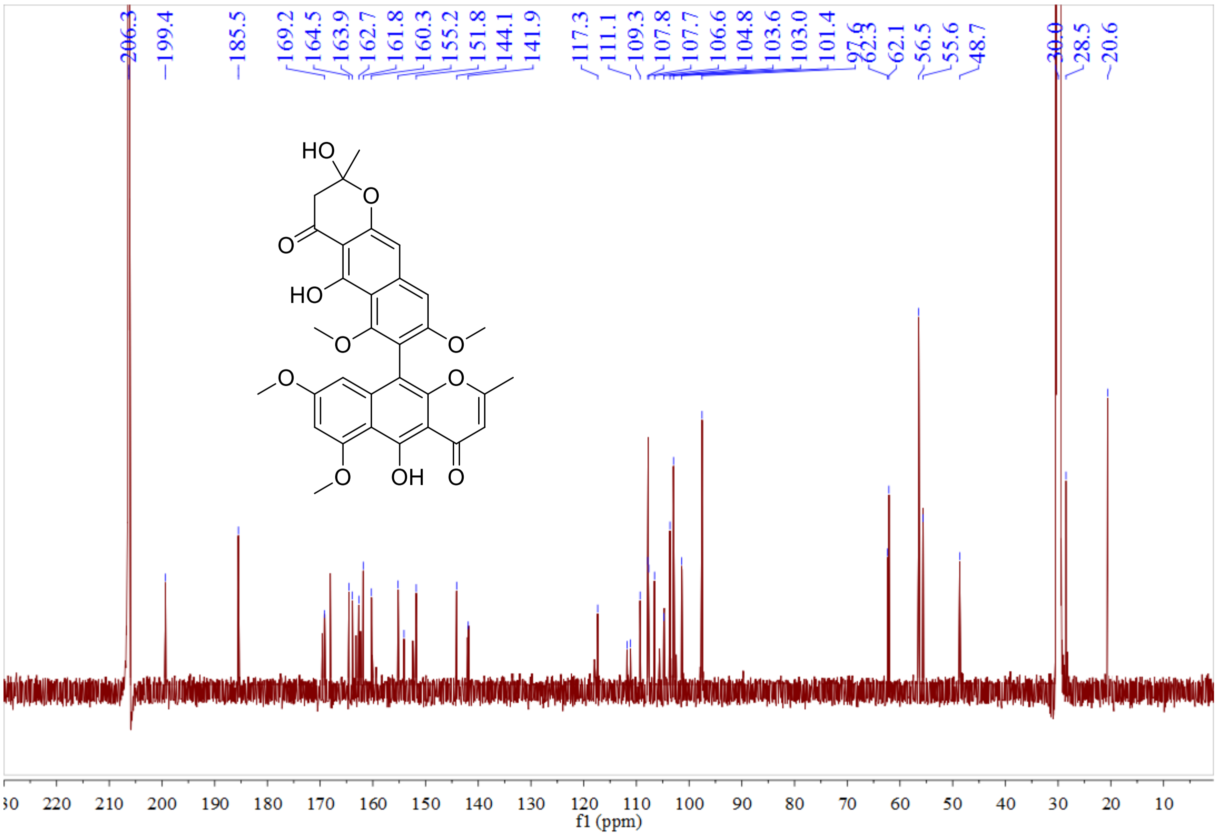

Figure S38. The ^{13}C NMR spectrum (150 MHz) of **11** in acetone-d_6

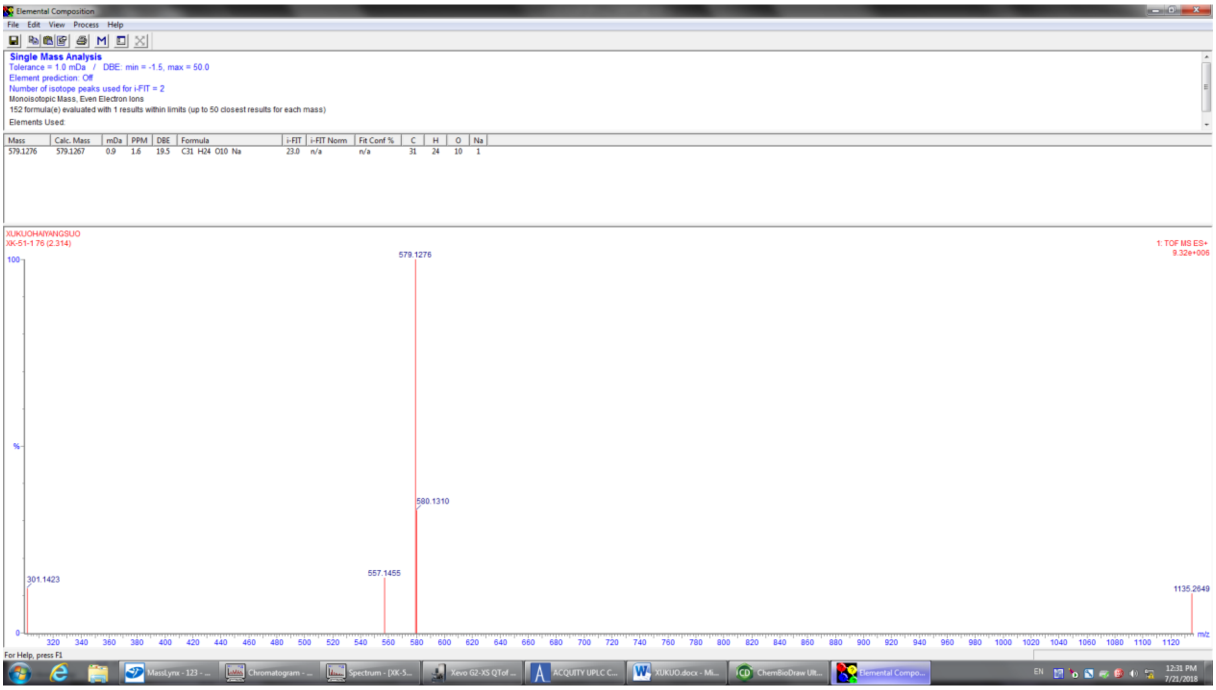

Figure S39. The HR-ESI-MS data of **12**

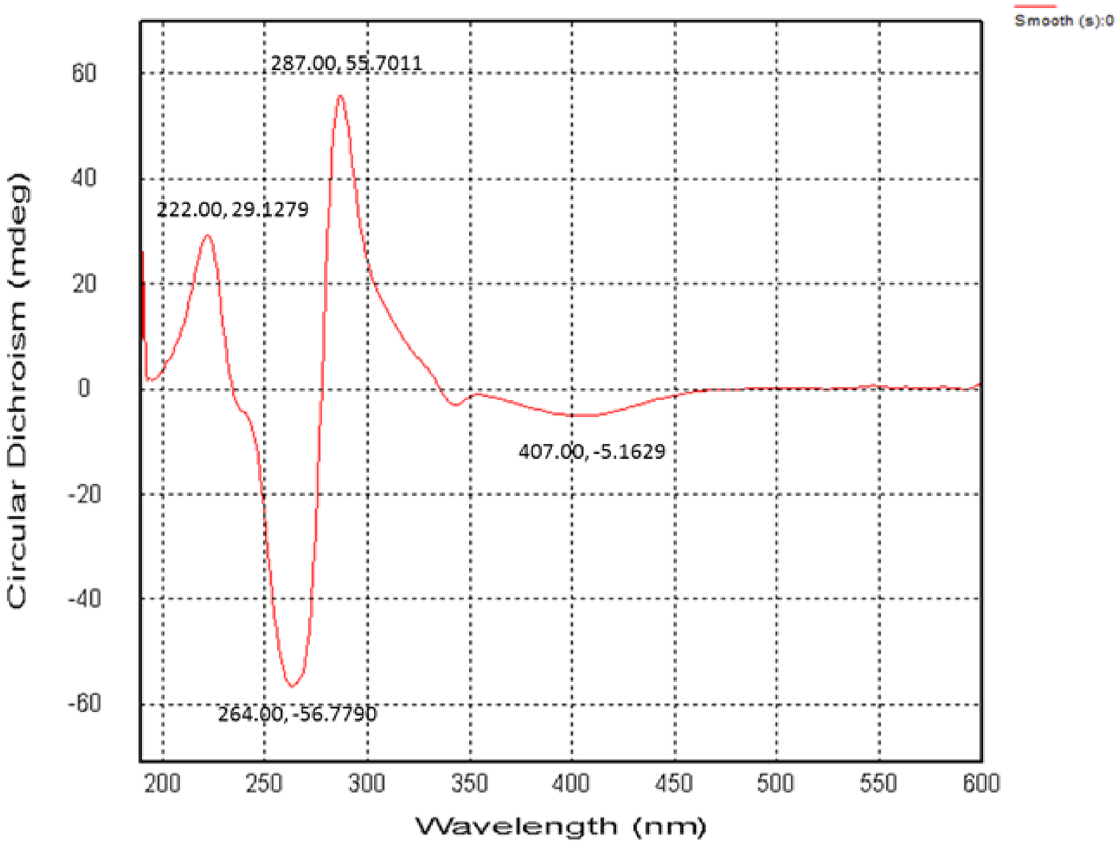

Figure S40. The ECD spectrum of **12** in MeOH

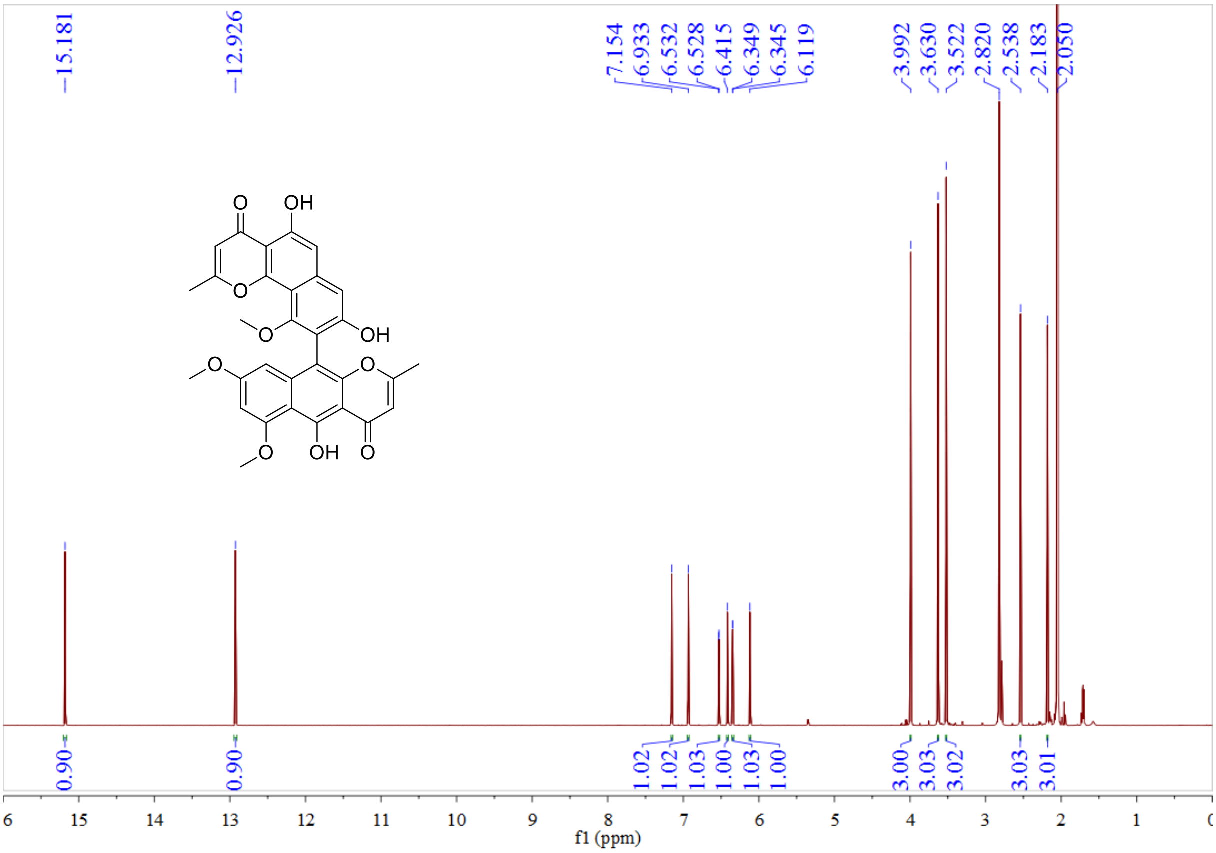

Figure S41. The ^{1}H NMR spectrum (600 MHz) of **12** in acetone-d_6

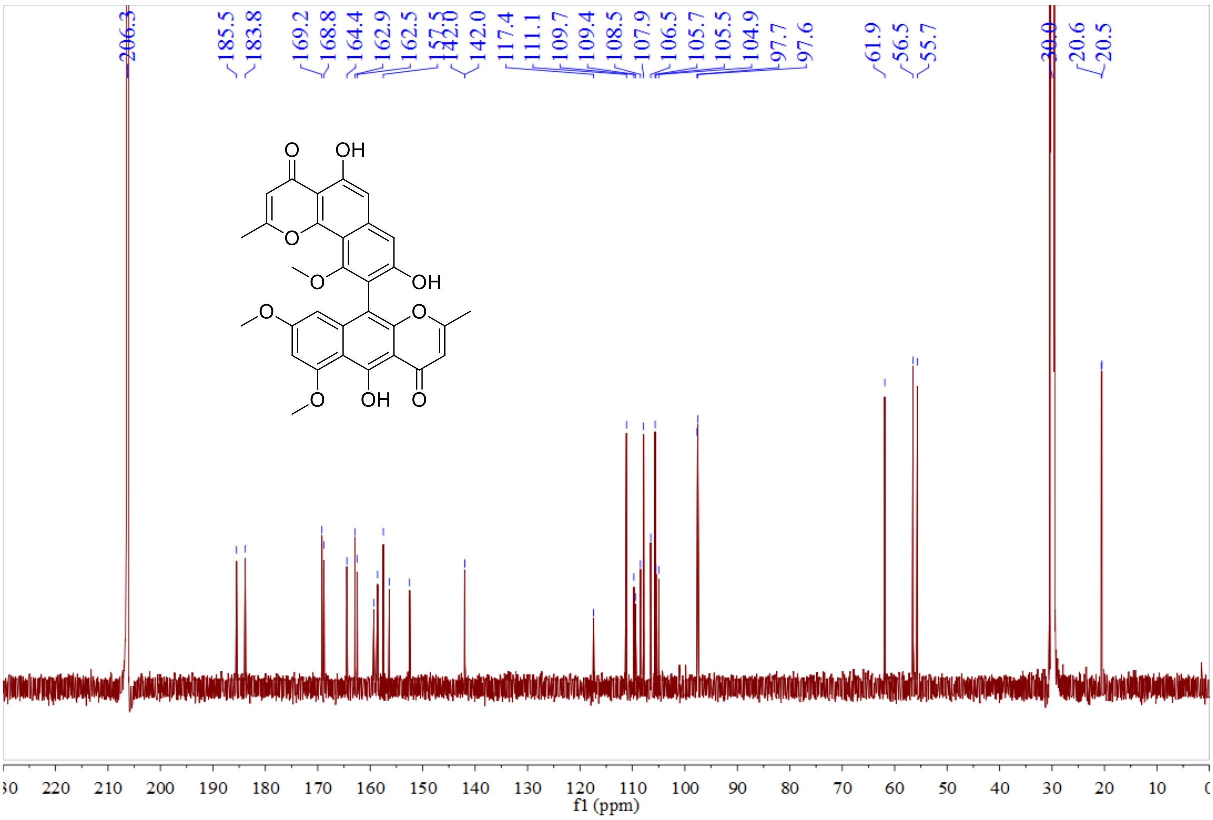

Figure S42. The ^{13}C NMR spectrum (150 MHz) of **12** in acetone-d_6

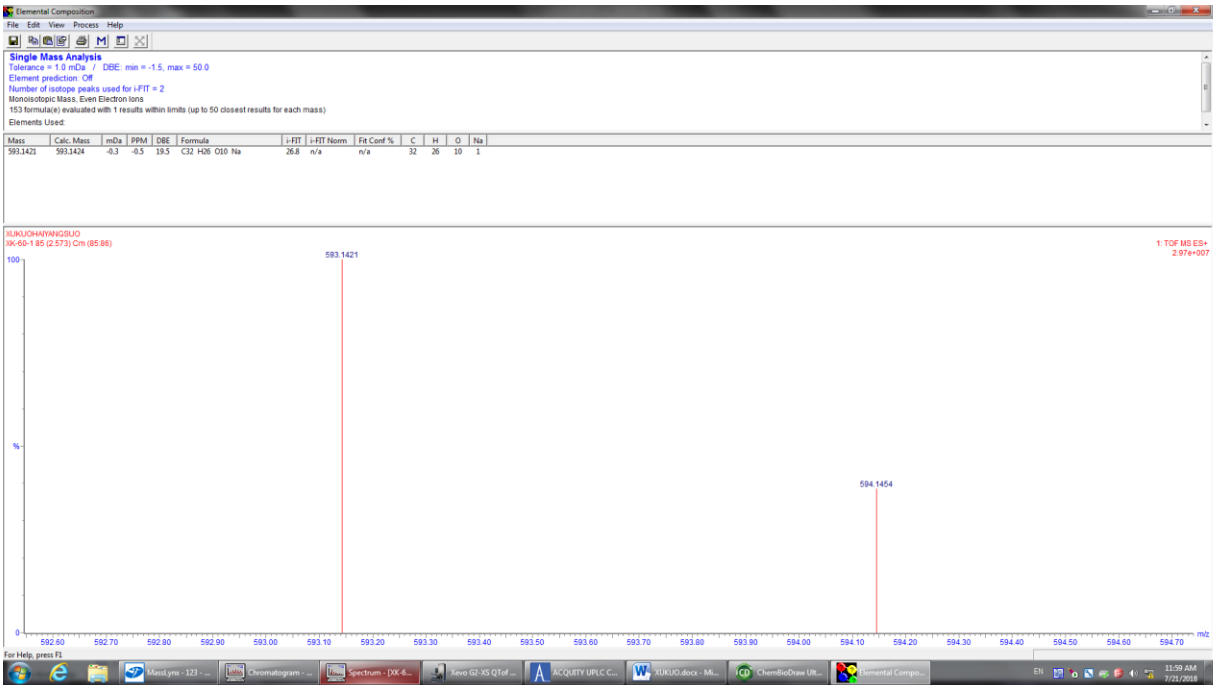

Figure S43. The HR-ESI-MS data of **13**

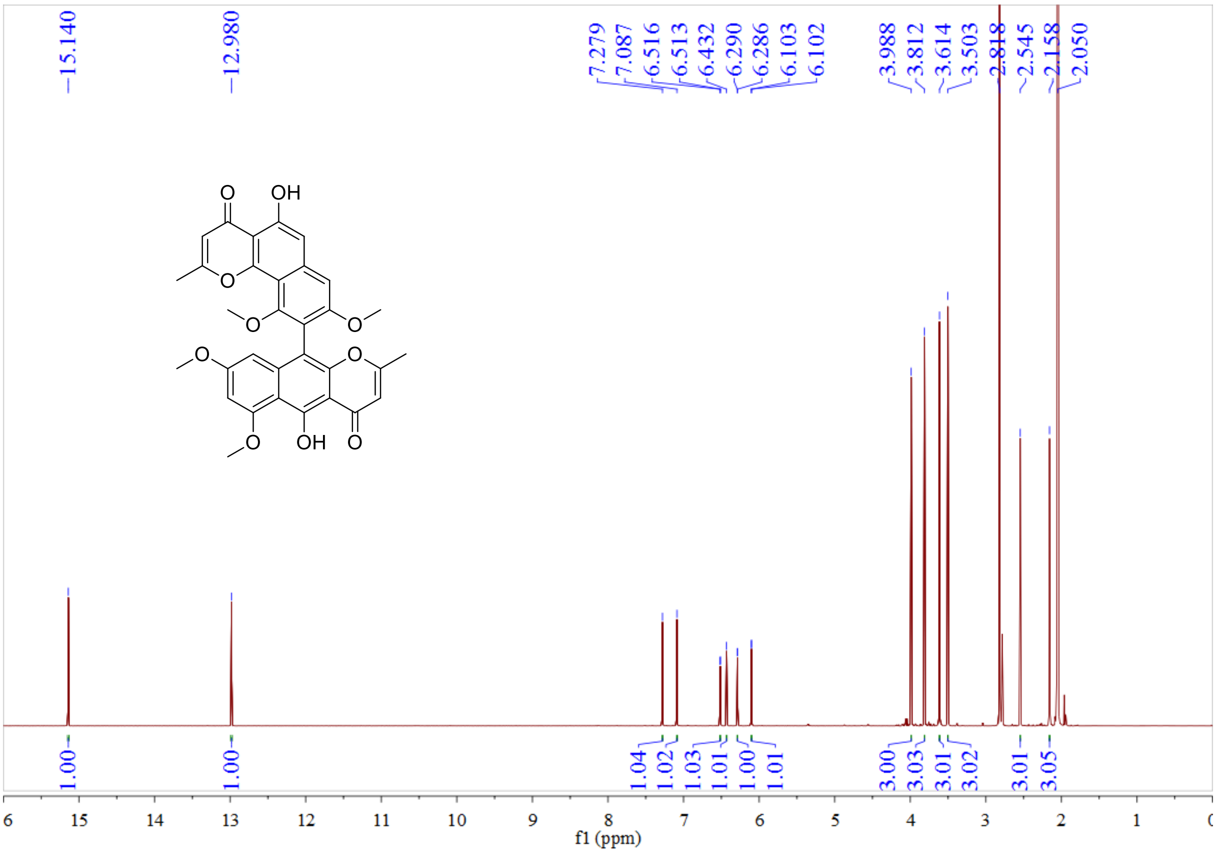

Figure S44. The ^{1}H NMR spectrum (600 MHz) of **13** in acetone-d_6

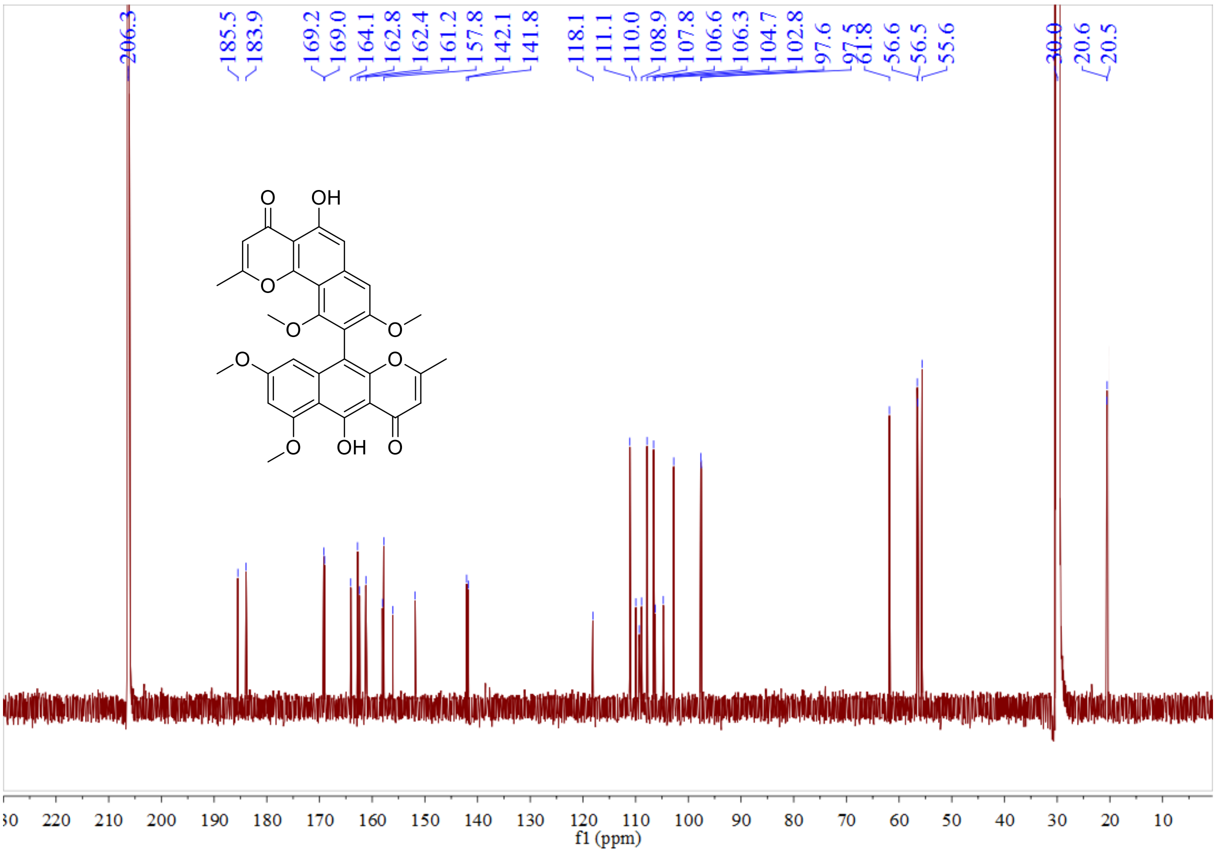

Figure S45. The ^{13}C NMR spectrum (150 MHz) of **13** in acetone-d_6

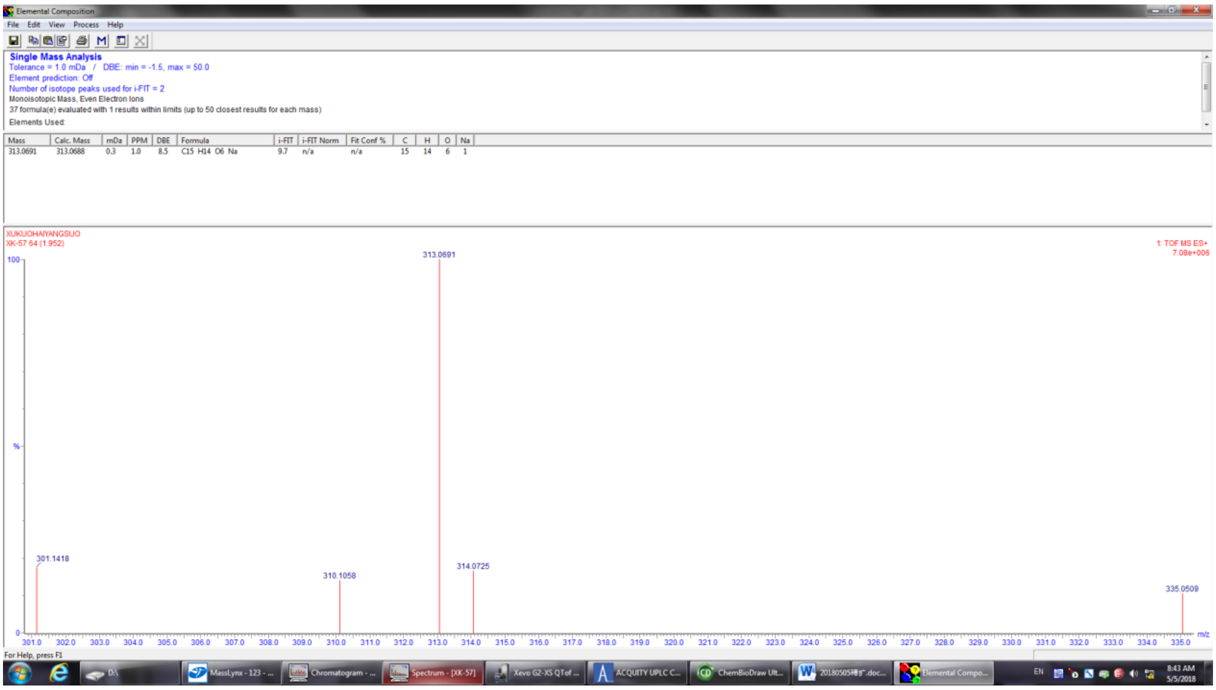

Figure S46. The HR-ESI-MS data of **14**

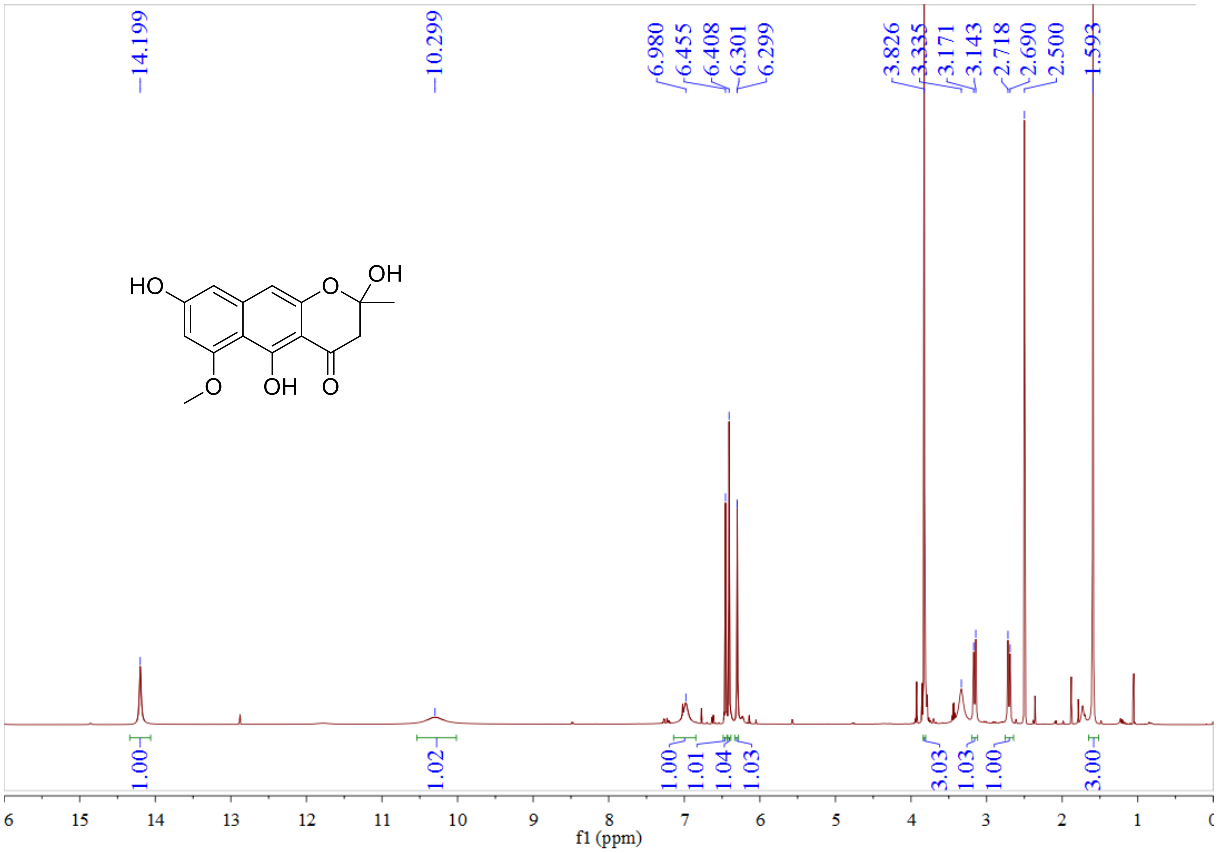

Figure S47. The ^{1}H NMR spectrum (600 MHz) of **14** in DMSO-d_6

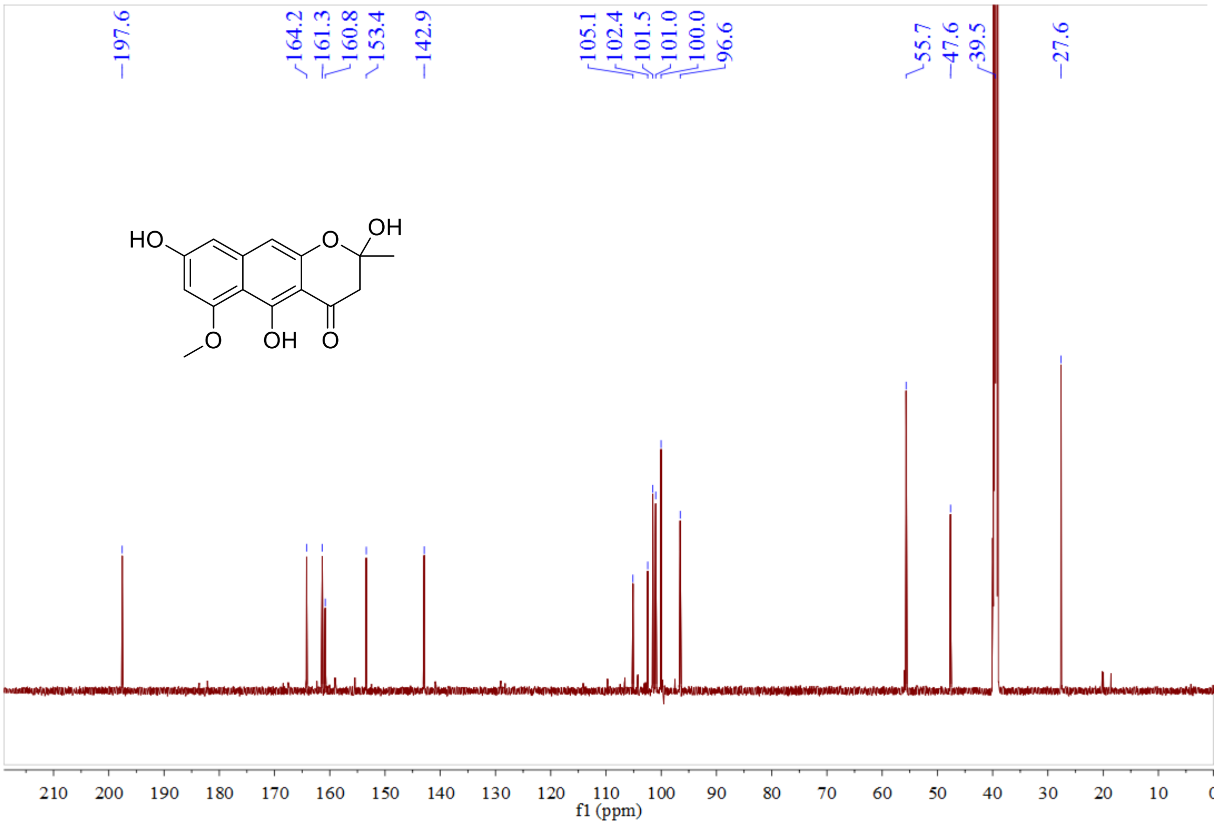

Figure S48. The ^{13}C NMR spectrum (150 MHz) of **14** in DMSO-d_6

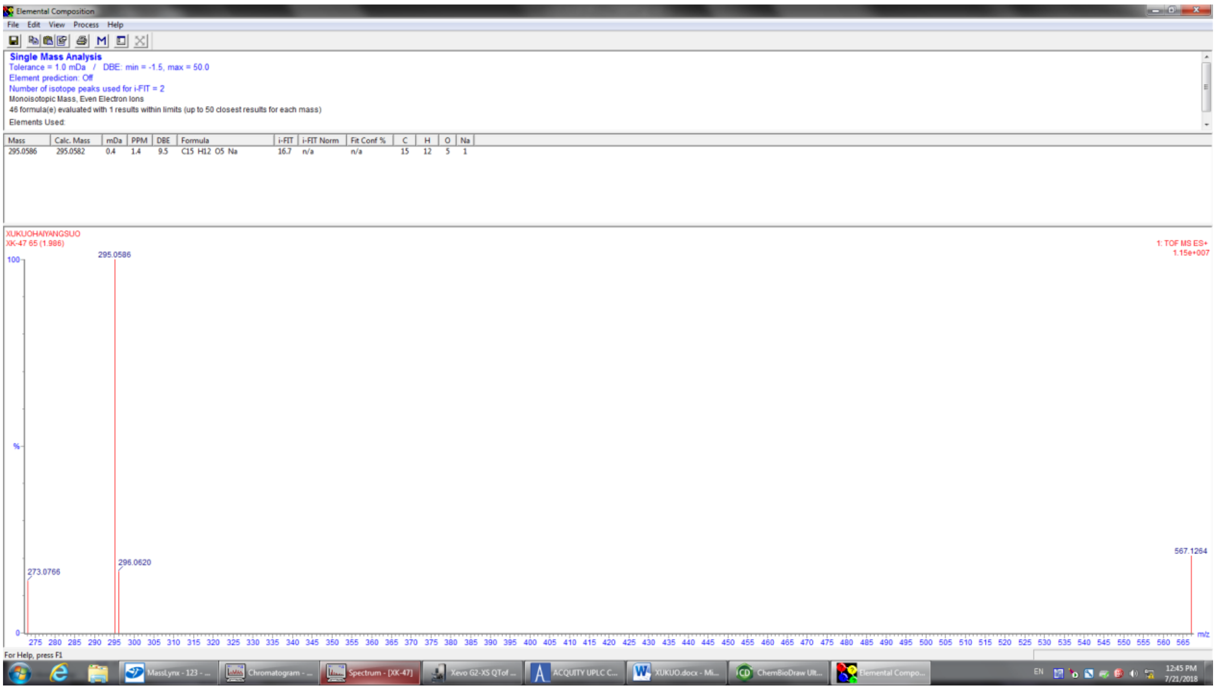

Figure S49. The HR-ESI-MS data of **15**

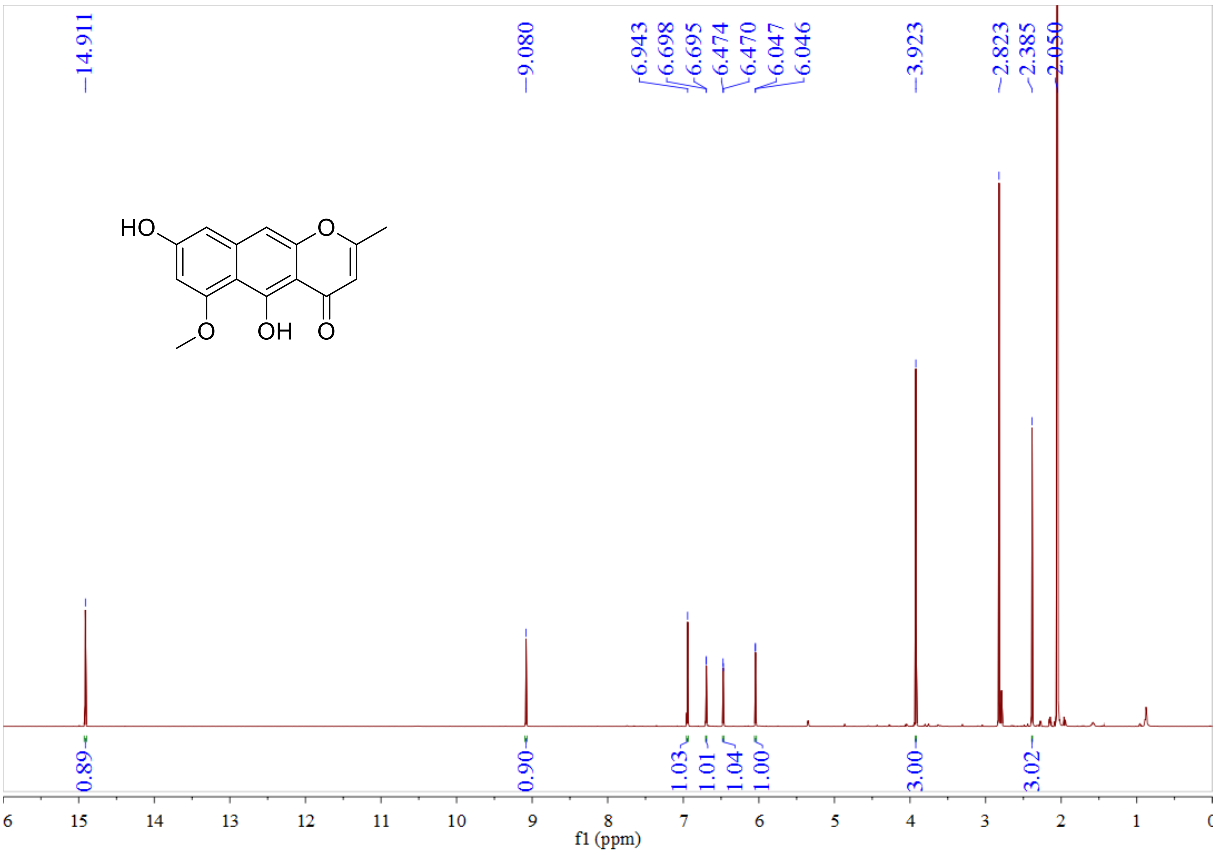

Figure S50. The ^{1}H NMR spectrum (600 MHz) of **15** in acetone-d_6

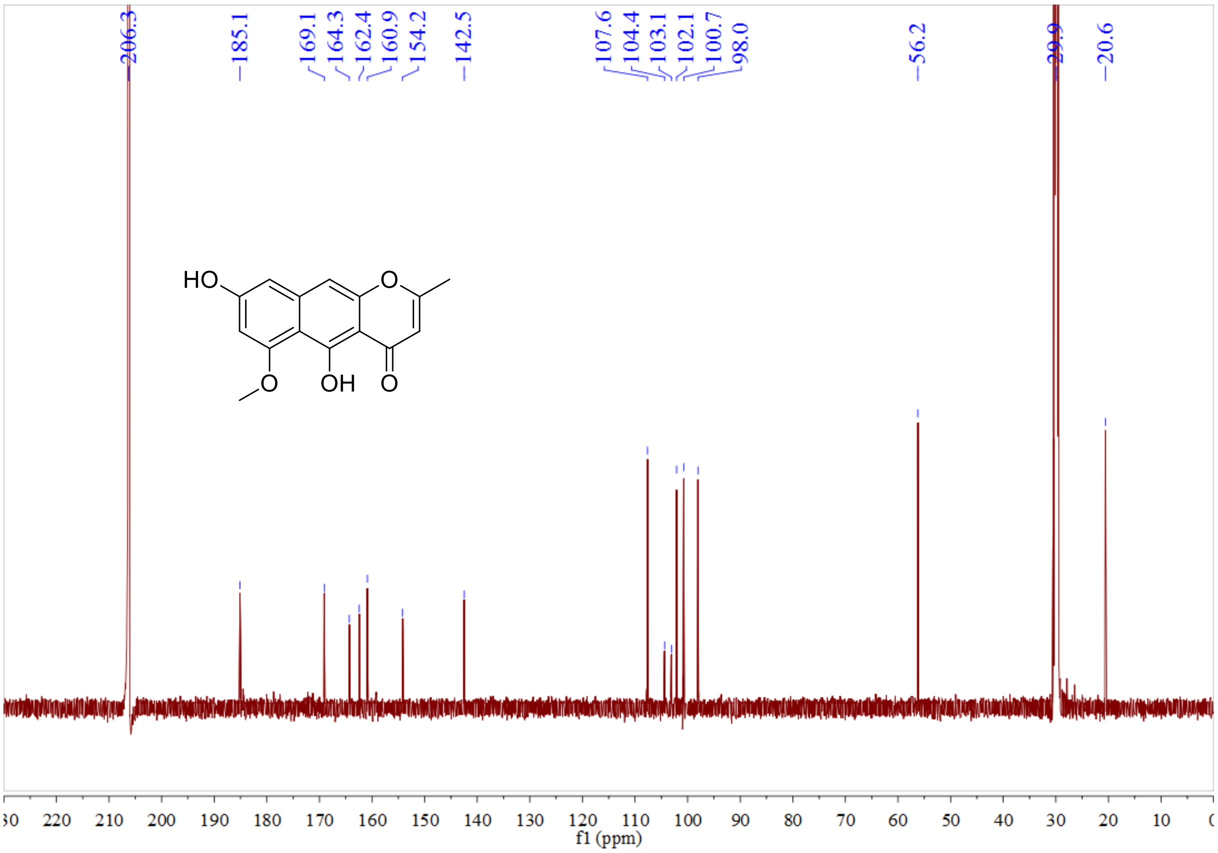

Figure S51. The ^{13}C NMR spectrum (150 MHz) of **15** in acetone-d_6

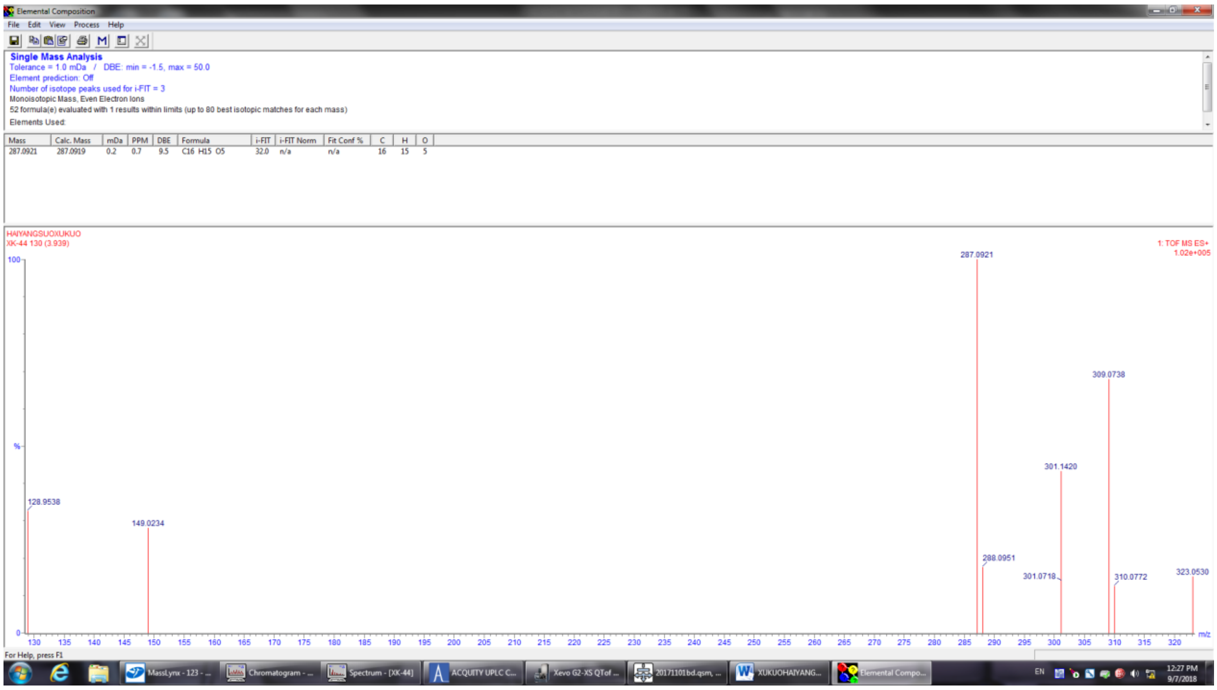

Figure S52. The HR-ESI-MS data of **16**

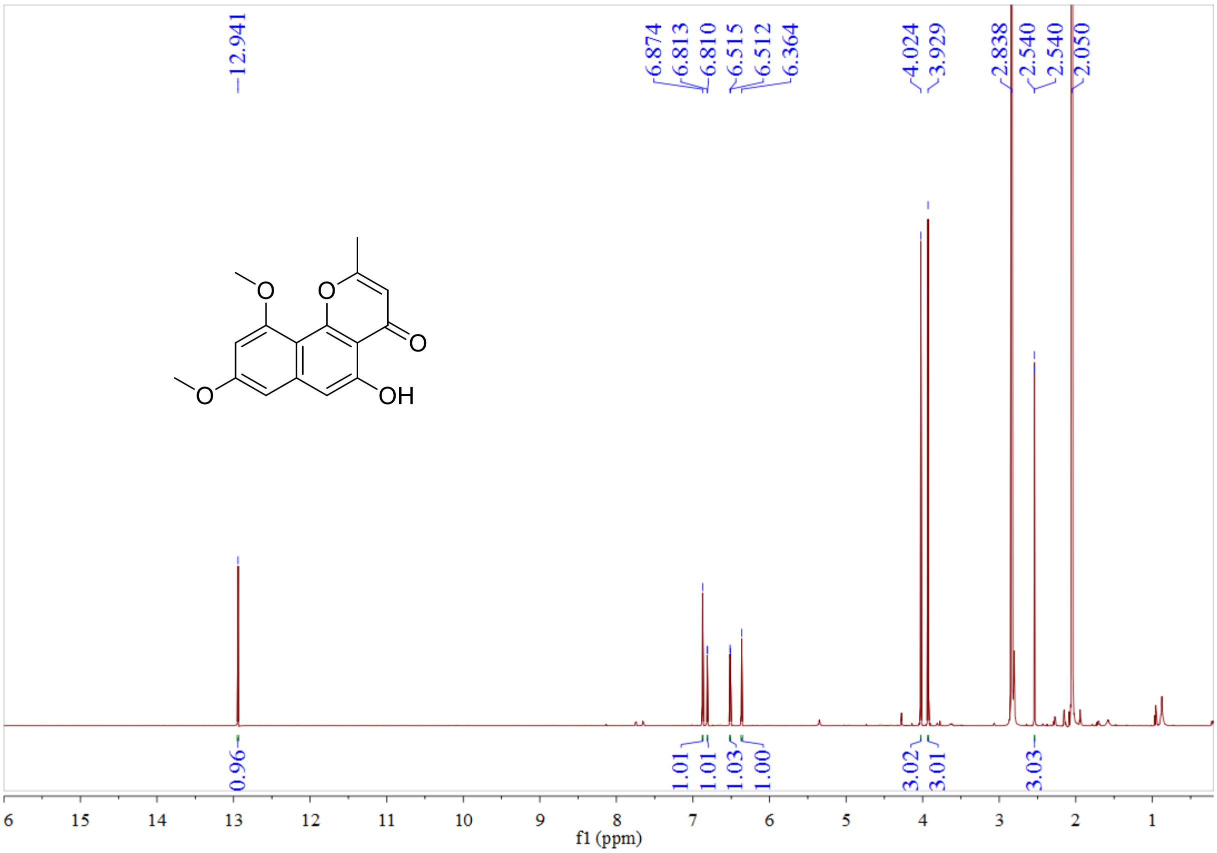

Figure S53. The ^{1}H NMR spectrum (600 MHz) of **16** in acetone-d_6

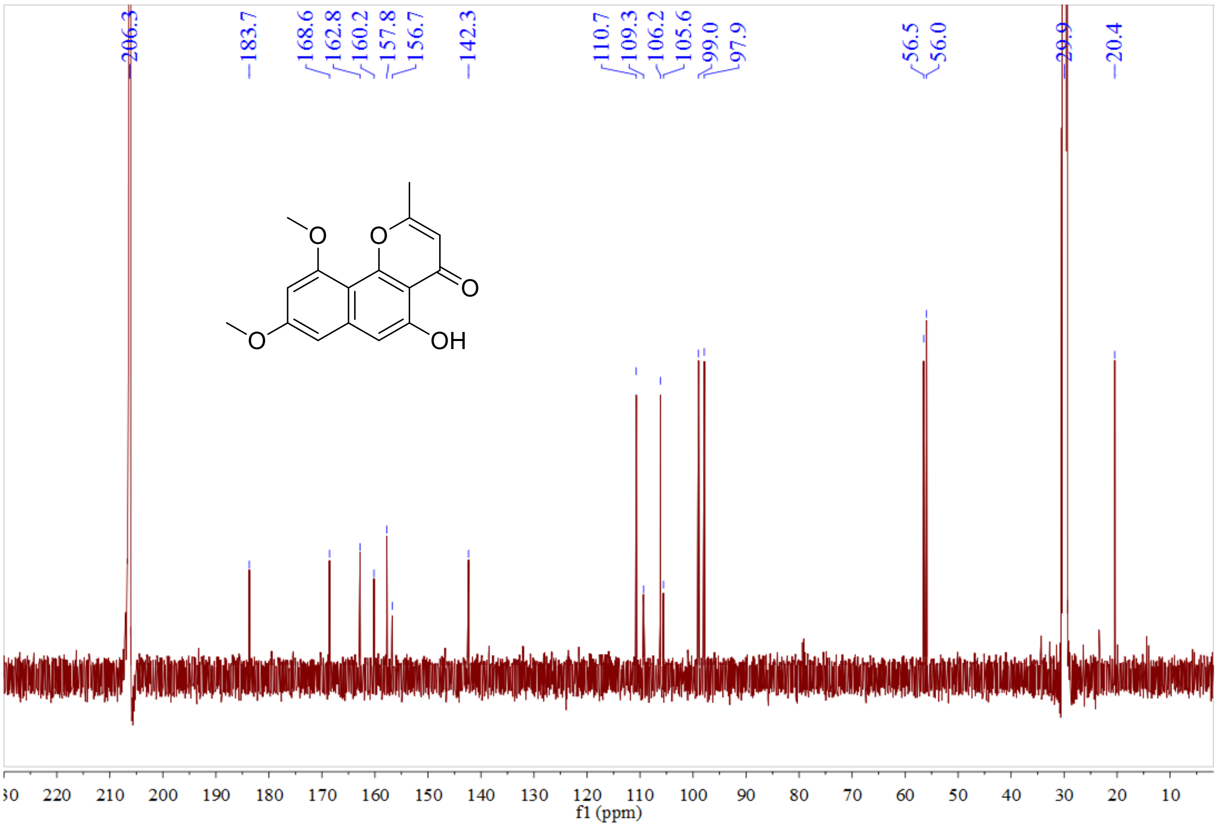

Figure S54. The ^{13}C NMR spectrum (150 MHz) of **16** in acetone-d_6

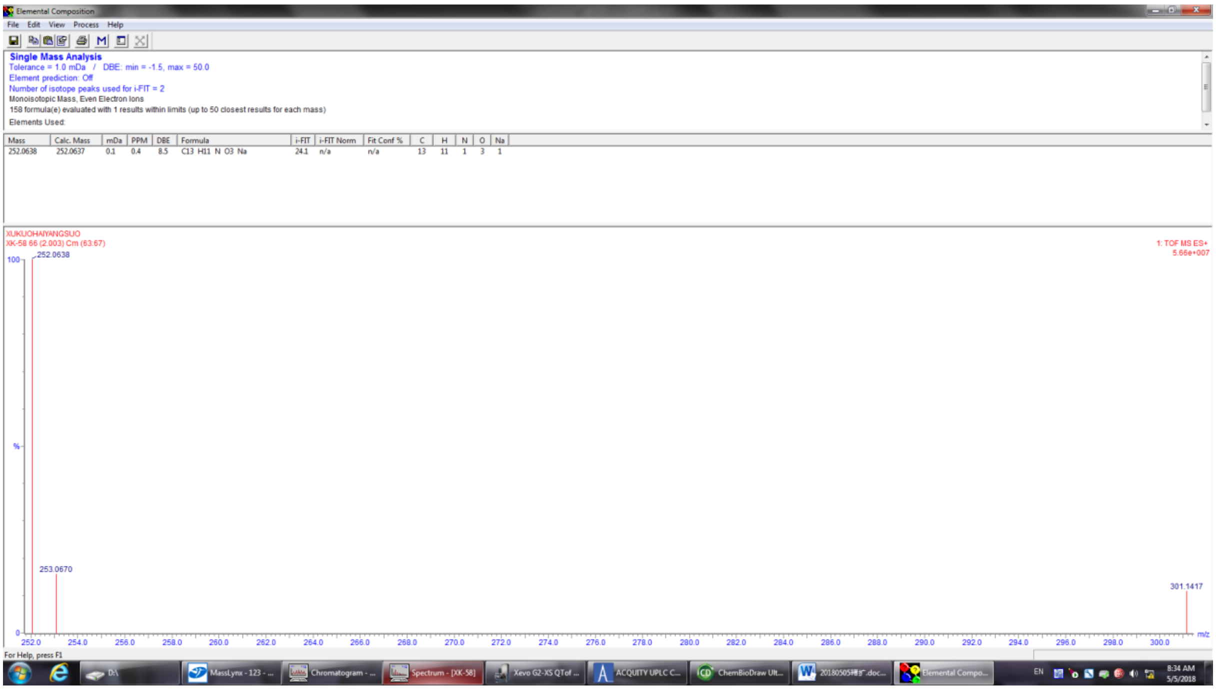

Figure S55. The HR-ESI-MS data of **17**

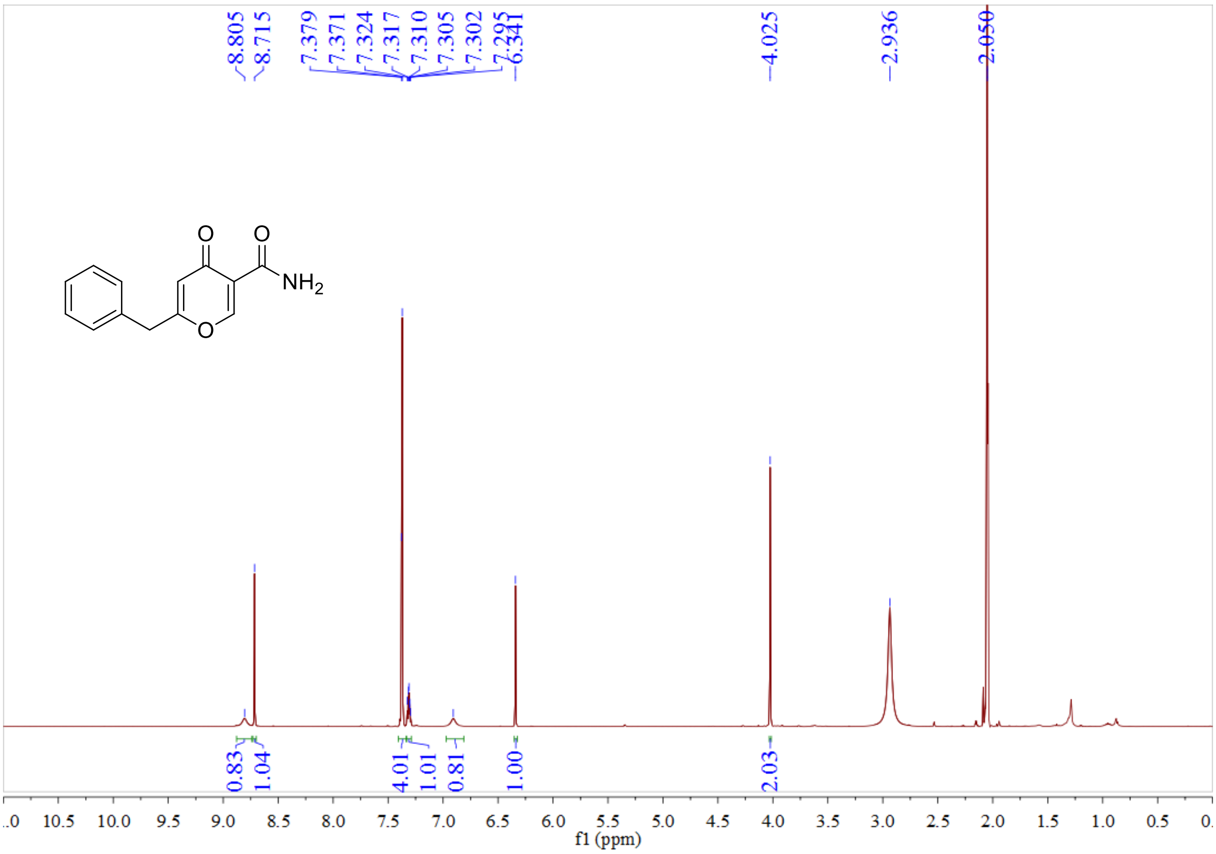

Figure S56. The ^{1}H NMR spectrum (600 MHz) of **17** in acetone-d_6

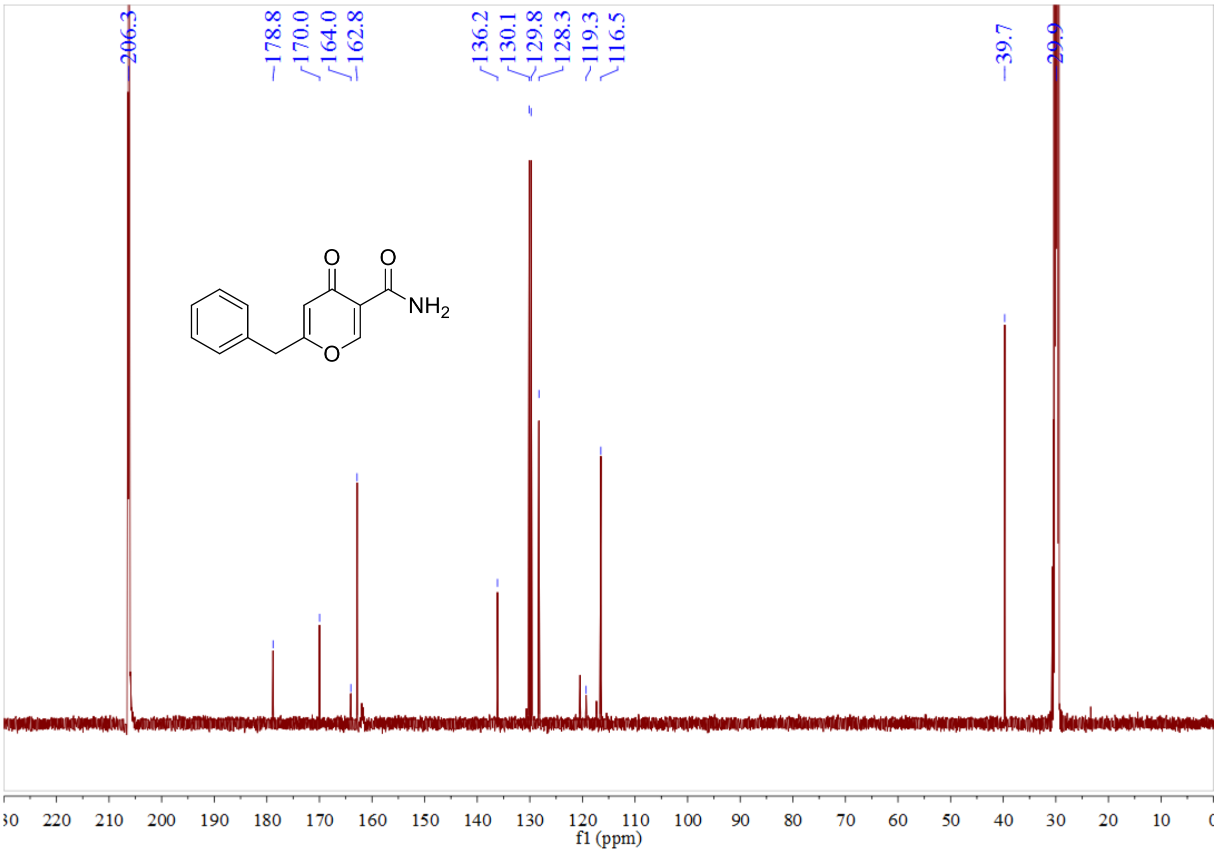

Figure S57. The ^{13}C NMR spectrum (150 MHz) of **17** in acetone-d_6

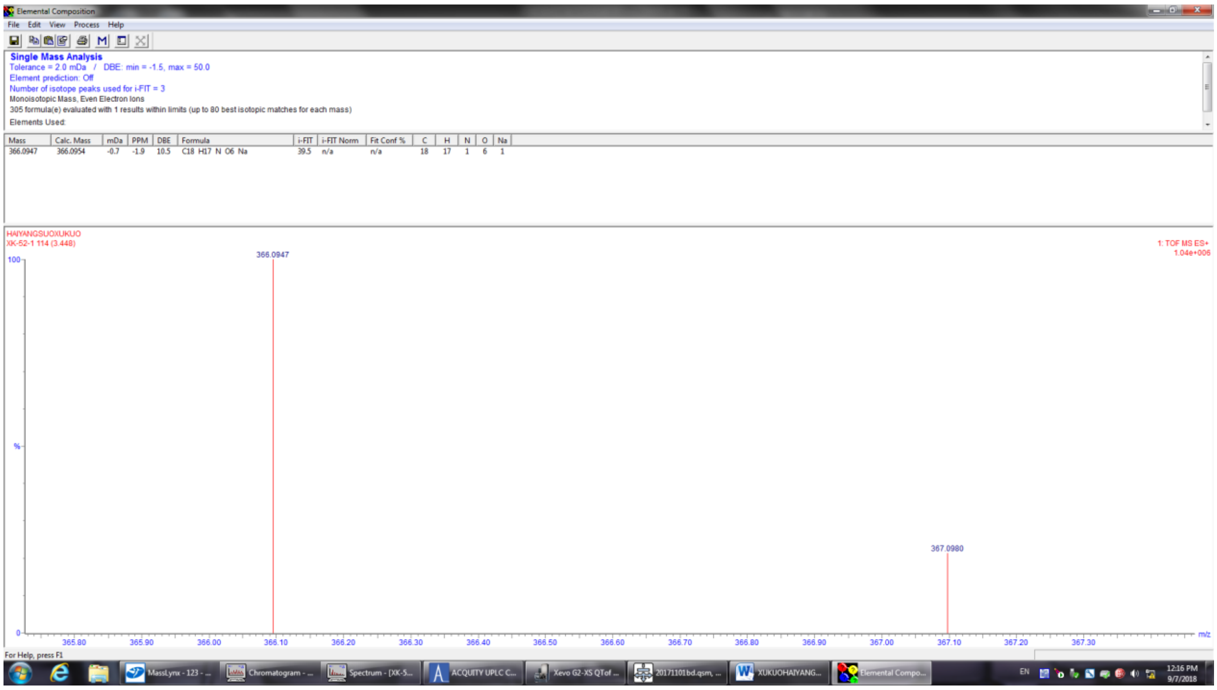

Figure S58. The HR-ESI-MS data of **18**

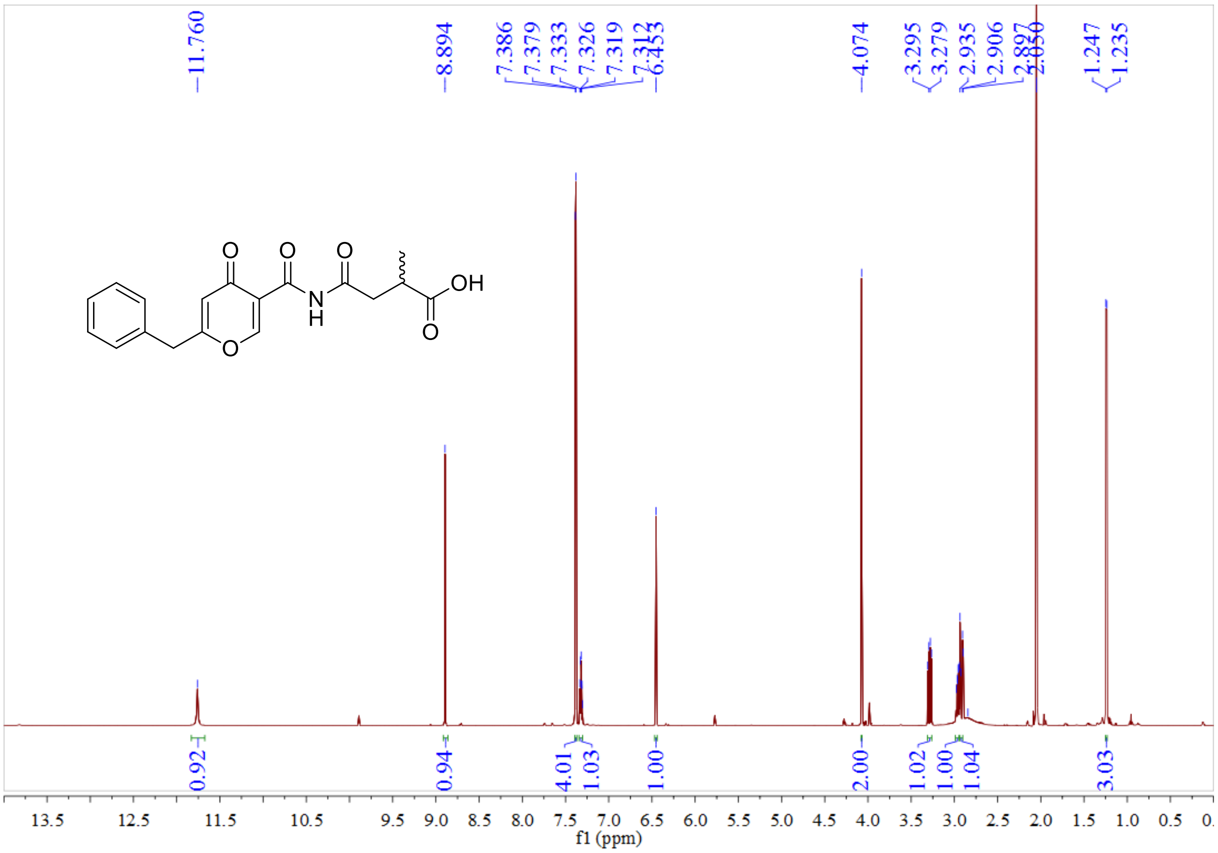

Figure S59. The ^{1}H NMR spectrum (600 MHz) of **18** in acetone-d_6

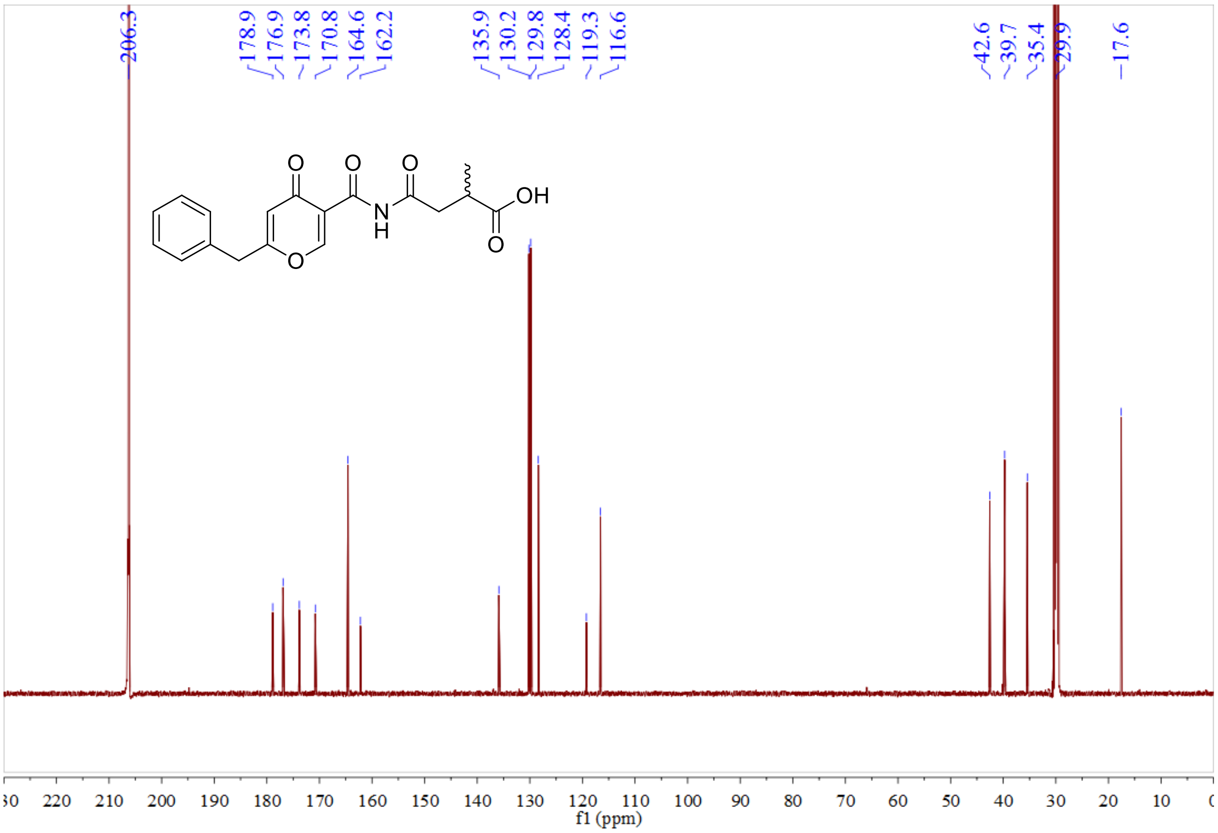

Figure S60. The ^{13}C NMR spectrum (150 MHz) of **18** in acetone-d_6

General procedure for ECD calculations

In general, conformational analyses of compounds **1** and **12** are carried out via systematic searching in the WaveFunction Spartan 14 (version 1.1.4) using the MMFF94 force field. Conformers with Boltzmann distribution over 1% are chosen as the beginning for ECD calculations. Ground-state geometries are optimized at the B3LYP/6-311+G(d,p) level in gas phase by the Gaussian 16 program (Gaussian Inc., Wallingford, CT, USA).[1] All quantum computations are performed on an IBM cluster machine located at the High Performance Computing Center of Peking Union Medical College. The energies, oscillator strengths, and rotational strengths (velocity) of the first 50 electronic excitations are calculated using the TD-DFT methodology at the B3LYP/6-311+G(d,p) level in methanol. The ECD spectra are simulated by the overlapping Gaussian function (half the bandwidth at 1/e peak height, 0.25 eV).[2] By comparison of the calculated and experimental ECD spectra, the absolute configurations of these compounds are established.

Table 1. The 3D conformers of **1** and **12** with Boltzmann distribution over 1%.

**Compounds**	**no.**	**3D Conformers**	**ΔG (kcal/mol)**	**Population (%)**
**1**	1	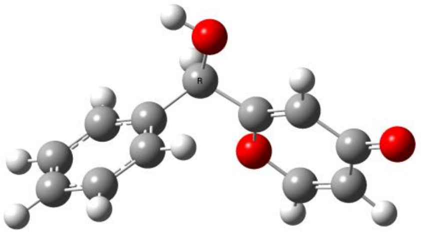	0.00	79.7
	2	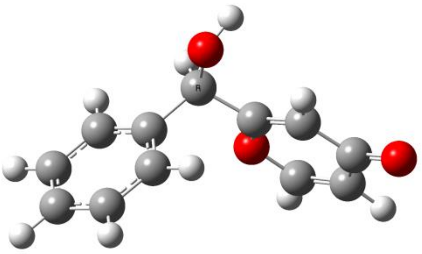	5.92	7.3

	3	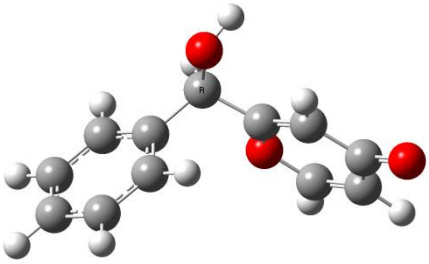	6.73	5.3
	4	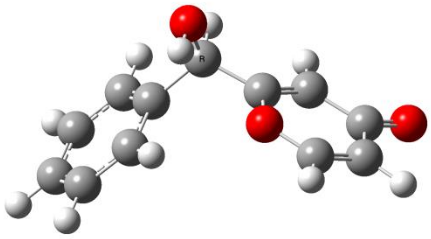	7.48	3.9
	5	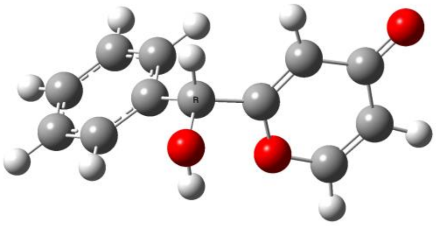	8.39	0.027
	6	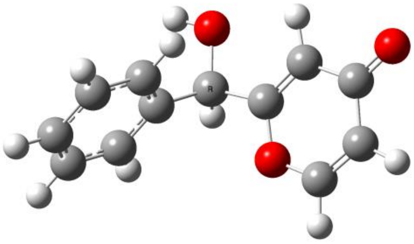	10.54	1.1
**12**	1	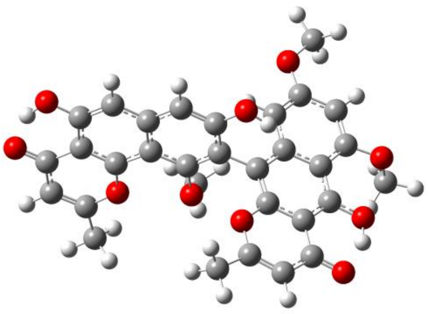	0.00	100.0

References:

1. Frisch, M. J.; Trucks, G. W.; Schlegel, H. B.; Scuseria, G. E.; Robb, M. A.; Cheeseman, J. R.; Scalmani, G.; Barone, V.; Mennucci, B.; Petersson, G. A.; Nakatsuji, H.; Caricato, M.; Li, X.; Hratchian, H. P.; Izmaylov, A. F.; Bloino, J.; Zheng, G.; Sonnenberg, J. L.; Hada, M.; Ehara, M.; Toyota, K.; Fukuda, R.; Hasegawa, J.; Ishida, M.; Nakajima, T.; Honda, Y.; Kitao, O.; Nakai, H.; Vreven, T.; Montgomery, Jr., J. A.; Peralta, J. E.; Ogliaro, F.; Bearpark, M.; Heyd, J. J.; Brothers, E.; Kudin, K. N.; Staroverov, V. N.; Kobayashi, R.; Normand, J.; Raghavachari, K.; Rendell, A.; Burant, J. C.; Iyengar, S. S.; Tomasi, J.; Cossi, M.; Rega, N.; Millam, J. M.; Klene, M.; Knox, J. E.; Cross, J. B.; Bakken, V.; Adamo, C.; Jaramillo, J.; Gomperts, R.; Stratmann, R. E.; Yazyev, O.; Austin, A. J.; Cammi, R.; Pomelli, C.; Ochterski, J. W.; Martin, R. L.; Morokuma, K.; Zakrzewski, V. G.; Voth, G. A.; Salvador, P.; Dannenberg, J. J.; Dapprich, S.; Daniels, A. D.; Farkas, Ö.; Foresman, J. B.; Ortiz, J. V.; Cioslowski, J.; Fox, D. J. Gaussian 09, Rev. C 01; Gaussian, Inc., Wallingford CT, 2009.
2. Stephens, P. J.; Harada, N. ECD cotton effect approximated by the Gaussian curve and other methods. *Chirality* **2010**, *22*, 229–233.

Analytical HPLC spectra for all isolated compounds (1−18)

==== Shimadzu LCsolution 分析报告 ====

样品信息

采集人	: Admin
样品名称	: Compound 1
样品 ID	:
样品架#	: 1
样品瓶#	: 46
进样体积	: 30 uL
数据文件名	: Compound 1(30%乙腈-TD).lcd
方法文件名	: XK-30%-乙腈-TD.lcm
批处理文件名	: XUKUO.lcb
报告文件名	: XUKUO.lcr
数据采集	: 2019-3-6 17:19:48
数据处理	: 2019-3-7 08:26:49

色谱图

Compound 1 E:\HPLC\绪扩\Compound 1(30%乙腈-TD).lcd

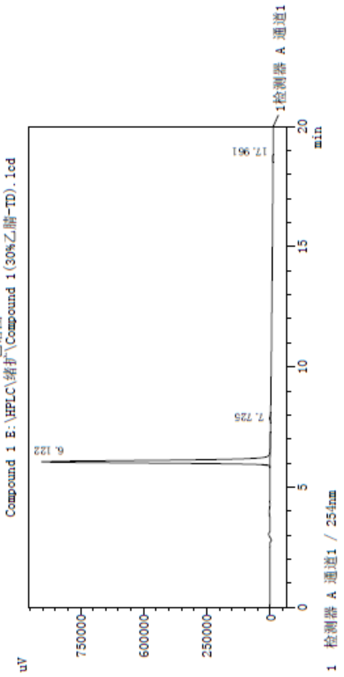

1 检测器 A 通道1 / 254nm

峰表

检测器 A Ch1 254nm

峰#	保留时间	面积	高度	面积 %	高度 %
1	6.122	6871534	791849	99.178	99.272
2	7.725	52315	4817	0.755	0.604
3	17.961	4613	989	0.067	0.124
总计		6928462	797655	100.000	100.000

==== Shimadzu LCsolution 分析报告 ====

样品信息

采集人	: Admin
样品名称	: Compound 1
样品 ID	:
样品架#	: 1
样品瓶#	: 46
进样体积	: 30 uL
数据文件名	: Compound 1(60%甲醇-TD).lcd
方法文件名	: XK-60%-甲醇-TD.lcm
批处理文件名	: XUKUO.lcb
报告文件名	: XUKUO.lcr
数据采集	: 2019-3-6 12:44:53
数据处理	: 2019-3-6 13:21:00

色谱图

Compound 1 E:\HPLC\绪扩\Compound 1(60%甲醇-TD).lcd

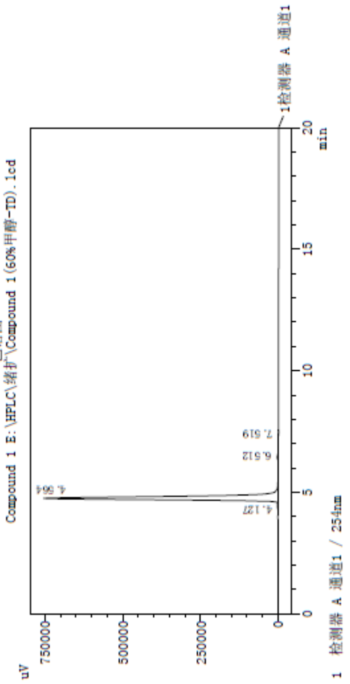

1 检测器 A 通道1 / 254nm

峰表

检测器 A Ch1 254nm

峰#	保留时间	面积	高度	面积 %	高度 %
1	4.127	29911	3217	0.427	0.420
2	4.564	6924155	759430	98.947	98.993
3	6.512	41351	3547	0.591	0.462
4	7.519	2452	961	0.035	0.125
总计		6997869	767155	100.000	100.000

==== Shimadzu LCsolution 分析报告 ====

样品信息

采集人	: Admin
样品名称	: Compound 2
样品 ID	:
样品架#	: 1
样品瓶#	: 47
进样体积	: 60 uL
数据文件名	: Compound 2(60%甲醇-TD).lcd
方法文件名	: XK-60%-甲醇-TD.lcm
批处理文件名	: XUKUO.lcb
报告文件名	: XUKUO.lcr
数据采集	: 2019-3-6 13:08:24
数据处理	: 2019-3-6 13:38:44

色谱图
Compound 2 E:\HPLC\绪扩\Compound 2(60%甲醇-TD).lcd

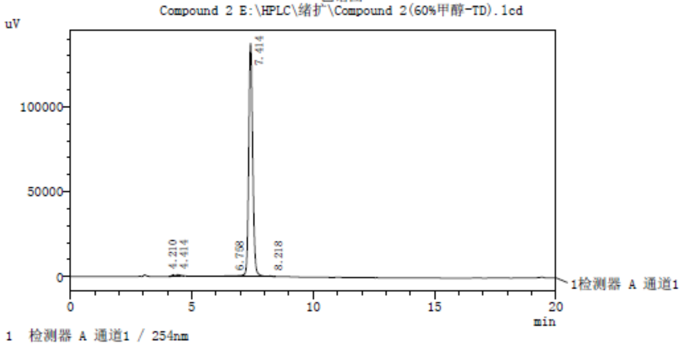

1 检测器 A 通道1 / 254nm

峰表

检测器 A Ch1 254nm

峰#	保留时间	面积	高度	面积 %	高度 %
1	4.210	7135	966	0.445	0.691
2	4.414	10149	938	0.633	0.672
3	6.758	7520	288	0.469	0.206
4	7.414	1575501	137290	98.313	98.258
5	8.218	2227	242	0.139	0.173
总计		1602532	139724	100.000	100.000

==== Shimadzu LCsolution 分析报告 ====

样品信息

采集人	: Admin
样品名称	: Compound 2
样品 ID	:
样品架#	: 1
样品瓶#	: 47
进样体积	: 60 uL
数据文件名	: Compound 2(30%乙腈-TD).lcd
方法文件名	: XK-30%-乙腈-TD.lcm
批处理文件名	: XUKUO.lcb
报告文件名	: XUKUO.lcr
数据采集	: 2019-3-6 17:43:17
数据处理	: 2019-3-7 08:27:38

色谱图
Compound 2 E:\HPLC\绪扩\Compound 2(30%乙腈-TD).lcd

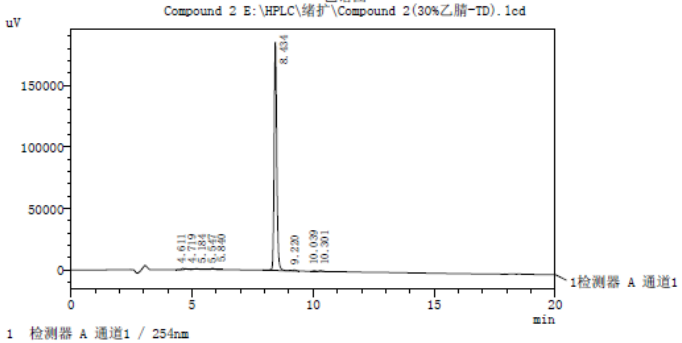

1 检测器 A 通道1 / 254nm

峰表

检测器 A Ch1 254nm

峰#	保留时间	面积	高度	面积 %	高度 %
1	4.611	6428	970	0.444	0.506
2	4.719	7637	824	0.527	0.430
3	5.184	10663	803	0.736	0.419
4	5.547	4283	329	0.296	0.172
5	5.840	10350	1115	0.715	0.582
6	8.434	1393574	185416	96.240	96.782
7	9.220	5936	797	0.410	0.416
8	10.039	4310	642	0.298	0.335
9	10.301	4842	684	0.334	0.357
总计		1448022	191580	100.000	100.000

==== Shimadzu LCsolution 分析报告 ====

样品信息

采集人	: Admin
样品名称	: Compound 3
样品 ID	:
样品架#	: 1
样品瓶#	: 48
进样体积	: 30 uL
数据文件名	: Compound 3(60%甲醇-TD).lcd
方法文件名	: XK-60%-甲醇-TD.lcm
批处理文件名	: XUKUO.lcb
报告文件名	: XUKUO.lcr
数据采集	: 2019-3-6 13:31:50
数据处理	: 2019-3-6 14:03:07

色谱图

Compound 3 E:\HPLC\绪扩\Compound 3(60%甲醇-TD).lcd

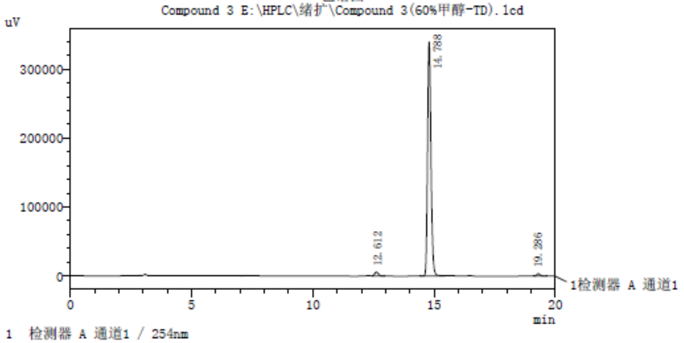

1 检测器 A 通道1 / 254nm

峰表

检测器 A Ch1 254nm

峰#	保留时间	面积	高度	面积 %	高度 %
1	12.612	59867	5609	1.762	1.603
2	14.788	3302297	340396	97.171	97.270
3	19.286	36264	3946	1.067	1.128
总计		3398429	349951	100.000	100.000

==== Shimadzu LCsolution 分析报告 ====

样品信息

采集人	: Admin
样品名称	: Compound 3
样品 ID	:
样品架#	: 1
样品瓶#	: 48
进样体积	: 30 uL
数据文件名	: Compound 3(30%乙腈-TD).lcd
方法文件名	: XK-30%-乙腈-TD.lcm
批处理文件名	: XUKUO.lcb
报告文件名	: XUKUO.lcr
数据采集	: 2019-3-6 18:06:46
数据处理	: 2019-3-7 08:06:40

色谱图

Compound 3 E:\HPLC\绪扩\Compound 3(30%乙腈-TD).lcd

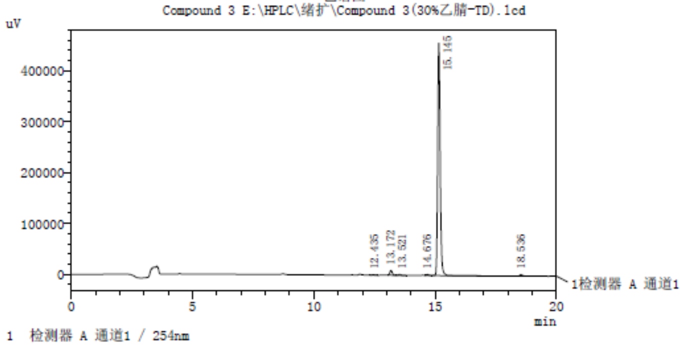

1 检测器 A 通道1 / 254nm

峰表

检测器 A Ch1 254nm

峰#	保留时间	面积	高度	面积 %	高度 %
1	12.435	6071	851	0.179	0.180
2	13.172	67739	9412	2.001	1.986
3	13.521	14026	1862	0.414	0.393
4	14.676	11380	1641	0.336	0.346
5	15.145	3263820	457110	96.433	96.471
6	18.536	21495	2954	0.635	0.623
总计		3384530	473830	100.000	100.000

==== Shimadzu LCsolution 分析报告 ====

样品信息

采集人	: Admin
样品名称	: Compound 4
样品 ID	:
样品架#	: 1
样品瓶#	: 49
进样体积	: 30 uL
数据文件名	: Compound 4(60%甲醇-TD).lcd
方法文件名	: XK-60%-甲醇-TD.lcm
批处理文件名	: XUKUO.lcb
报告文件名	: XUKUO.lcr
数据采集	: 2019-3-6 13:55:16
数据处理	: 2019-3-6 14:25:16

色谱图

Compound 4 E:\HPLC\绪扩\Compound 4(60%甲醇-TD).lcd

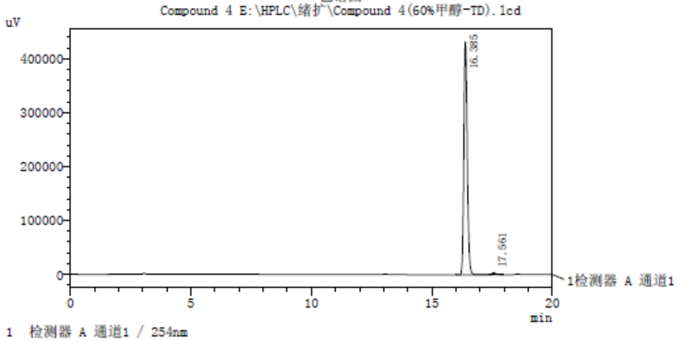

1 检测器 A 通道1 / 254nm

峰表

检测器 A Ch1 254nm

峰#	保留时间	面积	高度	面积 %	高度 %
1	16.385	4229896	432281	99.178	99.183
2	17.561	35045	3559	0.822	0.817
总计		4264941	435840	100.000	100.000

==== Shimadzu LCsolution 分析报告 ====

样品信息

采集人	: Admin
样品名称	: Compound 4
样品 ID	:
样品架#	: 1
样品瓶#	: 49
进样体积	: 30 uL
数据文件名	: Compound 4(30%乙腈-TD).lcd
方法文件名	: XK-30%-乙腈-TD.lcm
批处理文件名	: XUKUO.lcb
报告文件名	: XUKUO.lcr
数据采集	: 2019-3-6 18:30:13
数据处理	: 2019-3-7 07:53:23

色谱图

Compound 4 E:\HPLC\绪扩\Compound 4(30%乙腈-TD).lcd

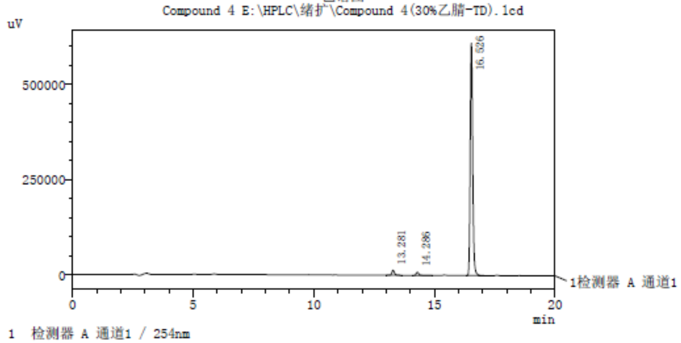

1 检测器 A 通道1 / 254nm

峰表

检测器 A Ch1 254nm

峰#	保留时间	面积	高度	面积 %	高度 %
1	13.281	96086	13107	2.120	2.067
2	14.286	67584	9168	1.491	1.446
3	16.526	4367643	611821	96.388	96.487
总计		4531314	634096	100.000	100.000

==== Shimadzu LCsolution 分析报告 ====

样品信息

采集人	:	Admin
样品名称	:	Compound 5
样品 ID	:	
样品架#	:	1
样品瓶#	:	50
进样体积	:	30 uL
数据文件名	:	Compound 5(60%甲醇-TD).lcd
方法文件名	:	XK-60%-甲醇-TD.lcm
批处理文件名	:	XUKUO.lcb
报告文件名	:	XUKUO.lcr
数据采集	:	2019-3-6 14:18:44
数据处理	:	2019-3-6 14:48:35

色谱图

Compound 5 E:\HPLC\绪扩\Compound 5(60%甲醇-TD).lcd

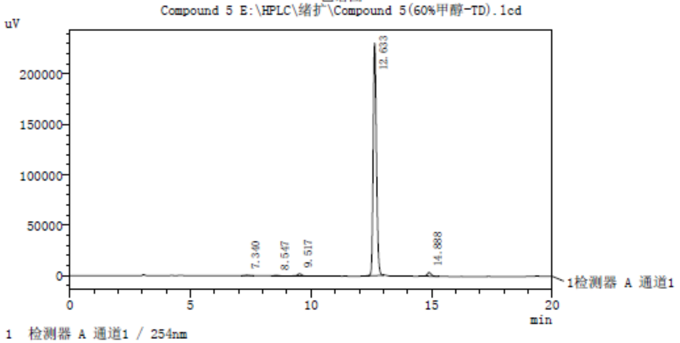

峰表

检测器 A Ch1 254nm

峰#	保留时间	面积	高度	面积 %	高度 %
1	7.340	5501	505	0.244	0.212
2	8.547	7281	643	0.323	0.270
3	9.517	29414	2316	1.305	0.973
4	12.633	2171535	230730	96.369	96.978
5	14.888	39612	3727	1.758	1.566
总计		2253343	237920	100.000	100.000

==== Shimadzu LCsolution 分析报告 ====

样品信息

采集人	:	Admin
样品名称	:	Compound 5
样品 ID	:	
样品架#	:	1
样品瓶#	:	50
进样体积	:	30 uL
数据文件名	:	Compound 5(30%乙腈-TD).lcd
方法文件名	:	XK-30%-乙腈-TD.lcm
批处理文件名	:	XUKUO.lcb
报告文件名	:	XUKUO.lcr
数据采集	:	2019-3-6 18:53:38
数据处理	:	2019-3-7 07:54:32

色谱图

Compound 5 E:\HPLC\绪扩\Compound 5(30%乙腈-TD).lcd

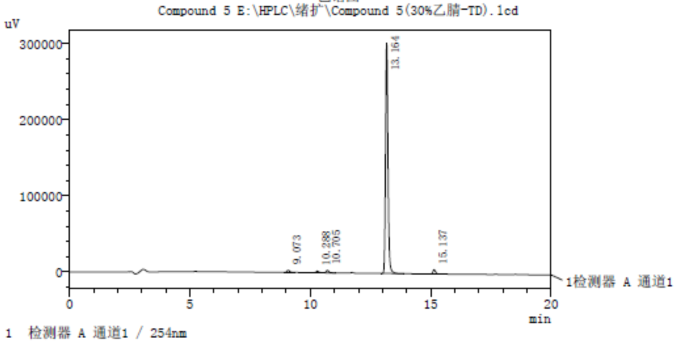

峰表

检测器 A Ch1 254nm

峰#	保留时间	面积	高度	面积 %	高度 %
1	9.073	23507	3022	1.047	0.954
2	10.288	18090	2172	0.806	0.686
3	10.705	24309	3315	1.083	1.047
4	13.164	2137994	302540	95.253	95.526
5	15.137	40655	5662	1.811	1.788
总计		2244553	316711	100.000	100.000

==== Shimadzu LCsolution 分析报告 ====

样品信息

采集人	: Admin
样品名称	: Compound 6
样品 ID	:
样品架#	: 1
样品瓶#	: 51
进样体积	: 30 uL
数据文件名	: Compound 6(60%甲醇-TD).lcd
方法文件名	: XK-60%-甲醇-TD.lcm
批处理文件名	: XUKUO.lcb
报告文件名	: XUKUO.lcr
数据采集	: 2019-3-6 14:42:10
数据处理	: 2019-3-6 15:05:12

色谱图

Compound 6 E:\HPLC\绪扩\Compound 6(60%甲醇-TD).lcd

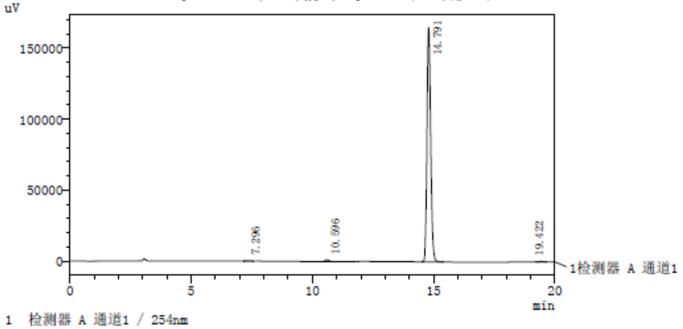

1 检测器 A 通道1 / 254nm

峰表

检测器 A Ch1 254nm

峰#	保留时间	面积	高度	面积 %	高度 %
1	7.296	1318	133	0.074	0.079
2	10.596	15553	1412	0.876	0.845
3	14.791	1755165	165045	98.809	98.787
4	19.422	4280	483	0.241	0.289
总计		1776317	167072	100.000	100.000

==== Shimadzu LCsolution 分析报告 ====

样品信息

采集人	: Admin
样品名称	: Compound 6
样品 ID	:
样品架#	: 1
样品瓶#	: 51
进样体积	: 30 uL
数据文件名	: Compound 6(30%乙腈-TD).lcd
方法文件名	: XK-30%-乙腈-TD.lcm
批处理文件名	: XUKUO.lcb
报告文件名	: XUKUO.lcr
数据采集	: 2019-3-6 19:17:04
数据处理	: 2019-3-6 19:40:05

色谱图

Compound 6 E:\HPLC\绪扩\Compound 6(30%乙腈-TD).lcd

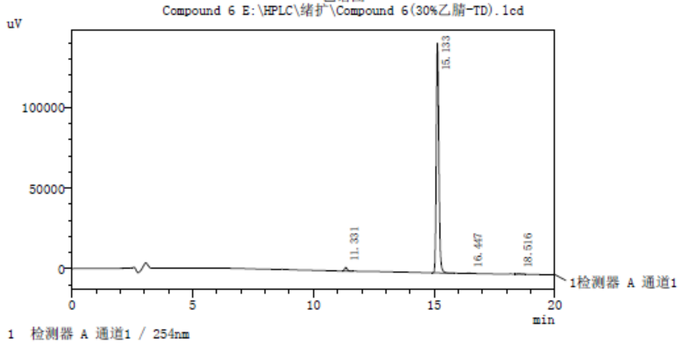

1 检测器 A 通道1 / 254nm

峰表

检测器 A Ch1 254nm

峰#	保留时间	面积	高度	面积 %	高度 %
1	11.331	16413	2257	1.507	1.552
2	15.133	1066852	142534	97.936	98.005
3	16.447	3299	345	0.303	0.237
4	18.516	2769	300	0.254	0.206
总计		1089334	145436	100.000	100.000

==== Shimadzu LCsolution 分析报告 ====

样品信息

采集人	:	Admin
样品名称	:	Compound 7
样品 ID	:	
样品架#	:	1
样品瓶#	:	52
进样体积	:	30 uL
数据文件名	:	Compound 7(60%甲醇-TD).lcd
方法文件名	:	XK-60%-甲醇-TD.lcm
批处理文件名	:	XUKU0.lcb
报告文件名	:	XUKU0.lcr
数据采集	:	2019-3-6 15:05:37
数据处理	:	2019-3-6 15:43:01

色谱图

Compound 7 E:\HPLC\绪扩\Compound 7(60%甲醇-TD).lcd

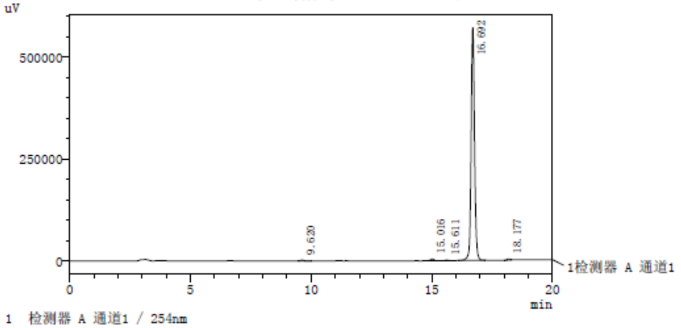

1 检测器 A 通道1 / 254nm

峰表

检测器 A Ch1 254nm

峰#	保留时间	面积	高度	面积 %	高度 %
1	9.620	17993	2083	0.318	0.358
2	15.016	40689	4152	0.719	0.714
3	15.611	16362	1749	0.289	0.301
4	16.692	5556462	570349	98.233	98.128
5	18.177	24929	2894	0.441	0.498
总计		5656435	581227	100.000	100.000

==== Shimadzu LCsolution 分析报告 ====

样品信息

采集人	:	Admin
样品名称	:	Compound 7
样品 ID	:	
样品架#	:	1
样品瓶#	:	52
进样体积	:	30 uL
数据文件名	:	Compound 7(30%乙腈-TD).lcd
方法文件名	:	XK-30%-乙腈-TD.lcm
批处理文件名	:	XUKU0.lcb
报告文件名	:	XUKU0.lcr
数据采集	:	2019-3-6 19:40:31
数据处理	:	2019-3-7 07:56:14

色谱图

Compound 7 E:\HPLC\绪扩\Compound 7(30%乙腈-TD).lcd

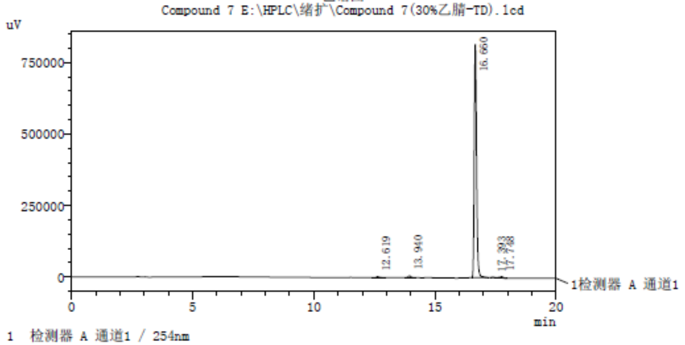

1 检测器 A 通道1 / 254nm

峰表

检测器 A Ch1 254nm

峰#	保留时间	面积	高度	面积 %	高度 %
1	12.619	38070	4149	0.691	0.498
2	13.940	48363	6442	0.878	0.773
3	16.660	5357551	816111	97.240	97.912
4	17.393	16389	1687	0.297	0.202
5	17.748	49242	5122	0.894	0.615
总计		5509615	833511	100.000	100.000

==== Shimadzu LCsolution 分析报告 ====

样品信息

采集人	: Admin
样品名称	: Compound 8
样品 ID	:
样品架#	: 1
样品瓶#	: 53
进样体积	: 30 uL
数据文件名	: Compound 8(60%甲醇-TD).lcd
方法文件名	: XK-60%-甲醇-TD.lcm
批处理文件名	: XUKU0.lcb
报告文件名	: XUKU0.lcr
数据采集	: 2019-3-6 15:29:04
数据处理	: 2019-3-6 15:54:28

色谱图
Compound 8 E:\HPLC\绪扩\Compound 8(60%甲醇-TD).lcd

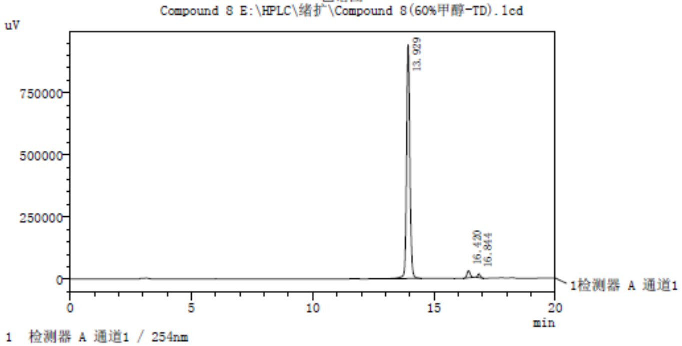

1 检测器 A 通道1 / 254nm

峰表

检测器 A Ch1 254nm

峰#	保留时间	面积	高度	面积 %	高度 %
1	13.929	8975892	940773	96.159	95.550
2	16.420	252002	28261	2.700	2.870
3	16.844	106544	15553	1.141	1.580
总计		9334439	984587	100.000	100.000

==== Shimadzu LCsolution 分析报告 ====

样品信息

采集人	: Admin
样品名称	: Compound 8
样品 ID	:
样品架#	: 1
样品瓶#	: 53
进样体积	: 30 uL
数据文件名	: Compound 8(30%乙腈-TD).lcd
方法文件名	: XK-30%-乙腈-TD.lcm
批处理文件名	: XUKU0.lcb
报告文件名	: XUKU0.lcr
数据采集	: 2019-3-6 20:03:58
数据处理	: 2019-3-7 07:57:34

色谱图
Compound 8 E:\HPLC\绪扩\Compound 8(30%乙腈-TD).lcd

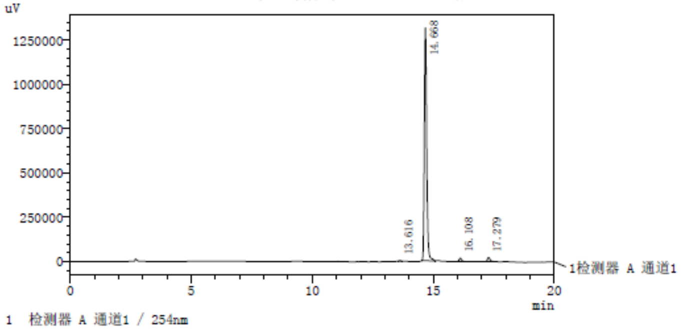

1 检测器 A 通道1 / 254nm

峰表

检测器 A Ch1 254nm

峰#	保留时间	面积	高度	面积 %	高度 %
1	13.616	16816	3636	0.183	0.267
2	14.668	8870059	1314075	96.479	96.608
3	16.108	132943	19362	1.446	1.423
4	17.279	173960	23146	1.892	1.702
总计		9193778	1360219	100.000	100.000

==== Shimadzu LCsolution 分析报告 ====

样品信息

采集人	: Admin
样品名称	: Compound 9
样品 ID	:
样品架#	: 1
样品瓶#	: 54
进样体积	: 30 uL
数据文件名	: Compound 9(60%甲醇-TD).lcd
方法文件名	: XK-60%-甲醇-TD.lcm
批处理文件名	: XUKU0.lcb
报告文件名	: XUKU0.lcr
数据采集	: 2019-3-6 15:52:32
数据处理	: 2019-3-7 07:59:29

色谱图
Compound 9 E:\HPLC\绪扩\Compound 9(60%甲醇-TD).lcd

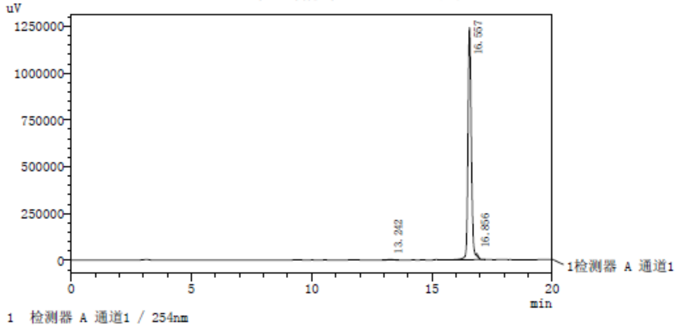

1 检测器 A 通道1 / 254nm

峰表

检测器 A Ch1 254nm

峰#	保留时间	面积	高度	面积 %	高度 %
1	13.242	65800	4391	0.540	0.350
2	16.557	12059614	1240172	99.001	98.786
3	16.856	55928	10845	0.459	0.864
总计		12181342	1255408	100.000	100.000

==== Shimadzu LCsolution 分析报告 ====

样品信息

采集人	: Admin
样品名称	: Compound 9
样品 ID	:
样品架#	: 1
样品瓶#	: 54
进样体积	: 30 uL
数据文件名	: Compound 9(30%乙腈-TD).lcd
方法文件名	: XK-30%-乙腈-TD.lcm
批处理文件名	: XUKU0.lcb
报告文件名	: XUKU0.lcr
数据采集	: 2019-3-6 20:27:25
数据处理	: 2019-3-7 08:01:13

色谱图
Compound 9 E:\HPLC\绪扩\Compound 9(30%乙腈-TD).lcd

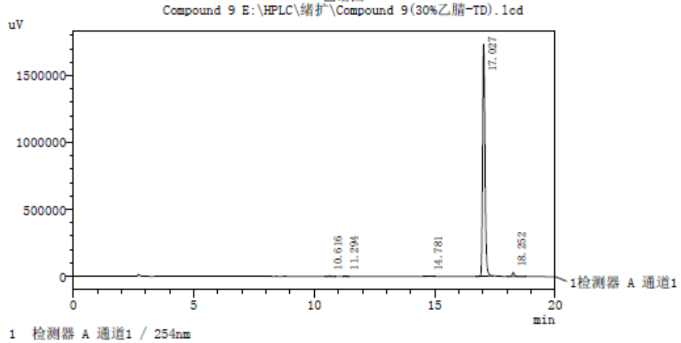

1 检测器 A 通道1 / 254nm

峰表

检测器 A Ch1 254nm

峰#	保留时间	面积	高度	面积 %	高度 %
1	10.616	34533	4487	0.283	0.253
2	11.294	22204	3837	0.182	0.216
3	14.781	44905	2537	0.367	0.143
4	17.027	11921803	1734735	97.547	97.744
5	18.252	198103	29174	1.621	1.644
总计		12221549	1774769	100.000	100.000

==== Shimadzu LCsolution 分析报告 ====

样品信息

采集人	: Admin
样品名称	: Compound 10
样品 ID	:
样品架#	: 1
样品瓶#	: 55
进样体积	: 30 uL
数据文件名	: Compound 10(60%甲醇-TD).lcd
方法文件名	: XK-60%-甲醇-TD.lcm
批处理文件名	: XUKU0.lcb
报告文件名	: XUKU0.lcr
数据采集	: 2019-3-6 16:16:01
数据处理	: 2019-3-7 08:02:54

色谱图

Compound 10 E:\HPLC\绪扩\Compound 10(60%甲醇-TD).lcd

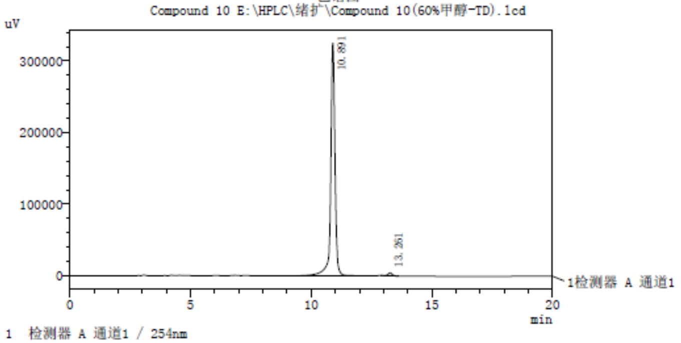

1 检测器 A 通道1 / 254nm

峰表

检测器 A Ch1 254nm

峰#	保留时间	面积	高度	面积 %	高度 %
1	10.891	3961183	324956	98.731	98.715
2	13.261	50900	4230	1.269	1.285
总计		4012083	329185	100.000	100.000

==== Shimadzu LCsolution 分析报告 ====

样品信息

采集人	: Admin
样品名称	: Compound 10
样品 ID	:
样品架#	: 1
样品瓶#	: 55
进样体积	: 30 uL
数据文件名	: Compound 10(30%乙腈-TD).lcd
方法文件名	: XK-30%-乙腈-TD.lcm
批处理文件名	: XUKU0.lcb
报告文件名	: XUKU0.lcr
数据采集	: 2019-3-6 20:50:53
数据处理	: 2019-3-7 08:03:59

色谱图

Compound 10 E:\HPLC\绪扩\Compound 10(30%乙腈-TD).lcd

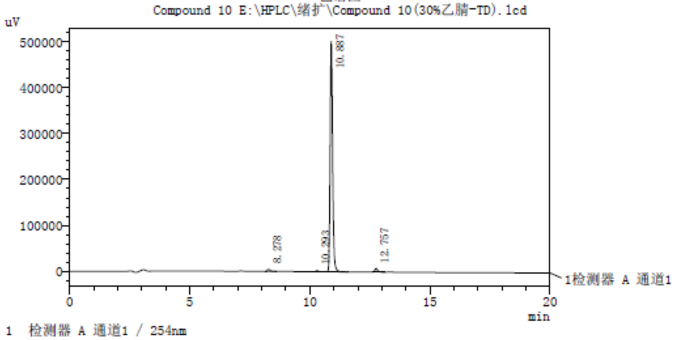

1 检测器 A 通道1 / 254nm

峰表

检测器 A Ch1 254nm

峰#	保留时间	面积	高度	面积 %	高度 %
1	8.278	35839	4680	0.969	0.904
2	10.293	18889	2387	0.510	0.461
3	10.887	3585535	502264	96.899	97.057
4	12.757	60037	8165	1.622	1.578
总计		3700299	517496	100.000	100.000

==== Shimadzu LCsolution 分析报告 ====

样品信息

采集人	:	Admin
样品名称	:	Compound 11
样品 ID	:	
样品架#	:	1
样品瓶#	:	56
进样体积	:	30 uL
数据文件名	:	Compound 11(60%甲醇-TD).lcd
方法文件名	:	XK-60%-甲醇-TD.lcm
批处理文件名	:	XUKUO.lcb
报告文件名	:	XUKUO.lcr
数据采集	:	2019-3-7 13:16:41
数据处理	:	2019-3-7 13:39:43

色谱图
Compound 11 E:\HPLC\绪扩\Compound 11(60%甲醇-TD).lcd

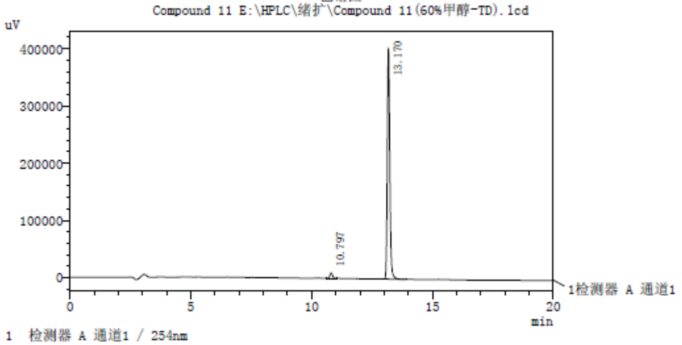

1 检测器 A 通道1 / 254nm

峰表

检测器 A Ch1 254nm

峰#	保留时间	面积	高度	面积 %	高度 %
1	10.797	184367	11575	2.556	2.767
2	13.170	7029857	406754	97.444	97.233
总计		7214224	418329	100.000	100.000

==== Shimadzu LCsolution 分析报告 ====

样品信息

采集人	:	Admin
样品名称	:	Compound 11
样品 ID	:	
样品架#	:	1
样品瓶#	:	56
进样体积	:	30 uL
数据文件名	:	Compound 11(30%乙腈-TD).lcd
方法文件名	:	XK-30%-乙腈-TD.lcm
批处理文件名	:	XUKUO.lcb
报告文件名	:	XUKUO.lcr
数据采集	:	2019-3-7 08:26:10
数据处理	:	2019-3-7 08:49:13

色谱图
Compound 11 E:\HPLC\绪扩\Compound 11(30%乙腈-TD).lcd

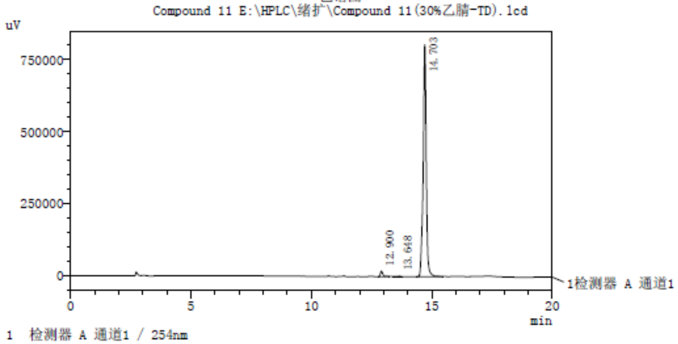

1 检测器 A 通道1 / 254nm

峰表

检测器 A Ch1 254nm

峰#	保留时间	面积	高度	面积 %	高度 %
1	12.900	132829	19136	1.821	2.320
2	13.648	12981	1595	0.178	0.193
3	14.703	7146927	804039	98.001	97.486
总计		7292738	824771	100.000	100.000

==== Shimadzu LCsolution 分析报告 ====

样品信息

采集人	: Admin
样品名称	: Compound 12
样品 ID	:
样品架#	: 1
样品瓶#	: 57
进样体积	: 30 uL
数据文件名	: Compound 12(60%甲醇-TD).lcd
方法文件名	: XK-60%-甲醇-TD.lcm
批处理文件名	: XUKU0.lcb
报告文件名	: XUKU0.lcr
数据采集	: 2019-3-7 13:40:09
数据处理	: 2019-3-7 14:14:13

色谱图
Compound 12 E:\HPLC\绪扩\Compound 12(60%甲醇-TD).lcd

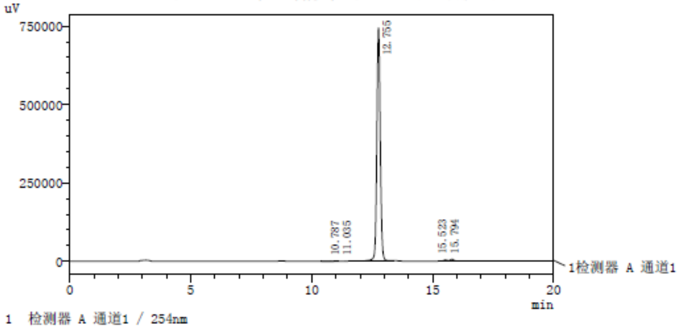

1 检测器 A 通道1 / 254nm

峰表

检测器 A Ch1 254nm

峰#	保留时间	面积	高度	面积 %	高度 %
1	10.787	5375	363	0.073	0.048
2	11.035	729	90	0.010	0.012
3	12.755	7314041	743387	98.672	98.676
4	15.523	33615	3737	0.453	0.496
5	15.794	58699	5784	0.792	0.768
总计		7412458	753362	100.000	100.000

==== Shimadzu LCsolution 分析报告 ====

样品信息

采集人	: Admin
样品名称	: Compound 12
样品 ID	:
样品架#	: 1
样品瓶#	: 57
进样体积	: 30 uL
数据文件名	: Compound 12(30%乙腈-TD).lcd
方法文件名	: XK-30%-乙腈-TD.lcm
批处理文件名	: XUKU0.lcb
报告文件名	: XUKU0.lcr
数据采集	: 2019-3-7 08:49:39
数据处理	: 2019-3-7 09:39:03

色谱图
Compound 12 E:\HPLC\绪扩\Compound 12(30%乙腈-TD).lcd

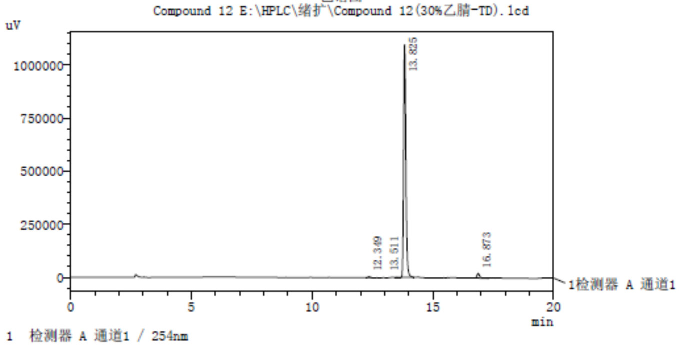

1 检测器 A 通道1 / 254nm

峰表

检测器 A Ch1 254nm

峰#	保留时间	面积	高度	面积 %	高度 %
1	12.349	27276	4568	0.371	0.407
2	13.511	10164	1582	0.138	0.141
3	13.825	7143105	1094695	97.246	97.478
4	16.873	164825	22171	2.244	1.974
总计		7345370	1123016	100.000	100.000

==== Shimadzu LCsolution 分析报告 ====

样品信息

采集人	: Admin
样品名称	: Compound 13
样品 ID	:
样品架#	: 1
样品瓶#	: 58
进样体积	: 30 uL
数据文件名	: Compound 13(60%甲醇-TD).lcd
方法文件名	: XK-60%-甲醇-TD.lcm
批处理文件名	: XUKU0.lcb
报告文件名	: XUKU0.lcr
数据采集	: 2019-3-7 14:03:36
数据处理	: 2019-3-7 14:26:39

色谱图

Compound 13 E:\HPLC\绪扩\Compound 13(60%甲醇-TD).lcd

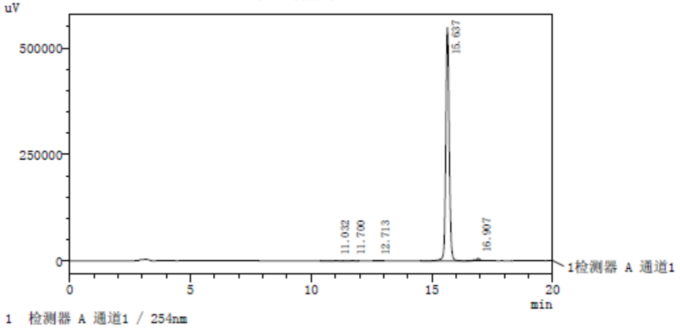

1 检测器 A 通道1 / 254nm

峰表

检测器 A Ch1 254nm

峰#	保留时间	面积	高度	面积 %	高度 %
1	11.032	7976	681	0.145	0.122
2	11.700	8786	801	0.160	0.144
3	12.713	4641	505	0.084	0.091
4	15.637	5409189	548612	98.361	98.573
5	16.907	68745	5952	1.250	1.069
总计		5499337	556551	100.000	100.000

==== Shimadzu LCsolution 分析报告 ====

样品信息

采集人	: Admin
样品名称	: Compound 13
样品 ID	:
样品架#	: 1
样品瓶#	: 58
进样体积	: 30 uL
数据文件名	: Compound 13(30%乙腈-TD).lcd
方法文件名	: XK-30%-乙腈-TD.lcm
批处理文件名	: XUKU0.lcb
报告文件名	: XUKU0.lcr
数据采集	: 2019-3-7 09:13:07
数据处理	: 2019-3-7 09:36:11

色谱图

Compound 13 E:\HPLC\绪扩\Compound 13(30%乙腈-TD).lcd

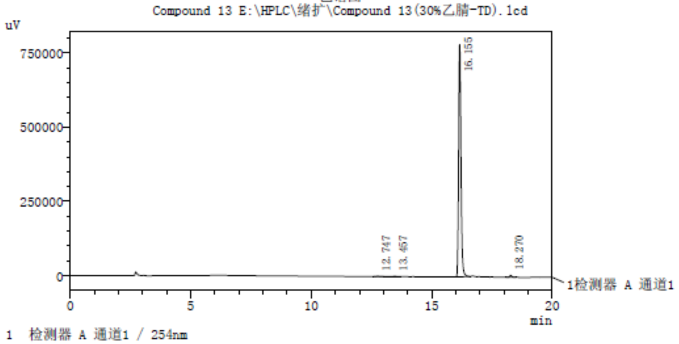

1 检测器 A 通道1 / 254nm

峰表

检测器 A Ch1 254nm

峰#	保留时间	面积	高度	面积 %	高度 %
1	12.747	10622	1321	0.199	0.167
2	13.457	16349	1579	0.306	0.199
3	16.155	5267538	782230	98.729	98.781
4	18.270	40863	6757	0.766	0.853
总计		5335373	791887	100.000	100.000

==== Shimadzu LCsolution 分析报告 ====

样品信息

采集人	: Admin
样品名称	: Compound 14
样品 ID	:
样品架#	: 1
样品瓶#	: 59
进样体积	: 30 uL
数据文件名	: Compound 14(60%甲醇-TD).lcd
方法文件名	: XK-60%-甲醇-TD.lcm
批处理文件名	: XUKU0.lcb
报告文件名	: XUKU0.lcr
数据采集	: 2019-3-7 14:27:05
数据处理	: 2019-3-7 15:04:50

色谱图
Compound 14 E:\HPLC\绪扩\Compound 14(60%甲醇-TD).lcd

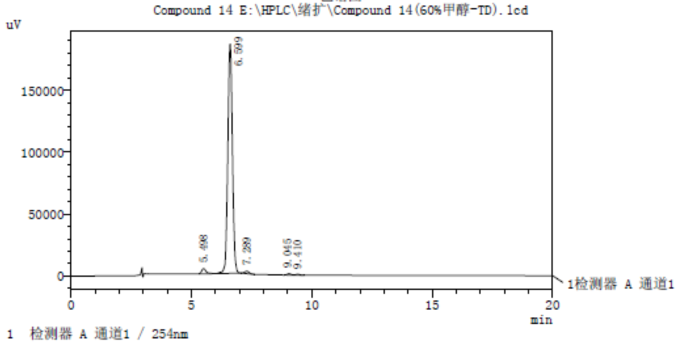

1 检测器 A 通道1 / 254nm

峰表

检测器 A Ch1 254nm

峰#	保留时间	面积	高度	面积 %	高度 %
1	5.498	52485	4192	2.061	2.169
2	6.599	2448759	185088	96.147	95.791
3	7.289	24326	1871	0.955	0.968
4	9.045	12363	1224	0.485	0.633
5	9.410	8955	846	0.352	0.438
总计		2546888	193220	100.000	100.000

==== Shimadzu LCsolution 分析报告 ====

样品信息

采集人	: Admin
样品名称	: Compound 14
样品 ID	:
样品架#	: 1
样品瓶#	: 59
进样体积	: 30 uL
数据文件名	: Compound 14(30%乙腈-TD).lcd
方法文件名	: XK-30%-乙腈-TD.lcm
批处理文件名	: XUKU0.lcb
报告文件名	: XUKU0.lcr
数据采集	: 2019-3-7 09:36:38
数据处理	: 2019-3-7 09:59:41

色谱图
Compound 14 E:\HPLC\绪扩\Compound 14(30%乙腈-TD).lcd

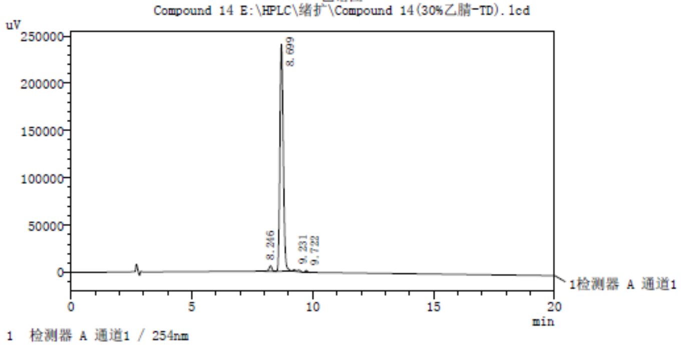

1 检测器 A 通道1 / 254nm

峰表

检测器 A Ch1 254nm

峰#	保留时间	面积	高度	面积 %	高度 %
1	8.246	49778	6276	2.053	2.512
2	8.699	2338006	239956	96.424	96.061
3	9.231	25096	1618	1.035	0.648
4	9.722	11825	1946	0.488	0.779
总计		2424705	249796	100.000	100.000

==== Shimadzu LCsolution 分析报告 ====

样品信息

采集人	: Admin
样品名称	: Compound 15
样品 ID	:
样品架#	: 1
样品瓶#	: 60
进样体积	: 30 uL
数据文件名	: Compound 15(60%甲醇-TD).lcd
方法文件名	: XK-60%-甲醇-TD.lcm
批处理文件名	: XUKUO.lcb
报告文件名	: XUKUO.lcr
数据采集	: 2019-3-7 14:50:34
数据处理	: 2019-3-7 15:13:37

色谱图
Compound 15 E:\HPLC\绪扩\Compound 15(60%甲醇-TD).lcd

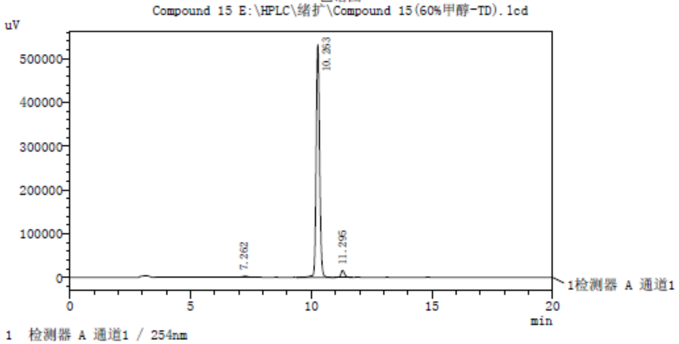

1 检测器 A 通道1 / 254nm

峰表

检测器 A Ch1 254nm

峰#	保留时间	面积	高度	面积 %	高度 %
1	7.262	33673	2342	0.617	0.425
2	10.263	5262266	532148	96.418	96.661
3	11.295	161821	16040	2.965	2.914
总计		5457760	550530	100.000	100.000

==== Shimadzu LCsolution 分析报告 ====

样品信息

采集人	: Admin
样品名称	: Compound 15
样品 ID	:
样品架#	: 1
样品瓶#	: 60
进样体积	: 30 uL
数据文件名	: Compound 15(30%乙腈-TD).lcd
方法文件名	: XK-30%-乙腈-TD.lcm
批处理文件名	: XUKUO.lcb
报告文件名	: XUKUO.lcr
数据采集	: 2019-3-7 10:00:08
数据处理	: 2019-3-7 10:23:11

色谱图
Compound 15 E:\HPLC\绪扩\Compound 15(30%乙腈-TD).lcd

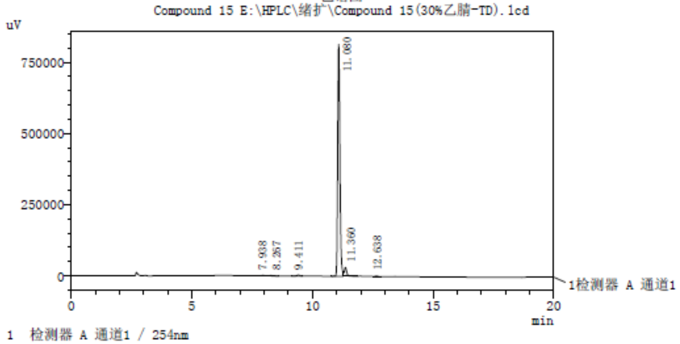

1 检测器 A 通道1 / 254nm

峰表

检测器 A Ch1 254nm

峰#	保留时间	面积	高度	面积 %	高度 %
1	7.938	10989	1305	0.203	0.153
2	8.267	10109	1153	0.187	0.135
3	9.411	29400	3743	0.543	0.439
4	11.080	5138440	814435	94.880	95.411
5	11.360	212481	30782	3.923	3.606
6	12.638	14292	2192	0.264	0.257
总计		5415712	853609	100.000	100.000

==== Shimadzu LCsolution 分析报告 ====

样品信息

采集人	: Admin
样品名称	: Compound 16
样品 ID	:
样品架#	: 1
样品瓶#	: 61
进样体积	: 30 uL
数据文件名	: Compound 16(60%甲醇-TD).lcd
方法文件名	: XK-60%-甲醇-TD.lcm
批处理文件名	: XUKU0.lcb
报告文件名	: XUKU0.lcr
数据采集	: 2019-3-7 15:14:02
数据处理	: 2019-3-7 15:37:05

色谱图

Compound 16 E:\HPLC\绪扩\Compound 16(60%甲醇-TD).lcd

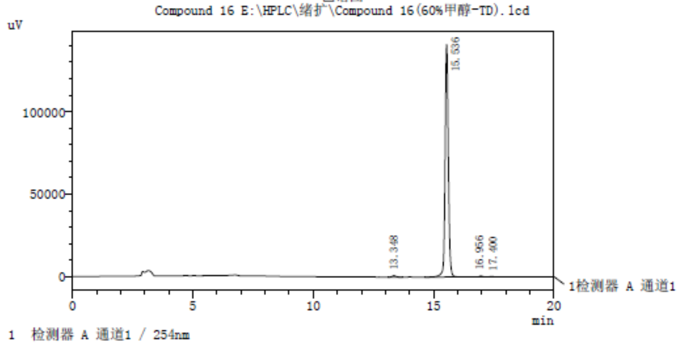

1 检测器 A 通道1 / 254nm

峰表

检测器 A Ch1 254nm

峰#	保留时间	面积	高度	面积 %	高度 %
1	13.348	8740	877	0.655	0.614
2	15.536	1319331	141182	98.870	98.783
3	16.956	4357	612	0.327	0.428
4	17.400	1978	250	0.148	0.175
总计		1334405	142921	100.000	100.000

==== Shimadzu LCsolution 分析报告 ====

样品信息

采集人	: Admin
样品名称	: Compound 16
样品 ID	:
样品架#	: 1
样品瓶#	: 61
进样体积	: 30 uL
数据文件名	: Compound 16(30%乙腈-TD).lcd
方法文件名	: XK-30%-乙腈-TD.lcm
批处理文件名	: XUKU0.lcb
报告文件名	: XUKU0.lcr
数据采集	: 2019-3-7 10:23:36
数据处理	: 2019-3-7 10:46:39

色谱图

Compound 16 E:\HPLC\绪扩\Compound 16(30%乙腈-TD).lcd

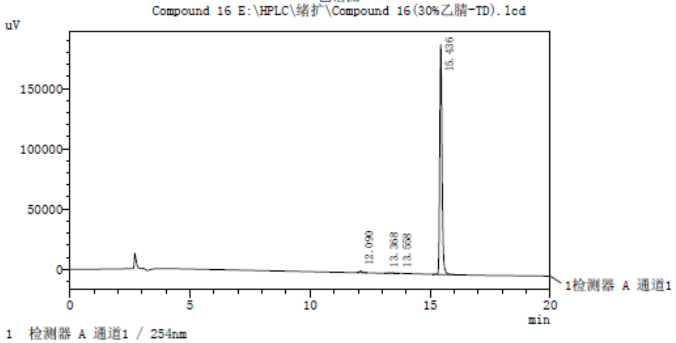

1 检测器 A 通道1 / 254nm

峰表

检测器 A Ch1 254nm

峰#	保留时间	面积	高度	面积 %	高度 %
1	12.090	8497	1374	0.662	0.713
2	13.368	3994	273	0.311	0.142
3	13.558	1078	182	0.084	0.094
4	15.436	1269881	190811	98.943	99.051
总计		1283450	192639	100.000	100.000

==== Shimadzu LCsolution 分析报告 ====

样品信息

采集人	: Admin
样品名称	: Compound 17
样品 ID	:
样品架#	: 1
样品瓶#	: 62
进样体积	: 30 uL
数据文件名	: Compound 17(30%乙腈-TD).lcd
方法文件名	: XK-30%-乙腈-TD.lcm
批处理文件名	: XUKU0.lcb
报告文件名	: XUKU0.lcr
数据采集	: 2019-3-7 10:47:04
数据处理	: 2019-3-7 14:36:58

色谱图
Compound 17 E:\HPLC\绪扩\Compound 17(30%乙腈-TD).lcd

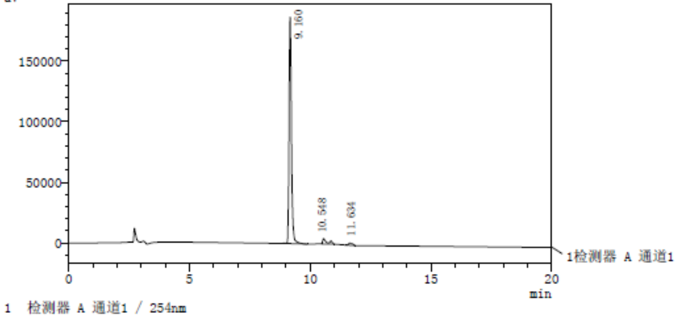

1 检测器 A 通道1 / 254nm

峰表

检测器 A Ch1 254nm

峰#	保留时间	面积	高度	面积 %	高度 %
1	9.160	1300054	186783	94.882	96.958
2	10.548	50836	4076	3.710	2.116
3	11.634	19286	1784	1.408	0.926
总计		1370175	192644	100.000	100.000

==== Shimadzu LCsolution 分析报告 ====

样品信息

采集人	: Admin
样品名称	: Compound 17
样品 ID	:
样品架#	: 1
样品瓶#	: 62
进样体积	: 30 uL
数据文件名	: Compound 17(60%甲醇-TD).lcd
方法文件名	: XK-60%-甲醇-TD.lcm
批处理文件名	: XUKU0.lcb
报告文件名	: XUKU0.lcr
数据采集	: 2019-3-7 15:37:31
数据处理	: 2019-3-7 16:00:34

色谱图
Compound 17 E:\HPLC\绪扩\Compound 17(60%甲醇-TD).lcd

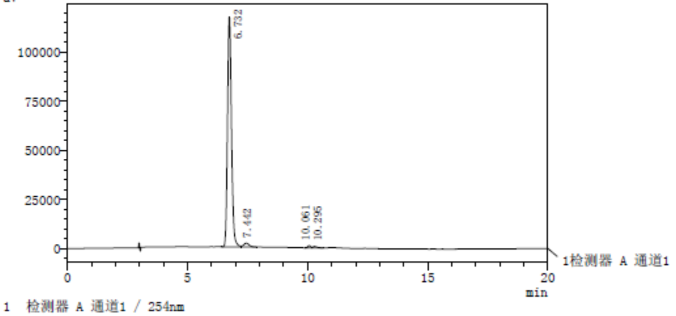

1 检测器 A 通道1 / 254nm

峰表

检测器 A Ch1 254nm

峰#	保留时间	面积	高度	面积 %	高度 %
1	6.732	1349366	117237	96.535	96.820
2	7.442	29419	1928	2.105	1.592
3	10.061	10540	1156	0.754	0.955
4	10.295	8477	766	0.606	0.633
总计		1397803	121087	100.000	100.000

==== Shimadzu LCsolution 分析报告 ====

样品信息

采集人	: Admin
样品名称	: Compound 18
样品 ID	:
样品架#	: 1
样品瓶#	: 63
进样体积	: 30 uL
数据文件名	: Compound 18(60%甲醇-TD).lcd
方法文件名	: XK-60%-甲醇-TD.lcm
批处理文件名	: XUKUO.lcb
报告文件名	: XUKUO.lcr
数据采集	: 2019-3-7 16:00:59
数据处理	: 2019-3-7 16:27:37

色谱图
Compound 18 E:\HPLC\绪扩\Compound 18(60%甲醇-TD).lcd

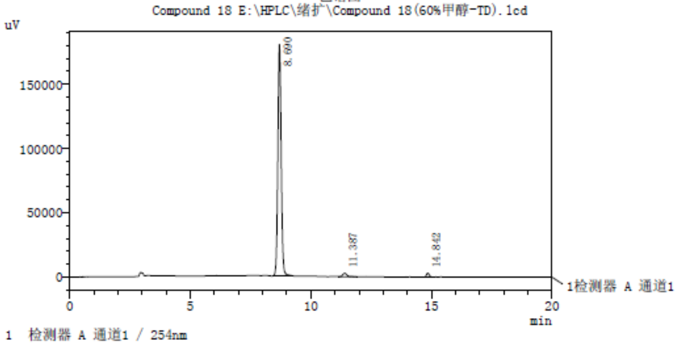

1 检测器 A 通道1 / 254nm

峰表

检测器 A Ch1 254nm

峰#	保留时间	面积	高度	面积 %	高度 %
1	8.690	1733559	180215	96.752	96.830
2	11.387	32621	2754	1.821	1.480
3	14.842	25567	3146	1.427	1.690
总计		1791747	186115	100.000	100.000

==== Shimadzu LCsolution 分析报告 ====

样品信息

采集人	: Admin
样品名称	: Compound 18
样品 ID	:
样品架#	: 1
样品瓶#	: 63
进样体积	: 30 uL
数据文件名	: Compound 18(30%乙腈-TD).lcd
方法文件名	: XK-30%-乙腈-TD.lcm
批处理文件名	: XUKUO.lcb
报告文件名	: XUKUO.lcr
数据采集	: 2019-3-7 11:10:32
数据处理	: 2019-3-7 11:33:33

色谱图
Compound 18 E:\HPLC\绪扩\Compound 18(30%乙腈-TD).lcd

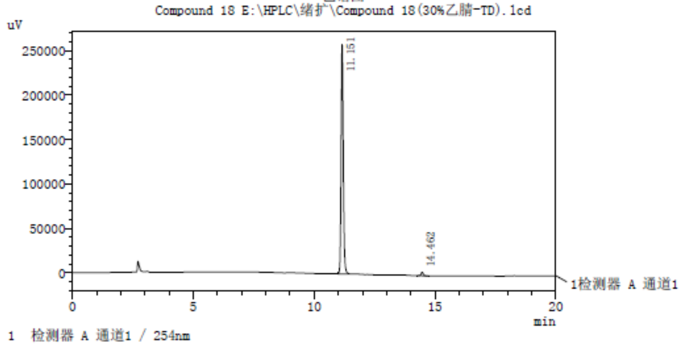

1 检测器 A 通道1 / 254nm

峰表

检测器 A Ch1 254nm

峰#	保留时间	面积	高度	面积 %	高度 %
1	11.151	1593630	257530	98.422	98.476
2	14.462	25553	3986	1.578	1.524
总计		1619183	261515	100.000	100.000
